# NAE1/UBA3-UBE2M are E1 and E2 enzymes for the URM1 modification

**DOI:** 10.1038/s41467-026-72296-w

**Published:** 2026-04-29

**Authors:** Swatadipta Chakraborty, Saibal Chanda, Zhongwen Cao, Alan Pham, Zihan Zhang, Yinsheng Wang, Logan Herring, Wenyue Cao, Wenshe Ray Liu

**Affiliations:** 1 Department of Biochemistry and Biophysics, College of Agriculture and Life Sciences, Texas A&M University, College Station, TX USA; 2 Environmental Toxicology Graduate Program, University of California, Riverside, CA USA; 3 Texas A&M Drug Discovery Center, Texas A&M University, College Station, TX USA; 4 Department of Chemistry, College of Arts and Sciences, Texas A&M University, College Station, TX USA; 5 Institute of Biosciences and Technology, Texas A&M University, Houston, TX USA; 6 Department of Translational Medical Sciences, Naresh K. Vashisht College of Medicine, Texas A&M University, Houston, TX USA; 7 Department of Chemistry, University of California, Riverside, CA USA; 8 Department of Cell Biology and Genetics, Naresh K. Vashisht College of Medicine, Texas A&M University, College Station, TX USA; 9 Department of Pharmaceutical Sciences, Irma Lerma Rangel College of Pharmacy, Texas A&M University, College Station, TX USA

**Keywords:** Neddylation, Proteomics

## Abstract

Ubiquitin-related modifier 1 (URM1) is an evolutionarily conserved ubiquitin-like protein. In eukaryotes, it serves dual roles as a sulfur donor for tRNA modification and a posttranslational protein modifier. URM1 is proposed to be a primitive protein modifier and a potential precursor to the more complex ubiquitin system. However, no specific activating enzyme (E1), conjugating enzyme (E2), or ligase (E3) has been reported for the URM1 modification cascade in human cells. In this study, we design an activity-based URM1 probe to covalently capture cysteine enzymes functioning in the URM1 signaling pathway. Through proteomic characterization and cell-based validation, we identify NAE1/UBA3 and UBE2M as E1 and E2 enzymes, respectively, for the urmylation pathway under both normal and oxidative stress conditions. Pharmacologic perturbation of the UBE2M–DCN1 module suggests DCN1 may contribute to URM1 conjugation. Bioinformatic analysis further reveals that genetic knockdown of NAE1, UBE2M, and URM1 affects overlapping genes associated with pathways controlling cellular response to stress conditions or with implications in liver diseases. URM1 serves a protective role against oxidative stress. Pevonedistat, a potent NAE1 inhibitor that blocks protein urmylation in human cells, exhibits strong synergy with cisplatin, an agent known to induce oxidative stress, in killing liver cancer cells effectively.

## Abstract (web summary)

URM1 is an ancient protein modifier whose mechanism in human cells has been unclear. Here, the authors show that NAE1/UBA3 and UBE2M drive URM1 modification, especially during oxidative stress, and that pathway inhibition sensitizes liver cancer cells to cisplatin.

## Introduction

**Fig. 1:**
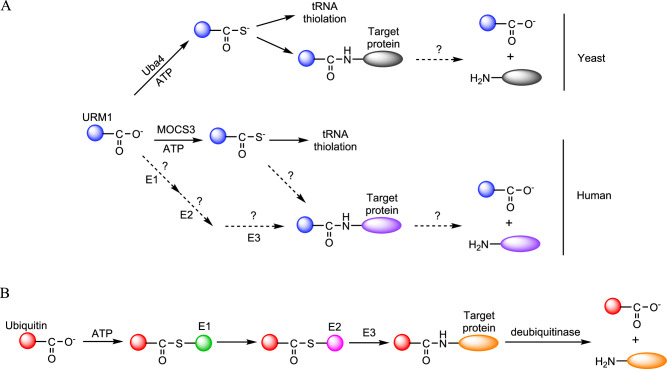
Urmylation and ubiquitination pathways. **A** Urmylation pathways in yeast and human cells. In yeast, URM1 is activated by Uba4 to form a thiocarboxylate intermediate, which serves dual roles in tRNA thiolation and protein urmylation. In human cells, it is known that MOCS3 catalyzes the formation of URM1 thiocarboxylate. **B** For comparison, the canonical E1-E2-E3 catalytic cascade of protein ubiquitination is shown.

Ubiquitin-like proteins (UBLs) are a group of small proteins that modify other proteins in cells. They are structurally similar to ubiquitin and are involved in many cellular processes, including DNA replication and repair, transcription regulation, protein homeostasis, and autophagy^1–3^. Ubiquitin-related modifier 1 (URM1) is a member of the UBL family. It shares structural features with ubiquitin, including a conserved β-grasp fold and a *C*-terminal di-glycine motif for conjugating with target proteins. However, URM1 is distinct in its dual functionality by serving as both a sulfur donor in tRNA thiolation and a protein modifier in response to oxidative stress^4–6^. URM1 was first discovered in yeast. As illustrated in Fig. 1A, it is activated in yeast by Uba4, an E1-like enzyme, to conjugate with a variety of proteins^7–9^. Although it was suggested that E2 and E3 enzymes exist in the urmylation pathway^10^, no such enzymes have been discovered in yeast. Instead, a proteomic analysis revealed that URM1 undergoes an unusual activation process in yeast by forming a thiocarboxylate intermediate^4^. This intermediate serves two distinct roles by both transferring sulfur for tRNA thiolation and directly modifying target proteins at their lysine residues^11–15^. In yeast, stress conditions promote increased protein urmylation that drives the formation of phase-separated assemblies with an apparent role in stress resilience^16^. In human cells, MOCS3 is a Uba4 homolog that catalyzes the synthesis of URM1 thiocarboxylate for tRNA thiolation^17^. However, a direct connection between URM1 thiocarboxylate and protein urmylation in human cells has not been established.

It was found that reduction in URM1 levels induces severe cytokinesis defects in HeLa cells, leading to the accumulation of enlarged multinucleated cells, which highlights the critical role of protein urmylation in cell cycle regulation^4^. Under oxidative stress, protein urmylation in HeLa cells is significantly elevated, suggesting a potential protective role against oxidative damage^6^. There is also a study linking URM1 to tumor growth promotion and apoptosis suppression via the JNK signaling pathway in hepatocellular carcinoma (HCC)^18^. Recent work further supports that the characteristic C-terminal GG motif is not strictly required for URM1 conjugation in certain settings, underscoring mechanistic divergence from canonical UBLs^19^. Beyond these, little is known about URM1’s functions in mammalian cells. This gap in knowledge leaves a critical question unanswered. The precise mechanism by which URM1 is conjugated to lysine residues on target proteins in mammalian cells remains unclear. In particular, it is unknown whether this occurs through a thiocarboxylate intermediate or through a canonical E1–E2–E3 cascade, as in protein ubiquitination illustrated in Fig. 1B. Filling this knowledge gap holds high significance, as CRISPR-Cas9 knockout of URM1 has been shown to reduce fitness across 468 cancer cell lines and 39 cancer types, highlighting the biological significance of urmylation pathway and its potential as a drug target^20^.

In this work, we employ an activity-based URM1 probe to covalently capture cysteine enzymes involved in the human urmylation pathway. Using proteomics and orthogonal validation, we demonstrate that URM1 activation and transfer in human cells proceed through the NAE1/UBA3–UBE2M axis, particularly under oxidative stress, thereby defining a mechanistically distinct route for mammalian protein urmylation.

## Results

### The synthesis of FLAG-URM1-G101Pa as an activity-based probe

**Fig. 2:**
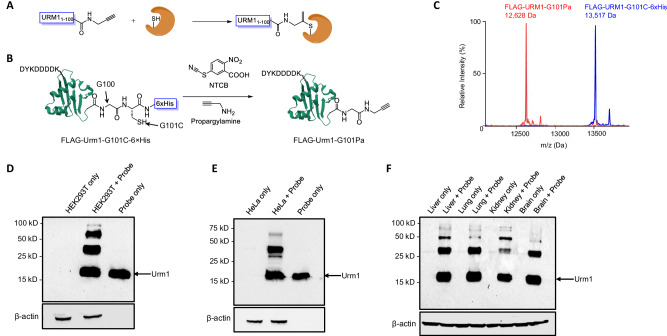
Synthesis and characterization of FLAG-URM1-G101Pa and its use in activity-based protein profiling of cysteine enzymes functioning in the URM1 signaling pathway. **A** A proposed covalent reaction between a propargylamine-containing URM1 ABP with a cysteine enzyme that generates a stable vinyl thioether complex. **B** A diagram illustrating the synthesis of FLAG-URM1-G101Pa from recombinantly expressed FLAG-URM1-G101C-6×His using the activated cysteine-based protein ligation (ACPL) technique. **C** Deconvoluted electrospray ionization mass spectrometry (ESI-MS) spectra of recombinantly expressed FLAG-URM1-G101C-6×His and synthesized FLAG-URM1-G101Pa. Theoretical molecular weights for FLAG-URM1-G101C-6×His and FLAG-URM1-G101Pa are 13,517 and 12,628 Da, respectively. **D** Cysteine enzymes in HEK293T cells probed by FLAG-URM1-G101Pa. **E** Cysteine enzymes in HeLa cells probed by FLAG-URM1-G101Pa. **F** Cysteine enzymes in four homogenized mouse tissues probed by FLAG-URM1-G101Pa. In **D**–**F**, Lysates of HEK293T cells, HeLa cells, or homogenized mouse tissues including liver, lung, kidney, and brain were incubated with FLAG-URM1-G101Pa (labeled as “Probe” in **D**–**F**) at 4 °C overnight, immunoprecipitated using anti-FLAG magnetic agarose resins, separated by SDS-PAGE, and then probed by an anti-FLAG antibody in Western blot analysis. Same gels were probed by an anti-β-actin antibody as loading controls. No. of independent experiments, in **D**–**F**, *n* = 3. Source data are provided as a Source Data file.

The activity-based protein profiling technique is a powerful proteomics tool for investigating enzyme-substrate interactions in complex biological systems^21^. In this technique, activity-based probes (ABPs) are designed to covalently label active site residues of target enzymes, enabling their selective enrichment and characterization through proteomic analysis^22,23^. Many enzymes involved in ubiquitin and UBL signaling pathways, including E1 activating enzymes, E2 conjugating enzymes, some E3 ligases, and most deconjugating proteases, utilize cysteine residues as catalytic sites^24,25^. Consequently, ABPs for these enzymes can be engineered by replacing the *C*-terminal glycine of ubiquitin or UBLs with an electrophilic warhead that can form covalent interactions with catalytic cysteines. Building upon this concept, researchers have developed ABPs based on ubiquitin, ubiquitin-like modifiers (SUMOs), and ISG15^26–32^. In particular, ubiquitin ABPs have been extensively used to profile a wide range of deubiquitinases, many of which function as cysteine proteases. One of the most currently used ubiquitin ABPs is ubiquitin_1-75_ propargylamine (UbPa). UbPa was synthesized by Ovaa et al. for a different purpose but was found to be highly reactive towards catalytic cysteines of deubiquitinases^33^. Given the high structural similarity between ubiquitin and URM1, we reasoned that replacing URM1’s *C*-terminal glycine (G101) with propargylamine would generate a URM1 ABP, as illustrated in Fig. 2A, capable of covalently capturing cysteine enzymes involved in the urmylation pathway by forming vinyl thioether complexes. Here, the propargylamide serves primarily as a covalent capture handle, whereas apparent specificity is driven mainly by the URM1 scaffold and active-site engagement. To facilitate the detection and enrichment of captured enzymes, a commonly used FLAG tag (DYKDDDDK) was fused to the *N*-terminus of URM1. We named this designed URM1 ABP as FLAG-URM1-G101Pa (Fig. 2B).

The expressed protein ligation technique, which involves recombinantly producing a protein with an intein fused to its *C*-terminus to form a protein thioester, has been commonly used to generate ubiquitin thioesters for subsequent reactions with small molecules to afford ubiquitin, SUMO, and ISG15 ABPs^34,35^. An alternative expressed protein ligation technique, which bypasses the need for intein or enzymatic involvement, has also been developed and applied to synthesize ubiquitin- and UBL-small molecule conjugates^36^. This method, known as activated cysteine-based protein ligation (ACPL), utilizes a cyano donor molecule, such as 2-nitro-5-thiocyanobenzoic acid (NTCB), to activate a protein cysteine (Fig. 2B) for an exchange reaction with a small molecule amine. This reaction ultimately replaces the cysteine residue with the small molecule amine at the *C*-terminus of the target protein. Due to its simplicity, we chose to use ACPL to synthesize FLAG-URM1-G101Pa. FLAG-URM1-G101C-6×His was expressed in *E. coli* and purified to homogeneity using Ni-NTA affinity and size exclusion chromatography. We then incubated FLAG-URM1-G101C-6×His with 5 mM NTCB and 500 mM propargylamine at 37 °C overnight to afford FLAG-URM1-G101Pa^37^. The ligation product was purified by desalting in a fast protein liquid chromatography (FPLC) system and then run through Ni-NTA resins to remove unreacted original protein. Electrospray ionization-mass spectrometry (ESI-MS) analysis of purified product showed a deconvoluted mass peak at 12,628 Da that was consistent with its theoretical molecular weight (Fig. 2C, Supplementary Fig. 1). Analytical LC–MS further confirmed efficient conversion and high purity of FLAG-URM1-G101Pa (Supplementary Fig. 1E). We observed a tendency of polymerization for FLAG-URM1-G101C-6×His, which was reduced in Flag-URM1-G101Pa due to the absence of free cysteine residues.

### Capturing URM1-targeting cysteine enzymes in cell and tissue lysates using FLAG-URM1-G101Pa

After confirming successful synthesis of FLAG-URM1-G101Pa, we applied it to covalently capture URM1-targeting cysteine enzymes in human cells. HEK293T cells were selected for this study due to their well-characterized expression of various deubiquitinases and other cysteine enzymes^38^. HeLa cells were also included in this study as many cysteine proteases are frequently overexpressed in cancer^39^. Cell lysates for the two cell lines were incubated with 6 μM FLAG-URM1-G101Pa in the presence of 1 mM tris(2-caroxyethyl)phosphine (TCEP) at 4 °C overnight. Coimmunoprecipitation was then performed using anti-FLAG magnetic resin beads to selectively isolate FLAG-URM1-G101Pa-bound proteins from the reaction mixtures. Immunoprecipitated proteins were separated by sodium dodecyl sulfate-polyacrylamide gel electrophoresis (SDS-PAGE) and probed by an anti-FLAG antibody in Western blot analysis. Cell lysates incubated with 1 mM TCEP alone at 4 °C overnight, followed by identical processing and analysis, served as the control. The URM1 ABP (FLAG-URM1-G101Pa) itself was also included in SDS-PAGE and Western blot analysis as a control. Protein loading comparison was made by analyzing β-actin in loaded samples. Final Western blot results are presented in Fig. 2D, E, and Supplementary Fig. 2A–C. As shown in these figures, cell lysates incubated with FLAG-URM1-G101Pa exhibited several higher molecular weight bands that were absent in both controls, indicating that the probe successfully labeled proteins in both cell lysates to form covalent protein complexes. These findings strongly suggest the presence of multiple cysteine enzymes involved in the URM1 signaling pathway in human cells.

Enzymes in the ubiquitin pathway are well known with varied expression patterns in different mammalian tissues^40^. We suspected that URM1-targeting enzymes would also have varied expression patterns in different tissues. Encouraged by enzyme capturing results in HEK293T and HeLa cell lysates, we proceeded to use FLAG-URM1-G101Pa to test this aspect in different mouse tissues. Mouse tissues including liver, lung, kidney, and brain were dissected and immediately stored on dry ice. Homogenized tissues from 20 to 50 mg tissue samples were clarified and supernatants were then used to react with FLAG-URM1-G101Pa in the presence of 1 mM TCEP at 4 °C overnight. Final reaction mixtures were immunoprecipitated using anti-FLAG magnetic agarose beads and immunoprecipitated proteins were then separated by SDS-PAGE and probed by anti-FLAG in Western blot analysis. Tissue lysates treated only with 1 mM TCEP and processed similarly were used as controls. Final Western blot results are presented in Fig. 2F and Supplementary Fig. 2D. As shown in these figures, distinct labeling patterns were observed across these tissues, with liver, lung, and kidney lysates exhibiting additional bands compared to brain lysates. Protein complexes that were detected around and above 100 kDa in liver, lung, and kidney lysates are obviously different from each other, indicating that different URM1-targeting cysteine enzymes are expressed in these tissues. Collectively, results obtained so far support the notion that FLAG-URM1-G101Pa can serve as an ABP to capture URM1-targeting cysteine enzymes in cell and tissue lysates.

### Proteomic analysis of cysteine enzymes profiled by FLAG-URM1-G101Pa from HEK293T and HeLa cell lysates

**Fig. 3:**
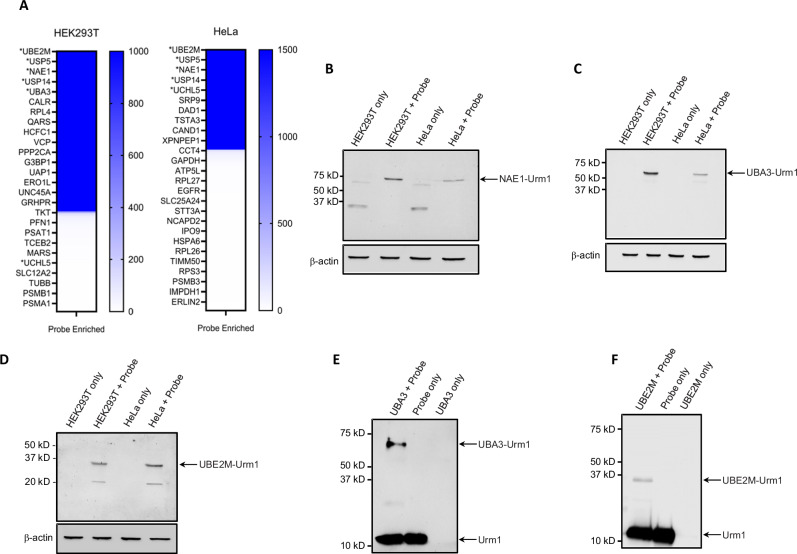
Proteomic analysis of proteins enriched by FLAG-URM1-G101Pa from HEK293T and HeLa cell lysates and validation of NAE1, UBA3, and UBE2M as binders for FLAG-URM1-G101Pa. **A** Heatmaps of FLAG-URM1-G101Pa-enriched protein candidates in HEK293T and HeLa cells. The ratios shown as side bars were calculated by averaging data from four replicates based on detected label-free quantification (LFQ) intensity. Enzymes known in ubiquitin or UBL pathways are asterisked. **B**–**D** Immunoprecipitation to enrich NAE1, UBA3, and UBE2M from FLAG-URM1-G101Pa-treated HEK293T and HeLa cell lysates. Cell lysates were incubated with FLAG-URM1-G101Pa at 4 °C overnight, immunoprecipitated by anti-FLAG magnetic agarose resins, separated by SDS-PAGE, and then detected by an anti-NAE1 (**B**), anti-UBA3 (**C**), or anti-UBE2M (**D**) antibody in Western blot analysis. Cell lysates without treatment with FLAG-URM1-G101Pa were used as negative controls. **E** Formation of a UBA3-URM1 complex by directly incubating UBA3 with FLAG-URM1-G101Pa. **F** Formation of a UBE2M-URM1 complex by directly incubating UBE2M with FLAG-URM1-G101Pa. Reaction mixtures in **E**, **F** were separated by SDS-PAGE and probed by anti-FLAG in Western blot analysis. No. of independent experiments, in **B**–**F**, *n* = 3. Source data are provided as a Source Data file.

To characterize cysteine enzymes that can be covalently labeled with the synthesized URM1 ABP, we first reacted HEK293T and HeLa cell lysates separately with FLAG-URM1-G101Pa by following the same procedure discussed in the previous section and then immunoprecipitated probe-conjugated proteins using anti-FLAG magnetic agarose beads. Isolated proteins were then separated by SDS-PAGE. SDS-PAGE gel regions corresponding to high molecular weight bands observed in Western blots were excised and subjected to trypsin digestion. We analyzed digested peptides for identifying their corresponding proteins via liquid chromatography with tandem mass spectrometry (LC-MS/MS) using an Orbitrap Fusion Lumos mass spectrometer (Supplementary Tables 1 and 2; Supplementary Fig. 3). For meaningful comparison in label-free quantification (LFQ-MS) proteomics, HEK293T and HeLa cell lysates were treated with FLAG-URM1-G101C-6×His, processed similarly, and analyzed as controls. Using FLAG-URM1-G101C-6×His-treated samples as controls avoided potential identifications of non-covalent URM1 and FLAG binders. This proteomic analysis revealed multiple enzymes that preferentially bind to FLAG-URM1-G101Pa with notable enrichment in both HEK293T and HeLa cell lysates, compared to FLAG-URM1-G101C-6×His-treated samples. As shown in Fig. 3A, key enzymes identified included NEDD8-activating enzyme (NAE) E1 regulatory subunit NAE1 (Protein ID: 13564), E2 conjugating enzyme UBE2M that is also known as Ubc12 (Protein ID: 61081), and deubiquitinases such as USP5 (Protein ID: 45974), USP14 (Protein ID: 54578), and UCHL5 (Protein ID: 9Y5K5). Although not shown in FLAG-URM1-G101Pa-treated HeLa cell lysates, NAE E1 catalytic subunit UBA3 (Protein ID: Q8TBC4) was highly enriched in FLAG-URM1-G101Pa-treated HEK293T cell lysates. Since NAE1 and UBA3 are two tightly bound subunits in a catalytically active NAE complex, their co-discovery is anticipated. The majority of probe-enriched proteins from HEK293T cells were also discovered in probe-enriched HeLa cell samples as shown in Fig. 3A.

There are also other protein classes identified. To align with our research objectives, we chose to focus primarily on cysteine enzymes and their validations. USP5, USP14, and UCHL5 are deubiquitinases with high catalytic activities. Given structural similarities between URM1 and ubiquitin, covalent interactions between these enzymes and FLAG-URM1-G101Pa and their proteomic identifications from URM1 ABP-treated HEK293T and HeLa cell lysates were not a surprise. To validate them as deconjugating proteases for protein urmylation, a fluorogenic URM1-ACA (ACA: 7-amino-4-coumarinyl-acetic acid) substrate was synthesized by running the ACPL reaction between FLAG-URM1-G101C-6×His and Gly-ACA. All three enzymes efficiently hydrolyzed URM1-ACA in vitro, consistent with cross-reactivity expected from URM1/ubiquitin structural similarity; yet whether they function as physiological de-urmylating enzymes remains to be determined (Supplementary Fig. 4). However, identifications of NAE1/UBA3 and UBE2M that are E1 and E2 enzymes, respectively, especially UBE2M, are completely unexpected. If the urmylation pathway in human cells mirrors the mechanism observed in yeast, an E2 enzyme will not be necessary. Otherwise, a canonical E1-E2-E3 cascade may exist. Uncovering this fundamental mechanism holds significant implications for our understanding of the urmylation pathway in humans. Recognizing the potential impact of this discovery on cell biology, we prioritized the validation of these two enzymes in the human urmylation pathway for further investigation.

### Validation of NAE1/UBA3 and UBE2M as protein binders for FLAG-URM1-G101Pa

To validate NAE1/UBA3 and UBE2M as URM1-targeting enzymes, we incubated HEK293T and HeLa cell lysates with FLAG-URM1-G101Pa, followed by immunoprecipitation using anti-FLAG magnetic agarose beads. The precipitated proteins were then separated by SDS-PAGE and analyzed via Western blotting using anti-NAE1, anti-UBA3, and anti-UBE2M antibodies. As controls, untreated cell lysates were processed in the same manner. As shown in Fig. 3B, anti-NAE1 detected a distinct band around 75 kDa in probe-treated HEK293T and HeLa cell lysates. This band was absent in untreated controls. While NAE1 is a regulatory subunit in the NAE1/UBA3 E1 complex, this observed band corresponds to the size of an NAE1-URM1 complex. In two negative controls, a 60 kDa background band was detected, likely representing free NAE1, and a 25 kDa background band may result from non-specific antibody binding. Figure 3C further confirms UBA3 enrichment in probe-treated and immunoprecipitated samples. Anti-UBA3 detected a clear UBA3-URM1 complex around 60 kDa, with minimal background in two untreated samples. For UBE2M, Fig. 3D shows that its complex with FLAG-URM1-G101Pa was strongly detected in probe-treated and immunoprecipitated samples, appearing as a 30 kDa band. This band was absent in both negative controls. Additionally, a 20 kDa band was observed in probe-treated samples, corresponding to the size of free UBE2M. Since this band was absent in the controls, it is likely a dissociation product of the UBE2M-URM1 complex that was generated during SDS-PAGE analysis. To confirm the formation of covalent complexes between FLAG-URM1-G101Pa and its binding partners, biochemical assays were conducted as well. UBA3 was incubated with FLAG-URM1-G101Pa at 37 °C for 45 min, followed by SDS-PAGE and Western blot analysis with anti-FLAG. Figure 3E shows a 60 kDa band, corresponding to the UBA3-URM1 complex, which was absent in both negative controls (probe-only and UBA3-only samples). A similar reaction was performed for UBE2M and FLAG-URM1-G101Pa. As shown in Fig. 3F, a 30 kDa UBE2M-URM1 complex band was clearly detected with anti-FLAG, whereas no such band was observed in probe-only and UBE2M-only samples. Collectively, these results demonstrate that the NAE1/UBA3 complex and UBE2M covalently interact with FLAG-URM1-G101Pa, supporting them as potential key enzymes in the URM1 modification pathway in human cells. However, these data do not exclude contributions from thiocarboxylate-dependent activation routes described for URM1 biology, and the relative importance of these mechanisms in cells under oxidative stress remains to be determined.

### Validation of NAE1/UBA3 and UBE2M as E1 and E2 enzymes in the URM1 modification pathway in human cells by gene silencing

**Fig. 4:**
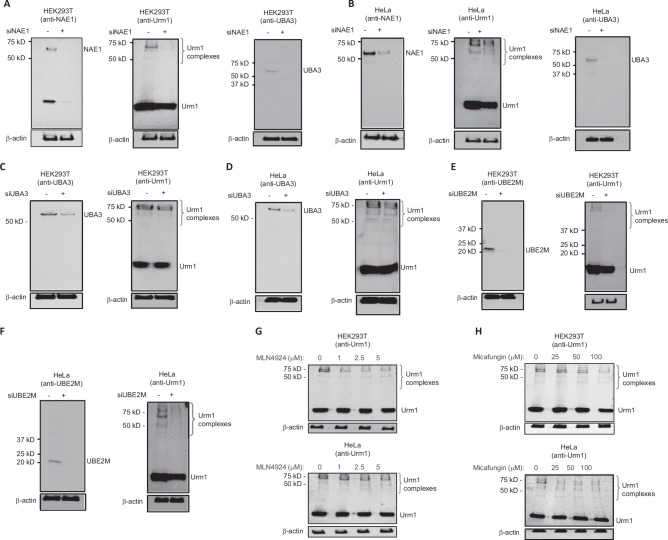
Validation of NAE1/UBA3 and UBE2M as E1 and E2 enzymes in the URM1 modification in human cells. siRNA knockdown of NAE1 probed by anti-NAE1 in HEK293T cells (**A**) and HeLa cells (**B**) and siNAE1-mediated downregulation of overall protein urmylation detected by an anti-URM1 antibody and UBA3 expression detected by anti-UBA3. siRNA knockdown of UBA3 probed by anti-UBA3 in HEK293T (**C**) and HeLa (**D**) cells and siUBA3-elicited downregulation of overall protein urmylation detected by anti-URM1. siRNA knockdown of UBE2M probed by anti-UBE2M in HEK293T (**E**) and HeLa (**F**) cells and the ensuing downregulation of overall protein urmylation detected by anti-URM1. Inhibition of overall protein urmylation in HEK293T and HeLa cells by MLN4924 (**G**) and micafungin (**H**), which are inhibitors of NAE and UBE2M, respectively. Samples in **A**–**H** has been resolved by native-PAGE. No. of independent experiments, in A-H, *n* = 3. Source data are provided as a Source Data file.

Gene silencing by RNA interference (RNAi) is a widely used technique for validating gene function^41^. To investigate the roles of NAE1, UBA3, and UBE2M in the URM1 modification pathway in human cells, we designed and transfected HEK293T and HeLa cells with small interfering RNAs (siRNAs) targeting these genes: siNAE1, siUBA3, and siUBE2M. To assess the efficiency of NAE1 knockdown and its impact on overall protein urmylation, we transfected HEK293T cells with siNAE1 and analyzed them 48 h post-transfection using Western blot. As shown in Fig. 4A, siNAE1 effectively reduced NAE1 expression, with only a weak signal detected at 60 kDa compared to a strong NAE1 band in control. A smaller band was also observed in the control, which, given its significantly reduced intensity in siNAE1-treated cells compared to non-treated cells, likely represents a degradation product detected by anti-NAE1. After confirming NAE1 silencing by siNAE1, we analyzed protein urmylation levels in these two samples. Western blot analysis using anti-URM1 revealed a clear presence of multiple urmylated proteins with molecular weights above 50 kDa in the non-treated control, whereas these protein complexes were markedly diminished in siNAE1-treated cells. NAE1 functions as the regulatory subunit for UBA3, which possesses catalytic activity within their formed complex. Given that NAE1 and UBA3 form a tightly bound complex, the reduction of NAE1 may lead to destabilization and potential degradation of UBA3 via the proteasome pathway. To confirm this, we performed Western blot analysis using anti-UBA3, which showed a significant reduction in UBA3 levels in siNAE1-transfected cells compared to its ready detection in non-transfected control cells. This decrease in UBA3 levels in siNAE1-transfected cells closely correlates with the observed reduction in protein urmylation in transfected cells. We conducted a similar NAE1 gene silencing experiment in HeLa cells. As shown in Fig. 4B, siNAE1 effectively knocked down NAE1 expression in HeLa cells and led to a significant decrease in overall urmylated proteins and a corresponding reduction in UBA3 levels. Similar downregulation of protein urmylation was observed with UBA3 gene silencing. As shown in Fig. 4C, siUBA3 efficiently knocked down UBA3 expression in HEK293T cells, leading to a reduction in overall protein urmylation levels compared to non-transfected control cells. Likewise, in HeLa cells, siUBA3 transfection decreased UBA3 expression and reduced overall urmylated protein levels, as shown in Fig. 4D. The consistent results in both HEK293T and HeLa cells strongly support the functional role of the NAE1/UBA3 complex as an E1 enzyme in the URM1 modification pathway in human cells.

UBE2M gene knockdown was performed using siUBE2M. HEK293T cells were transfected with siUBE2M, and 48 h later, UBE2M expression and overall protein urmylation levels were analyzed via Western blot analysis. As shown in Fig. 4E, siUBE2M effectively reduced UBE2M expression to a minimal level in transfected cells, compared to a strong UBE2M band observed in non-transfected control cells. Additionally, overall urmylated protein complexes detected by anti-URM1 were significantly reduced in siUBE2M-transfected cells. A similar UBE2M knockdown experiment was conducted in HeLa cells. As shown in Fig. 4F, siUBE2M efficiently silenced UBE2M expression, leading to a corresponding decrease in urmylated protein complexes detected by anti-URM1. Collectively, our results demonstrate that UBE2M functions as an E2 enzyme in the URM1 modification pathway in human cells.

### Validation of NAE1/UBA3 and UBE2M as E1 and E2 enzymes in the URM1 modification pathway in human cells through enzymatic inhibition

MLN4924 (Pevonedistat) is a small-molecule investigational drug that selectively inhibits the NAE1/UBA3 complex^42^. It binds to the enzyme’s active site, blocking its catalytic activity to activate NEDD8, a UBL protein as well. MLN4924 is known to induce DNA damage, oxidative stress, and cell cycle arrest in rapidly proliferating cancer cells, which was previously thought to occur through inhibition of the NEDD8 modification pathway^43^. Given its strong antitumor potency, MLN4924 has been evaluated in multiple clinical trials, with ongoing studies exploring its potential to enhance anticancer efficacy in combination with chemotherapy or immune checkpoint inhibitors. To assess whether MLN4924 also inhibits protein urmylation, we treated HEK293T and HeLa cells with increasing concentrations of MLN4924 for 24 h, followed by Western blot analysis to examine changes in protein urmylation. As shown in Fig. 4G and Supplementary Fig. 5, protein urmylation was significantly reduced in a dose-dependent manner in both HEK293T and HeLa cells, with a more pronounced effect observed in HEK293T cells. These results confirm that inhibition of NAE1/UBA3 leads to diminished protein urmylation in human cells. Micafungin, an antifungal agent, inhibits UBE2M and has been shown in biochemical and cellular studies to block neddylation, albeit requiring high doses due to its relatively weak binding affinity for UBE2M^44^. We utilized micafungin to investigate the connection between UBE2M and protein urmylation. Both HEK293T and HeLa cells were treated with increasing concentrations of micafungin for 24 h, followed by Western blot analysis using anti-URM1 to detect changes in protein urmylation levels. As shown in Fig. 4H, urmylated protein levels in both cell lines were significantly downregulated in a dose-dependent manner upon micafungin treatment (Supplementary Fig. 5G, H). These results support the conclusion that UBE2M activity is required for protein urmylation in human cells. Overall, results from two small molecule inhibitors support that NAE1/UBA3 and UBE2M function as the E1 and E2 enzymes, respectively, in the URM1 modification pathway in human cells.

A biochemical assay containing NAE1/UBA3, UBE2M, URM1, and ATP produced a higher-molecular-weight UBE2M-reactive species under non-reducing conditions that is consistent with a putative UBE2M ~ URM1 thioester intermediate; omission of ATP, NAE1/UBA3, or URM1 abolished this species (Supplementary Fig. 6B, C). This putative intermediate was eliminated by N-ethylmaleimide and disrupted by glutathione or reducing agents (DTT or β-mercaptoethanol), consistent with catalytic cysteine dependence and thiol sensitivity (Supplementary Fig. 6D–F). In an E1-only reaction, a URM1-containing species consistent with an E1 ~ URM1 thioester intermediate formed in an ATP- and active-site cysteine-dependent manner (Supplementary Fig. 6G, H). Both putative E2 ~ URM1 and E1 ~ URM1 intermediates exhibited time- and temperature-dependent formation (Supplementary Fig. 7). Finally, excess NEDD8-GG reduced formation of both intermediates in these in vitro reactions (Supplementary Fig. 8), suggesting that NEDD8 and URM1 can engage the same E1/E2 enzymes when both C-terminal diglycine substrates are present. We note that this in vitro competition reflects enzyme–substrate compatibility under defined conditions and does not necessarily imply direct competition between endogenous NEDD8 and URM1 in cells, where relative abundance, compartmentalization, and regulatory context can be fundamentally different. Together, these behaviors are consistent with E1/E2∼Ubl thioester charging intermediates rather than stable non-covalent complexes.

### Oxidative stress-induced urmylation and its regulation by NAE1/UBA3 and UBE2M-DCN1 inhibitors

Oxidative stress-induced urmylation of cellular proteins has been observed in both yeast and human cells^6^. In yeast, this process promotes the formation of phase-separated assemblies that likely protect functionally critical proteins from further damage^16^. In human cells, it is possible that oxidative stress activates a URM1 modification pathway distinct from the one involving NAE1/UBA3 and UBE2M. To investigate whether NAE1/UBA3 and UBE2M function as major E1 and E2 enzymes for protein urmylation under oxidative stress, we tested whether their inhibition by small molecules blocks protein urmylation under oxidative stress conditions. Because MLN4924 and the UBE2M-DCN1 interface inhibitors were developed to perturb neddylation, their effects on URM1 conjugation could, in principle, include indirect consequences of broadly altering Cullin-RING ligase (CRL) activity and cellular stress signaling. We therefore interpret inhibitor phenotypes as supportive and consider them alongside orthogonal genetic and biochemical evidence.

**Fig. 5:**
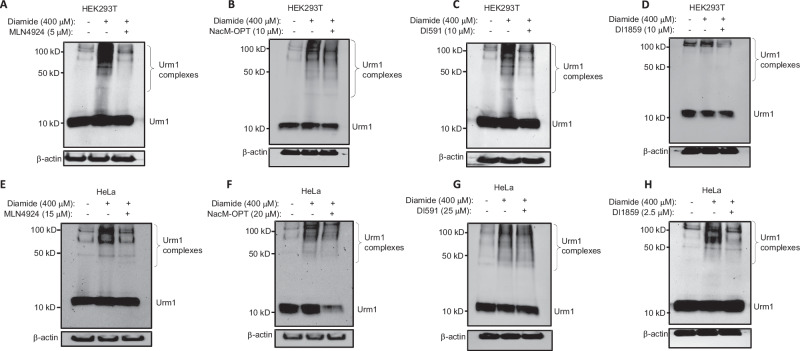
URM1 modification in response to oxidative stress and its inhibition by MLN4924, an inhibitor for NAE1/UBA3 and three inhibitors targeting the UBE2M-DCN1 interaction. **A**–**D** Inhibition of oxidative stress-induced protein urmylation in HEK293T cells by MLN4924, NacM-OPT, DI591, and DI1859. **E**–**H** Inhibition of oxidative stress induced-protein urmylation in HeLa cells by the same inhibitors. HEK293T and HeLa cells were treated with the indicated concentrations of inhibitors for 24 h before exposure to 400 μM diamide. Protein urmylation levels were analyzed 10 min and 45 min post-diamide treatment for HEK293T and HeLa cells, respectively. Samples in **A**–**H** has been resolved by native-PAGE. No. of independent experiments, in A-H, *n* = 3. Source data are provided as a Source Data file.

Diamide, a potent oxidative reagent that depletes cellular reducing agents such as glutathione and thioredoxin, is commonly used to disrupt redox homeostasis and promote reactive oxygen species (ROS) formation. We utilized diamide to trigger oxidative stress in human cells. Treating HEK293T cells with 400 µM diamide for 10 min significantly increased overall protein urmylation levels compared to untreated control cells (Fig. 5A). However, pretreatment with 5 µM MLN4924 for 24 h before a 10-min diamide exposure resulted in a marked reduction in protein urmylation levels, supporting the role of the NAE1/UBA3 complex as an E1 enzyme in URM1 modification under oxidative stress. Because micafungin requires high concentrations for effective UBE2M inhibition, we explored alternative inhibitors targeting UBE2M to assess its role in oxidative stress-induced urmylation. DCN1, a scaffold-type E3 ligase, interacts with UBE2M to mediate neddylation (NEDD8 modification) of cullins, which are ubiquitin ligases^45^. Given the importance of cullins in various human diseases, considerable efforts have been made to develop inhibitors that block the UBE2M-DCN1 interaction. Since UBE2M and DCN1 form a tightly bound complex, we suspected that DCN1 may function as an E3 enzyme in the URM1 modification pathway. To test this hypothesis, we employed three UBE2M-DCN1 inhibitors, NacM-OPT^46^, DI591^47^, and DI1859^48^, designed to bind DCN1 at its unique pocket that interacts with the acetylated *N*-terminus of UBE2M, thereby disrupting their association. Among the three inhibitors, DI1859 is the most potent by forming a covalent complex with DCN1 (Supplementary Fig. 9A–F). Since these inhibitors selectively target DCN1 rather than UBE2M, their effects can reveal whether DCN1 is the primary E3 ligase for URM1 modification. If UBE2M associates with other E3 enzymes, inhibition of DCN1 alone would not significantly alter urmylation levels. We first tested these inhibitors in HEK293T cells under oxidative stress. Cells were pretreated with inhibitors for 24 h before diamide exposure for 10 min. As shown in Fig. 5B–D, all three inhibitors effectively blocked diamide-induced protein urmylation, with DI1859 exhibiting the strongest effect due to its high potency. We further assessed the inhibitory effects of MLN4924 and the three UBE2M-DCN1 inhibitors in HeLa cells. As shown in Fig. 5E–G, higher concentrations of MLN4924, NacM-OPT, and DI591 were required to observe inhibition, likely due to the increased resistance of cancerous HeLa cells to small-molecule treatments (Supplementary Fig. 10B–E). Prolonged diamide treatment for 45 min was also used to observe activated urmylation in HeLa cells (Supplementary Fig. 10A). Notably, DI1859 exhibited substantial cytotoxicity in HeLa cells, causing significant cell death at 10 µM (Supplementary Fig. 10F). Therefore, we used a lower concentration (2.5 µM) that led to strong inhibition of oxidative stress-induced urmylation, as shown in Fig. 5H.

Collectively, our data provide strong evidence that NAE1/UBA3 and UBE2M are E1 and E2 enzymes for URM1 modification under oxidative stress and UBE2M engages DCN1 as an E3-like scaffold to promote URM1 transfer under oxidative stress. These findings support the existence of an NAE1/UBA3-UBE2M activation cascade and implicate DCN1 as an E3-like cofactor in the cellular URM1 conjugation pathway.

### Disruption of the URM1 pathway induces oxidative stress responses and sensitizes tumor cells to apoptosis

**Fig. 6:**
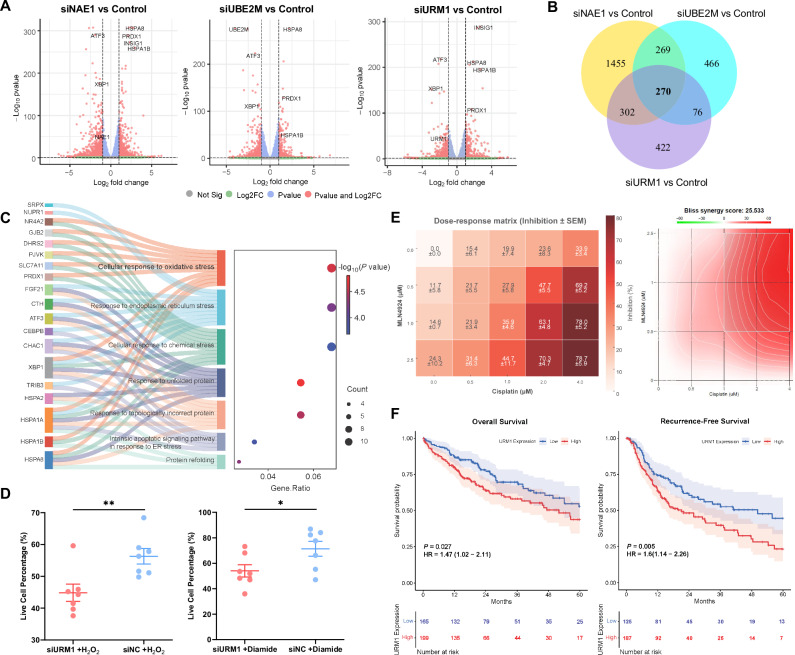
URM1 knockdown and MLN4924 treatment enhance oxidative stress and apoptosis in association with impaired URM1 pathway activity. **A** Volcano plots of differentially expressed genes (DEGs) upon siRNA-mediated knockdown of NAE1, UBE2M, and URM1 in HEK293T cells. Differential expression was analyzed using DESeq2. Statistical significance was assessed using two-sided Wald tests, and *P*-values were adjusted for multiple comparisons using the Benjamini–Hochberg method. Genes with adjusted *P* < 0.05 and |log2 fold change| > 1 were considered significant. **B** Venn diagram shows 270 shared DEGs among siNAE1, siUBE2M, and siURM1 vs Control groups. **C** Gene Ontology (GO) enrichment analysis of the shared DEGs highlights pathways related to oxidative stress, endoplasmic reticulum (ER) stress, and protein refolding. GO enrichment was performed using clusterProfiler; statistical significance was assessed by over-representation analysis (hypergeometric test) with Benjamini–Hochberg correction for multiple comparisons. **D** Percentage of live HepG2 cells after URM1 knockdown under oxidative stress. HepG2 cells were transfected with URM1-targeting siRNA (siURM1) or a non-targeting control siRNA (siNC) for 48 h and then treated with 200 μM H₂O₂ or 400 μM diamide for 16 h. Live cells were defined as Annexin V⁻/PI⁻ by flow cytometry. Each dot represents one independently transfected and treated cell sample; *n* = 7 biological replicates per group. Data are presented as mean ± SEM. Statistical significance was assessed using two-sided unpaired Student’s *t* tests comparing siURM1 + H₂O₂ versus siNC + H₂O₂, and siURM1 + diamide versus siNC + diamide. Exact *P*-values: H₂O₂, *P* = 0.0084; diamide, *P* = 0.0411. **E** Combination treatment with MLN4924 and cisplatin exhibits synergistic cytotoxicity. Heatmap displays dose-dependent inhibition and Bliss synergy scores. **F** Kaplan–Meier survival analysis stratified by URM1 expression in patient cohorts. Blue and red curves denote the low- and high-expression groups, respectively. Shaded bands represent the 95% confidence intervals around the Kaplan–Meier survival estimates. *P*-values were calculated using the log-rank test. Representative sequential FACS gating strategy is shown in Supplementary Fig. 13. Source data are provided as a Source Data file.

To understand gene expression patterns regulated by URM1 and its two controlling enzymes NAE1/UBA3 and UBE2M, transcriptomic profiling was performed on HEK293T cells treated with three siRNAs targeting NAE1, UBE2M, and URM1 genes. Quality assessment and clustering analyses confirmed high reproducibility across biological replicates (Supplementary Table 4, Supplementary Fig. 11A, B). Differential expression analysis identified a substantial number of differentially expressed genes (DEGs) under each knockdown condition. Volcano plots illustrated distinct yet partially overlapping gene expression changes among the three groups (Fig. 6A). Notably, several oxidative stress–related genes were consistently dysregulated across all three conditions, including upregulation of PRDX1^49^, HSPA8, and HSPA1B^50^, and downregulation of XBP1 and ATF3^51^. These genes are known to play key roles in redox homeostasis and cellular adaptation to oxidative insults, suggesting a critical function of the URM1 pathway in maintaining redox homeostasis. This trend was further supported by enrichment results comparing URM1 knockdown to control samples (Supplementary Fig. 11C, D, Supplementary Table 5).

To visualize global transcriptional changes, a hierarchical clustering heatmap was generated based on all DEGs across the three knockdown groups (Supplementary Fig. 11E), revealing both shared and distinct expression patterns. To define a core set of commonly regulated genes, Venn diagram analysis identified 270 significantly altered genes shared across all three knockdown conditions, representing a conserved transcriptional signature associated with URM1 pathway disruption (Fig. 6B). Gene Ontology (GO) enrichment analysis of this shared gene set revealed strong enrichment of stress-related biological processes, including cellular responses to oxidative stress, endoplasmic reticulum (ER) stress, the unfolded protein response, and protein refolding (Fig. 6C). Among these, oxidative and ER stress responses were particularly prominent, suggesting a shared regulatory mechanism through which NAE1, UBE2M, and URM1 maintain cellular redox homeostasis and stress resilience. Given that NAE1 and UBE2M act as upstream enzymes in the urmylation cascade, it is reasonable to conclude that these stress responses are mediated, at least in part, through urmylation.

To functionally validate the role of URM1 in protection against oxidative stress, we extended our analysis to HepG2 cells, a human HCC line widely used to study redox biology due to the liver’s central role in detoxification and oxidative stress responses^52^. Following URM1 knockdown, cells were challenged with hydrogen peroxide (H₂O₂) or diamide, two agents that induce oxidative stress through distinct mechanisms. Knockdown of URM1 led to significantly reduced viability of HepG2 cells under both treatments compared with controls, indicating heightened sensitivity to oxidative damage (Fig. 6D). These findings support a cytoprotective function of URM1 against oxidative stress and provide functional support for its predicted role in redox regulation.

Inspired by these findings, we hypothesized that disruption of the URM1 pathway may sensitize tumor cells to agents that induce oxidative stress, such as cisplatin. Cisplatin is a widely used chemotherapeutic drug known to generate reactive oxygen species (ROS) and induce oxidative DNA damage, contributing to its cytotoxic effects^53^. We next evaluated whether pharmacological inhibition of the URM1 pathway could further sensitize cells to oxidative stress–induced cytotoxicity. Combined treatment with MLN4924, a NAE1/UBA3 inhibitor that functionally impairs the URM1 pathway, and cisplatin demonstrated a synergistic decrease in cell viability. This synergism was quantitatively assessed using dose–response matrices and synergy models (Bliss, HSA, and Loewe), yielding synergy scores exceeding 20 (Fig. 6E, Supplementary Fig. 12). These results strongly indicate that chemical disruption of the URM1 axis may potentiate chemotherapy effectiveness under oxidative stress conditions. Collectively, these findings support a model in which the URM1 pathway serves as a critical determinant of cellular resistance to oxidative stress. Genetic depletion or pharmacological inhibition of this pathway increases tumor cell vulnerability to oxidative damage, thereby enhancing apoptosis.

Finally, to investigate the clinical relevance of URM1 in liver cancer, we analyzed patient survival outcomes in HCC cohorts stratified by URM1 expression. Patients with high URM1 expression exhibited significantly poorer overall survival (HR = 1.47, 95% CI: 1.02–2.11, *P* = 0.027) and recurrence-free survival (HR = 1.61, 95% CI: 1.14–2.26, *P* = 0.005) compared with those with low expression (Fig. 6F). These findings suggest that URM1 may promote tumor progression by enhancing cellular adaptation to oxidative stress and highlight its potential as a clinically relevant biomarker in HCC.

## Discussion

**Fig. 7:**
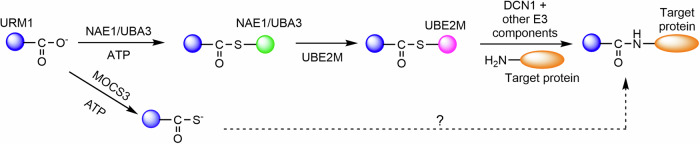
Working model of URM1 modification in human cells via the NAE1/UBA3–UBE2M–DCN1 axis. A possible alternative route via a URM1 thiocarboxylate intermediate remains to be validated.

The NAE1/UBA3-UBE2M-DCN1 catalytic cascade is a known E1-E2-E3 pathway that regulates protein neddylation, a posttranslational UBL modification essential for cellular homeostasis^54^. Neddylation of substrate proteins, primarily CRLs are key regulators of protein degradation via the ubiquitin-proteasome system. Modifications of CRLs enhance their activities in controlling cell cycle progression, DNA repair, and signal transduction^55^. Dysregulation of this pathway has been implicated in various cancers and other diseases^56^. As a result, the NAE1/UBA3-UBE2M-DCN1 axis has emerged as an attractive target for drug discovery, with inhibitors such as MLN4924 disrupting NAE1/UBA3 function to block CRL activation, leading to cell cycle arrest and apoptosis in cancer cells. Additionally, multiple small molecule inhibitors targeting the UBE2M-DCN1 interaction have been developed to selectively impair neddylation for potential cancer intervention. Our findings expand the functional scope of this enzymatic axis by demonstrating that it also catalyzes posttranslational protein urmylation in human cells (Fig. 7), thereby linking neddylation and urmylation within a shared regulatory framework. This conclusion is supported by integrated transcriptomic, proteomic, functional, and clinical data indicating that the URM1 pathway plays a conserved role in cellular stress adaptation. Given the abundance of cellular NEDD8, we do not infer global competition between URM1 and NEDD8 in vivo under basal conditions. Substrate or channel selection between neddylation and urmylation may depend on stress-regulated availability of activated URM1 species, adapter/E3 usage, and compartmentalization, variables that will require dedicated mechanistic dissection. Still, the functional convergence of neddylation and urmylation on a common catalytic machinery creates potential synergism or complication for therapeutic targeting. Any inhibitors developed for neddylation may also inhibit URM1 modification as well. Our results clearly show that URM1 serves a protective role against oxidative stress in human cells. An inhibitor for URM1 modification will compromise this potential protective function, leading to not only synergistic tumor killing effects for oxidative stress-inducing agents such as cisplatin, but also potential toxic side effects to normal tissues and cells. Although the functional experiments presented here are necessarily initial, they support a cytoprotective role of URM1 under oxidative stress and motivate future substrate-mapping and in vivo genetic studies to define physiological contexts where this pathway is most critical.

Despite progress, many questions remain regarding URM1 modification in human cells. Compared to ubiquitination and other UBL modifications, URM1 modification is still poorly understood. However, multiple proteins undergo urmylation under both normal and oxidative stress conditions and identifying these protein targets will be key to elucidating the biological functions of URM1 modification and the impact of NAE1/UBA3-UBE2M-DCN1 inhibitors on cellular processes. Notably, URM1 expression is negatively correlated with survival in liver cancer patients, suggesting a potential role in oxidative-stress adaptation. As a metabolically active organ constantly exposed to oxidative stress, the liver may rely on URM1 modification to protect against oxidative damage. Understanding the precise functions of URM1 and its modification in liver diseases will require further investigation. In addition, given the robust induction of URM1 conjugation by oxidative stress, an important open question is whether stress-induced urmylation is universally routed through the NAE1/UBA3-UBE2M cascade or whether additional mechanisms contribute under specific contexts. Dissecting the relative contributions of these mechanisms under defined stress or developmental conditions will require future mechanistic studies.

Overall, our results identify NAE1/UBA3 and UBE2M as an E1/E2 activation cascade for URM1 and implicate DCN1 as an E3-like cofactor/scaffold that promotes URM1 conjugation under oxidative stress in human cells. On the grounds that multiple small-molecule inhibitors have already been developed for this axis, our findings provide a straightforward framework for characterizing protein urmylation and its functional roles in human biology. This work lays the foundation for future studies on the interplay between urmylation, oxidative stress responses, neddylation, and disease progression.

## Methods

### Ethics statement

All animal procedures were approved by the Texas A&M University Institutional Animal Care and Use Committee (IACUC) under protocol IACUC 2025-0071 and were conducted in accordance with relevant guidelines and regulations.

### Chemicals

Propargylamine was purchased from TCI Chemicals, USA. MLN4924 (Catalog No. B1036) and Micafungin sodium (Catalog No. A3606) were purchased from APExBIO, USA. NAcM-OPT (Cat. No.: HY-111505), DI-591 (Cat. No.: HY-124602), and DI-1859 (Cat. No.: HY-145733) were acquired from MedChemExpress, USA. Diamide (Cat. No. D3648) and H₂O₂ (Cat. No. H1009) were purchased from Millipore Sigma, USA. All commercial small-molecule reagents and inhibitors were purchased from the vendors indicated and used as received in accordance with the manufacturers’ specifications.

### Preparation of FLAG-URM1-G101Pa probe

#### Expression and purification of FLAG-URM1-G101C-6×His

To generate the precursor for probe synthesis, an overnight culture of *E. coli* BL21(DE3) cells harboring a Flag-URM1-G101C-6×His expression vector (34) was inoculated into LB media containing 100 μg/mL ampicillin. All plasmid constructs were verified by Sanger sequencing prior to use. Cells were grown at 37 °C until OD_600_ reached 0.6–0.7, followed by the addition of 1 mM isopropyl-β-thiogalactoside (IPTG) to induce protein expression. Induced cells were grown in an incubating shaker at 18 °C overnight and harvested by centrifugation (4000 rpm, 20 min, 4 °C). To purify Flag-URM1-G101C-6×His, the bacterial pellet was resuspended in a Ni^2+^ binding buffer (50 mM NaH_2_PO_4_, 500 mM NaCl, 5 mM imidazole, 1 mM TCEP, pH 7.8) supplied with 0.2 mg/mL lysozyme (Sigma-Aldrich) and lysed by sonication on ice. The total cell lysate was clarified by centrifugation at 10,000 rpm for 30 min at 4 °C. Resulting supernatant was applied to a high-affinity Ni²⁺-NTA resin chromatography (Genescript, USA). Prior to use, Ni²⁺-NTA resins were equilibrated with buffer containing 50 mM NaH_2_PO_4_, 150 mM NaCl, 0.5 mM MgCl₂, 20 mM imidazole, and 1 mM TCEP at pH 7. The resins were subsequently washed with 100 mL of Ni^2+^-NTA wash buffer A (50 mM NaH_2_PO_4_, 900 mM NaCl, 0.5 mM MgCl₂, 20 mM imidazole, 0.1% Triton X-100, 1 mM TCEP, pH 7), followed by 100 mL of Ni^2+^-NTA washing buffer B (50 mM NaH_2_PO_4_, 150 mM NaCl, 0.5 mM MgCl₂, 20 mM imidazole, 1 mM TCEP, pH 7). Finally, the bound protein was eluted into 5 mL of elution buffer containing 50 mM NaH_2_PO_4_, 150 mM NaCl, 250 mM imidazole, and 1 mM TCEP at pH 7. Eluted Flag-URM1-G101C-6×His was desalted into 50 mM ammonium bicarbonate (ABC) buffer using a HiPrep 26/10 Desalting column (Cytiva, USA) in an ӒKTA pure™ chromatography system (AKTA pure, Cytiva, USA) and analyzed by electrospray ionization MS (ESI-MS) and 15% SDS-PAGE. The concentration of protein solution was measured by Pierce™ 660 nm Protein Assay Reagent (Thermo Fisher Scientific, USA). The purified protein was then dispensed into 100 nmol aliquots for lyophilization. Eventually, protein pellets were either used for chemical reactions or kept at −80 °C for long-term storage.

### The synthesis of FLAG-URM1-G101Pa via ACPL

To react with FLAG-URM1-G101C-6×His that was purified to homogeneity, 500 mM propargylamine was dissolved in water. 500 mM TCEP stock and 5 mM 2-Nitro-5-thiocyanatobenzoic acid (NTCB, Sigma Millipore, Cat. No. 30211-77-9) stocks were prepared in water and DMSO, respectively. To conduct ACPL reactions, FLAG-URM1-G101C-6×His pellet was dissolved in the propargylamine stock solution and 0.5 mM TCEP and 5 mM NTCB were sequentially introduced into the reaction mixture. pH of the reaction mixture was adjusted to 9 and incubated at 37 °C for 16 h. This was followed by desalting using a HiTrap Desalting column. Then, Ni^2+^-NTA resins were incubated with desalted products at room temperature for 30 min to remove unreacted original protein and intermediates. After that, micro syringe filters were used to remove resins, and flowthrough was collected for ESI-MS analysis.

### Cell culture

HEK293T and HeLa cells were obtained from the American Type Culture Collection (ATCC), and HepG2 cells were obtained from the Margie Moczygemba laboratory (Texas A&M University) and were originally sourced from ATCC. ATCC authenticates human cell lines by STR profiling. All cell lines were cultured in Dulbecco’s Modified Eagle Medium (DMEM; Gibco) supplemented with 10% fetal bovine serum (FBS; Gibco) and 1% penicillin-streptomycin (Gibco) at 37 °C in a humidified incubator with 5% CO₂.

### Cell and tissue lysate preparation

#### HEK293T and HeLa cell lysates

HEK293T and HeLa cells were cultured in T75 flasks until they reached approximately 90% confluency. Once ready, the cells were harvested in a 1× PBS buffer and transferred into eppendorf tubes. To induce cell lysis, 500 μl Pierce™ IP Lysis Buffer (ThermoFisher, USA) was added to the cell suspension, which was then gently shaken under refrigeration for 30 min to ensure complete lysis. Following this lysis process, cell lysates were centrifuged at 14,000 rpm for 30 min at 4 °C. Resulting supernatant was collected, aliquoted, rapidly frozen in liquid nitrogen, and stored at −80 °C.

#### Mouse tissue lysates

Male C57BL/6J mice (3 months old) were maintained at the Texas A&M University animal facility under a 12 h light/12 h dark cycle, at an ambient temperature of 20–24 °C and relative humidity of 40–60%. Mouse tissues (lung, liver, kidney, and brain) were freshly dissected and immediately transferred into pre-chilled eppendorf tubes placed on dry ice. Frozen tissue samples were weighed, and 20–50 mg of tissue was taken for preparing lysates. Extra tissue was stored at −80 °C for long-term preservation. Following this, lysis buffer (1× RIPA, 50 mM Tris-HCl, 150 mM NaCl, 1% Triton X-100, 0.5% sodium deoxycholate, 0.1% SDS,1 mM EDTA, 1 mM EGTA, pH 7.4) was prepared and kept on ice, and 20 μL/mg of RIPA was added to the tissue (400–1000 μL total). Tissues were homogenized using tissue homogenizer and centrifuged at 12,000 rpm at 4 °C for 15 min. The resulting supernatant was collected and transferred into a clean tube. The concentration of each tissue lysate was determined by Pierce™ BCA Protein Assay Kit (ThermoFisher, Cat. No. 23227).

### Immunoprecipitation assays

Pierce™ Anti-DYKDDDDK Magnetic Agarose beads (Thermo Fisher, Cat. No. A36797) was used to enrich proteins captured by FLAG-URM1-G101Pa. Cell lysates were incubated overnight with 6 μM FLAG-URM1-G101Pa. Prior to use, the beads were washed thrice with binding buffer (Pierce™ IP Lysis Buffer containing 25 mM Tris-HCl pH 7.4, 150 mM NaCl, 1 mM EDTA, 1% NP-40, and 5% glycerol). Cell lysates incubated with Flag-URM1-G101Pa were added to pre-washed magnetic agarose and incubated for 2 h at room temperature with gentle shaking. The beads were collected using a magnetic stand, and the supernatant was saved as the flow-through fraction. The beads were washed thrice with wash buffer containing 1× PBS and 0.025% Tween™-20 and once with autoclaved deionized water. Anti-FLAG-enriched protein complexes were eluted using Pierce IgG Elution Buffer (acidic pH 2.8) and the pH was neutralized by adding 1 M Tris HCl at pH 8.

### Immunoblotting

FLAG-enriched proteins were separated by SDS-PAGE. Following electrophoresis, proteins were transferred from the gel to a 0.2 µm nitrocellulose membrane using the iBlot™ Transfer Stack (ThermoFisher, Cat. No. IB23001) according to manufacturer’s protocol. The membrane was then blocked with 1X Tris-buffered saline containing 0.1% Tween-20 (TBS-T) and 5% non-fat dry milk powder at room temperature for 1 h. Primary antibodies were diluted in 1X TBS-T and applied to the membrane, followed by overnight incubation with shaking at 4 °C. The membranes were subsequently washed four times with TBS-T for 10 min each. After washing, the membranes were incubated with horseradish peroxidase-conjugated secondary antibodies for 1 h at room temperature. Signal detection was performed using an enhanced chemiluminescence detection kit (Pierce™ ECL Western Blotting Substrate, Cat. No.32106). The primary and secondary antibodies used are listed in Supplementary Table 3.

### Proteomic sample preparation and protein identification by LC-MS/MS analysis

#### Sample preparation

Protein complexes eluted from HEK293T and HeLa cell lysates using FLAG-URM1-G101Pa (FLAG-URM1-G101C-6×His as a control) were dried by a Speed-vac, reconstituted in 30 μl of 2× Laemmli sample buffer (Bio-Rad), and heated at 95 °C for 10 min. The resulting proteins were subsequently loaded onto a 15% SDS-PAGE gel, and the gel was run at 90 V for 30 min. After staining with Coomassie blue and destaining with a solution of methanol, acetic acid and water (50:10:40, v/v), the gel band was excised into four sections, and each was cut into 1 mm^3^ cubes. The gel pieces underwent further destaining and dehydration using a mixture of 50 mM ammounium bicarbonate and acetonitrite (75:25 and 50:50, v/v) twice. To the gel pieces were added 10 mM dithiothreitol and 55 mM iodoacetamide for cysteine reduction and alkylation at 37 °C for 1 h and at room temperature in the dark for 30 min, respectively. The proteins were subsequently digested in-gel with trypsin (Thermo Fisher Scientific, Cat. No. 90058) at 37 °C overnight and the resultant peptides were dried and desalted with OMIX C18 tips, following the manufacturer’s recommended procedures (Thermo Fisher Scientific, Cat. No.87784).

#### MS data analysis

The desalted peptides were dissolved in 0.1% formic acid solution and subjected to LC-MS/MS analysis on an Orbitrap Fusion Lumos Tribrid mass spectrometer coupled with an EASY-nLCTM 1000 system and a high-field asymmetric-waveform ion mobility spectrometry (FAIMS) (Thermo Fisher Scientific). The compensation voltages for FAIMS were −40, −60 and −80 V. Following the gel separation procedure described above, four samples from the experimental and control groups in one biological replicate from HEK293T and HeLa cells resulted in a total of 16 samples. All 16 samples were loaded onto a trapping column (Inner diameter 150 μm, outer diameter 360 μm) packed in house with 5 μm C18 resin (Dr. Maisch, r15.aq) with 0.1% formic acid, and peptides were subsequently eluted onto a 20-cm analytical column (Inner diameter 75 μm, outer diameter 360 μm) packed with 3 μm C18 resins (Dr. Maisch, r13.aq), where the flow rate was 300 nl/min. The mass spectrometer was operated in a data-dependent acquisition mode with a 1-s cycle time, during which the top 20 most abundant ions detected in full scan were chosen for fragmentation by higher-energy collisional activation, and the resulting MS/MS were acquired in the linear Ion trap with rapid scan speed. The raw LC-MS/MS data were converted into mzXML format using FAIMS_MzXML_Generator (Version: 1.1.8003.32506) followed by searching with MaxQuant (2.1.2.0) against the Uniprot database (UP000005640_9606). Cysteine carbamidomethylation was a fixed modification, and methionine oxidation and N-terminal acetylation were variable modifications. A mass tolerance of 20 ppm was set for both MS and MS/MS. A maximum of two trypsin missed cleavages were permitted, and the peptides were filtered at 1% false discovery rate (FDR). Label-free quantification was employed with a minimum ratio count of 2. The match between runs option was implemented with a match time window of 0.7 min. Identified proteins were first filtered by deleting the contaminants and reverse hits in proteinGroups.txt file generated by MaxQuant. Subsequently, prefiltered proteins detected in the experimental samples but absent or significantly reduced in the control samples were considered as candidate interacting proteins.

### Biochemical binding assay

1 μM FLAG-URM1-G101Pa and 1 μM UBE2M (UBPBio, Cat. No. C3100) or UBA3 (Prospec, Cat. No. ENZ-576) were incubated in HEPES-NaCl buffer (20 mM HEPES, 150 mM NaCl, pH 7.4) at 37 °C for 1 h. The resulting protein mixture was then used for immunoblotting, as shown in Fig. 3E, F. Recombinant UBE2M and UBA3 were purchased as purified proteins from the indicated vendors and used according to the manufacturers’ specifications.

### Genetic knockdown using siRNA

HEK293T, HeLa, and HepG2 cells were seeded in complete DMEM (10% FBS), prepared without antibiotics, and incubated for 24 h to reach 70–80% confluency. Cells were transfected using Lipofectamine™ RNAiMAX Transfection Reagent (Thermo Fisher Scientific, Cat. No. 13778075) with one of the following siRNAs: URM1 (final concentration: 60 nM; MCE, Cat. No. HY-RS15508), NAE1 (50 nM; MCE, Cat. No. HY-RS09007), or UBE2M (70 nM; MCE, Cat. No. HY-RS15355). Sequences are listed in Supplementary Table 4. A FAM-labeled siRNA (MCE) was used as a positive control to monitor transfection efficiency, and a non-targeting siRNA was included as a negative control. Cells were incubated with the transfection mixture for 48 h. Total protein or RNA was harvested for subsequent analyses. Knockdown efficiency was validated by immunoblotting (Fig. 4).

### Oxidative stress and chemical inhibitor treatment

Three T-75 flasks of HEK293T and HeLa cells were cultured in complete DMEM (10% FBS) for 24 h, reaching 70–80% confluency. The cultures were then treated with respective inhibitors, as specified in the main text, and incubated for 24 h. Two other flasks were treated with the same volume of DMSO used for dissolving inhibitors and served as controls. After 24 h, the culture medium was replaced with fresh medium, and both inhibitor-treated cells and untreated controls were subsequently exposed to oxidative stress by treatment with 400 μM diamide, 15 min for HEK293T cells and 45 min for HeLa cells. One flask was kept unstressed and untreated with inhibitors, serving as the “untreated” control. Following oxidative stress exposure (15 or 45 min), cells were collected for lysate preparation. Cell lysate volumes were consistently adjusted to correspond to a cell count of 10^6^ cells.

### RNA sequencing and data analysis

RNA extraction, library preparation, sequencing were performed by Novogene. *Sample preparation and sequencing.* Total RNA was extracted from HEK293T cells transfected with siRNAs targeting NAE1, UBE2M, or URM1, as described above, and harvested 48 h post-transfection. RNA integrity was assessed using the Agilent 2100 Bioanalyzer (Agilent Technologies). Messenger RNA was purified from total RNA using poly-T oligo-attached magnetic beads, fragmented, and used for first-strand cDNA synthesis with random hexamer primers. Second-strand cDNA was synthesized with dUTP to retain strand specificity. After end repair, A-tailing, adapter ligation, size selection, amplification, and purification, libraries were quality-checked by Qubit fluorometry, real-time PCR, and Bioanalyzer analysis. Libraries were sequenced on an Illumina platform to generate paired-end 150 bp reads. *Data processing and analysis.* Raw sequencing reads were trimmed and filtered using fastp (v0.20.0), and clean reads were aligned to the human reference genome (GRCh38) with HISAT2 v2.0.5. Gene expression was quantified using featureCounts v1.5.0-p3, and fragments per kilobase of transcript per million mapped reads (FPKM) were calculated. Differential expression analysis was conducted with the DESeq2 R package (v1.20.0), applying an adjusted *P*-value < 0.05 and |log₂ fold change| ≥ 1. Gene Ontology (GO) and KEGG pathway enrichment analyses were performed with the clusterProfiler R package (v3.8.1). Gene Set Enrichment Analysis (GSEA) was conducted using the Broad Institute’s GSEA software (v4.2.3) against curated GO and KEGG datasets.

### Apoptosis assay

HepG2 cells were transfected with siRNA targeting URM1 as described above. After 48 h, cells were treated with 200 μM hydrogen peroxide (H₂O₂) or 400 μM diamide for 16 h to induce oxidative stress. Cells were then harvested, washed with PBS, and stained with APC-conjugated Annexin V and propidium iodide (PI) using the Annexin V Apoptosis Detection Kit with PI (BioLegend), following the manufacturer’s instructions. Stained cells were analyzed immediately on a CytoFLEX flow cytometer (Beckman Coulter), and data were processed using FlowJo software. Live cells were defined as Annexin V⁻/PI⁻ and gated based on unstained and single-stained controls. Heat-killed cells were included as a positive control for apoptosis induction. A minimum of 20,000 events was collected per sample, and seven biological replicates (*n* = 7) were analyzed for each condition.

### Assessment of synergistic drug effects

#### Drug treatment and cell viability assay

HepG2 cells were seeded in 96-well plates and allowed to adhere overnight. Cells were first treated with MLN4924 (2.5, 1, or 0.5 μM) or DMSO (vehicle control) for 24 h, followed by the addition of cisplatin at indicated concentrations (4, 2, 1, or 0.5 μM) or DMF (vehicle control). After 48 h of combined treatment, the culture medium was replaced with fresh medium before adding Cell Counting Kit-8 (CCK-8; MCE) reagent. Briefly, CCK-8 reagent was added to each well, and cells were incubated for 2 h at 37 °C before absorbance was measured at 450 nm using a Synergy H1 microplate reader (BioTek Instruments). Each condition was tested in triplicate wells, and the entire experiment was independently repeated three times (*n* = 3 biological replicates). Cell viability was normalized to vehicle-treated controls, and the percentage of inhibition was calculated as (1 – normalized viability) × 100%.

#### Synergy analysis

Synergy between MLN4924 and cisplatin was evaluated using Bliss independence, Loewe additivity, and highest single agent (HSA) models. Synergy scores and synergy heatmaps were generated using the SynergyFinder R package (v3.16.0). A synergy score >10 was considered synergistic, a score between –10 and 10 was interpreted as additive, and a score <–10 was considered antagonistic.

### Survival analysis

Survival analysis of URM1 expression in HCC patients was performed using data from The Cancer Genome Atlas (TCGA). Patients were stratified into URM1-high and URM1-low groups based on the optimal cutoff value determined by survival analysis. Kaplan–Meier survival curves for overall survival (OS) and recurrence-free survival (RFS) were generated, and differences between groups were assessed using the log-rank test. Hazard ratios (HRs) and 95% confidence intervals (CIs) were calculated. A *P* < 0.05 was considered statistically significant.

### Reporting summary

Further information on research design is available in the Nature Portfolio Reporting Summary linked to this article.

## Supplementary information

Supplementary Information

Reporting Summary

Transparent Peer Review file

## Source data

Source data

## Data Availability

The RNA-seq data generated in this study have been deposited in the Gene Expression Omnibus (GEO) under accession code GSE305835. The mass spectrometry proteomics data generated in this study have been deposited in the ProteomeXchange Consortium via the PRIDE partner repository under accession code PXD067405. The TCGA data used in this study are available from the Genomic Data Commons Data Portal [https://portal.gdc.cancer.gov]. Source data are provided with this paper. All other data supporting the findings of this study are available within the paper and its Supplementary Information. Source data are provided with this paper.
