# Supplementary material for: NAE1/UBA3-UBE2M are E1 and E2 enzymes for the URM1 modification
Source: Nat Commun. 2026 Apr 29;17:5858. doi: 10.1038/s41467-026-72296-w (PMC13333801; doi:10.1038/s41467-026-72296-w)
Supplement: Supplementary file 1 — Supplementary Information [file 41467_2026_72296_MOESM1_ESM.pdf]

Supplementary Figures

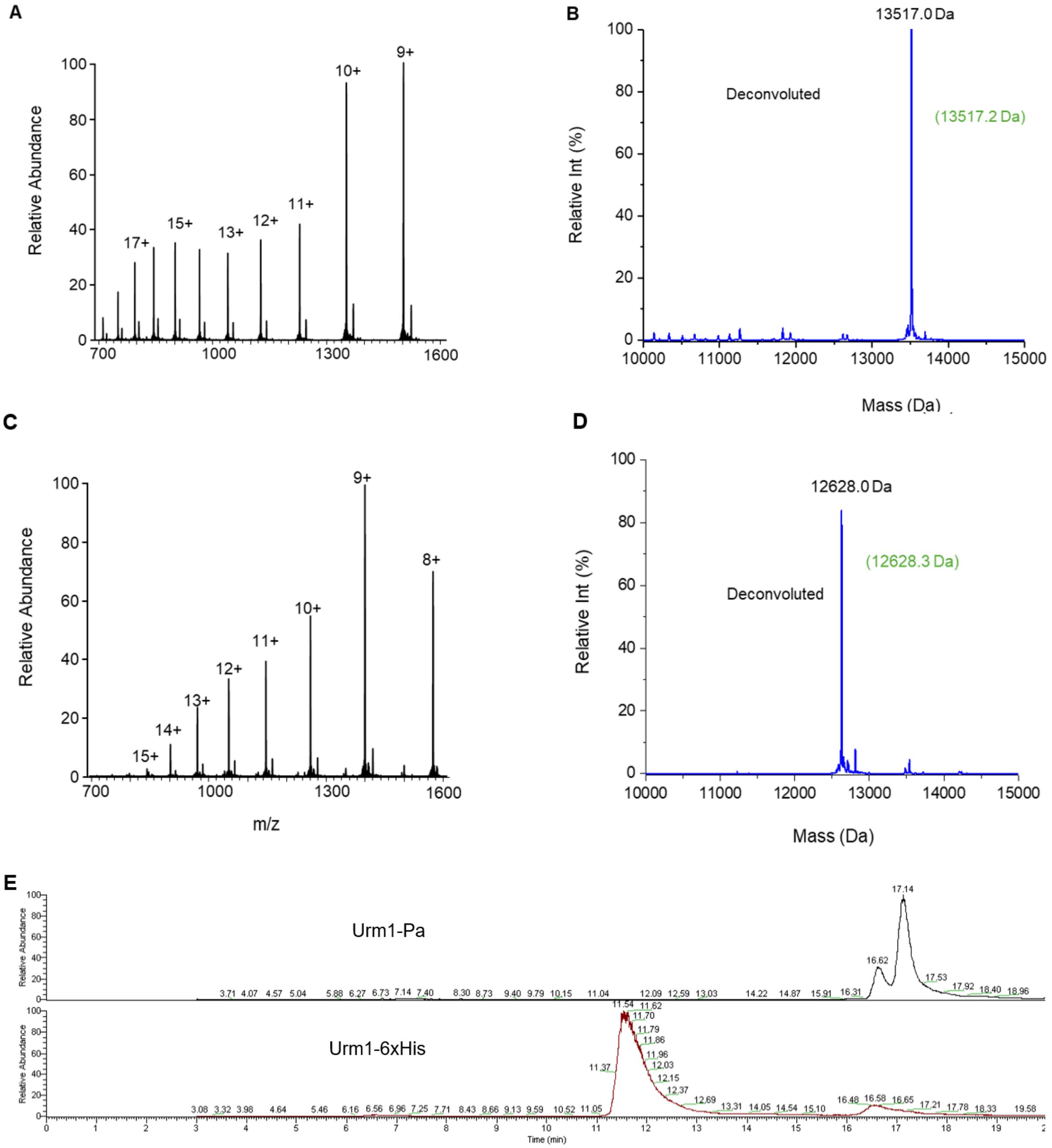

Supplementary Figure 1. ESI-MS of FLAG-URM1-G101C-6His and FLAG-URM1-G101Pa. (A) Positive-ion ESI-MS of FLAG-URM1-G101C-6×His. (B) Deconvoluted ESI-MS of FLAG-URM1-G101C-6×His. The theoretical average mass 13517.2 Da was calculated based on its elemental composition. (C) Positive-ion ESI-MS of FLAG-URM1-G101Pa. (D) Deconvoluted ESI-MS of FLAG-URM1-G101Pa. The theoretical average mass 12628.3 Da was calculated based on its elemental composition. (E) Analytical LC–MS chromatograms (TIC) of FLAG-URM1-G101C-6×His (starting material) and FLAG-URM1-G101Pa (product), showing distinct retention times and a predominant product peak, consistent with efficient conversion and high purity.

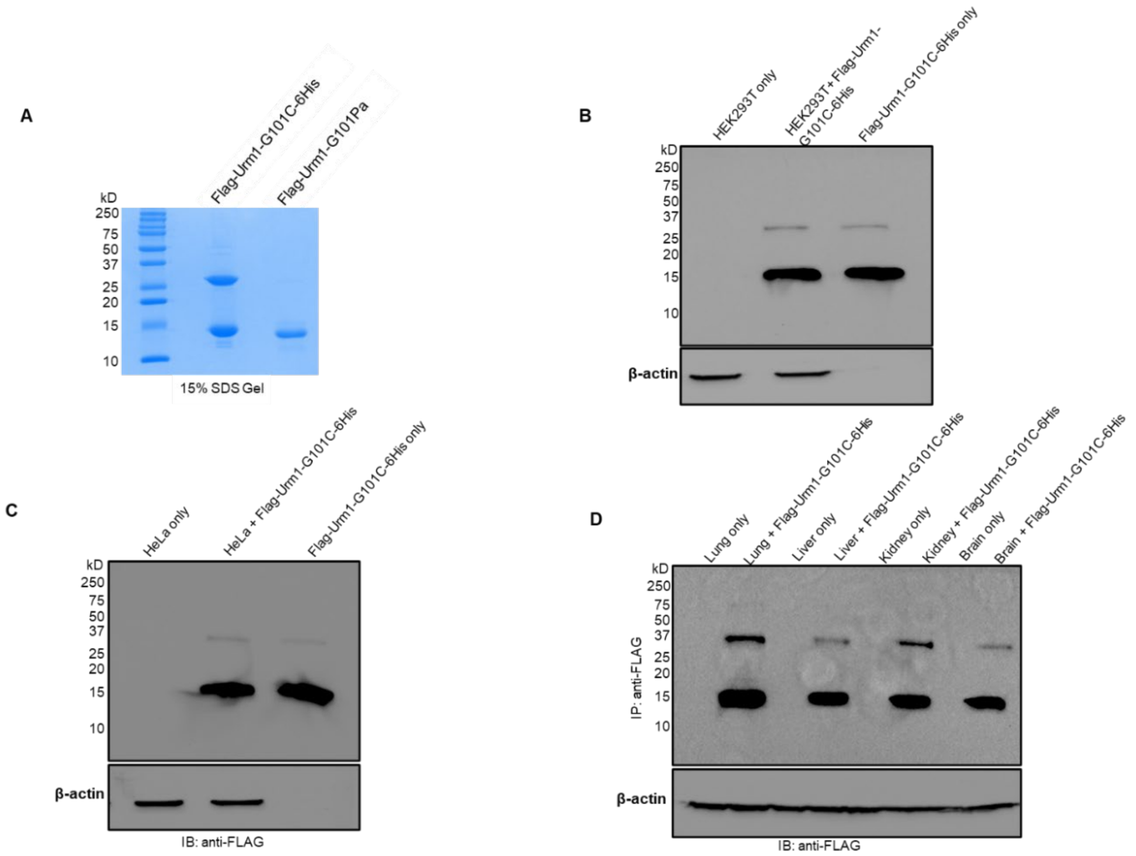

Supplementary Figure 2. Immunoblotting of FLAG-URM1-G101C-6×His binders from cell and tissue lysates. (A) SDS-PAGE analysis of purified FLAG-URM1-G101C-6×His and FLAG-URM1-G101Pa proteins.(B–D) Detection of cysteine-containing enzymes labeled by the negative control probe FLAG-URM1-G101C-6×His in: (B) HEK293T cells, (C) HeLa cells, and (D) homogenized tissues from four mouse organs. Anti-β-actin was used as a loading control on the same membranes. Shown are representative blots from n = 3 independent experiments with similar results. Full uncropped blots with molecular weight markers are provided in the Source Data file.

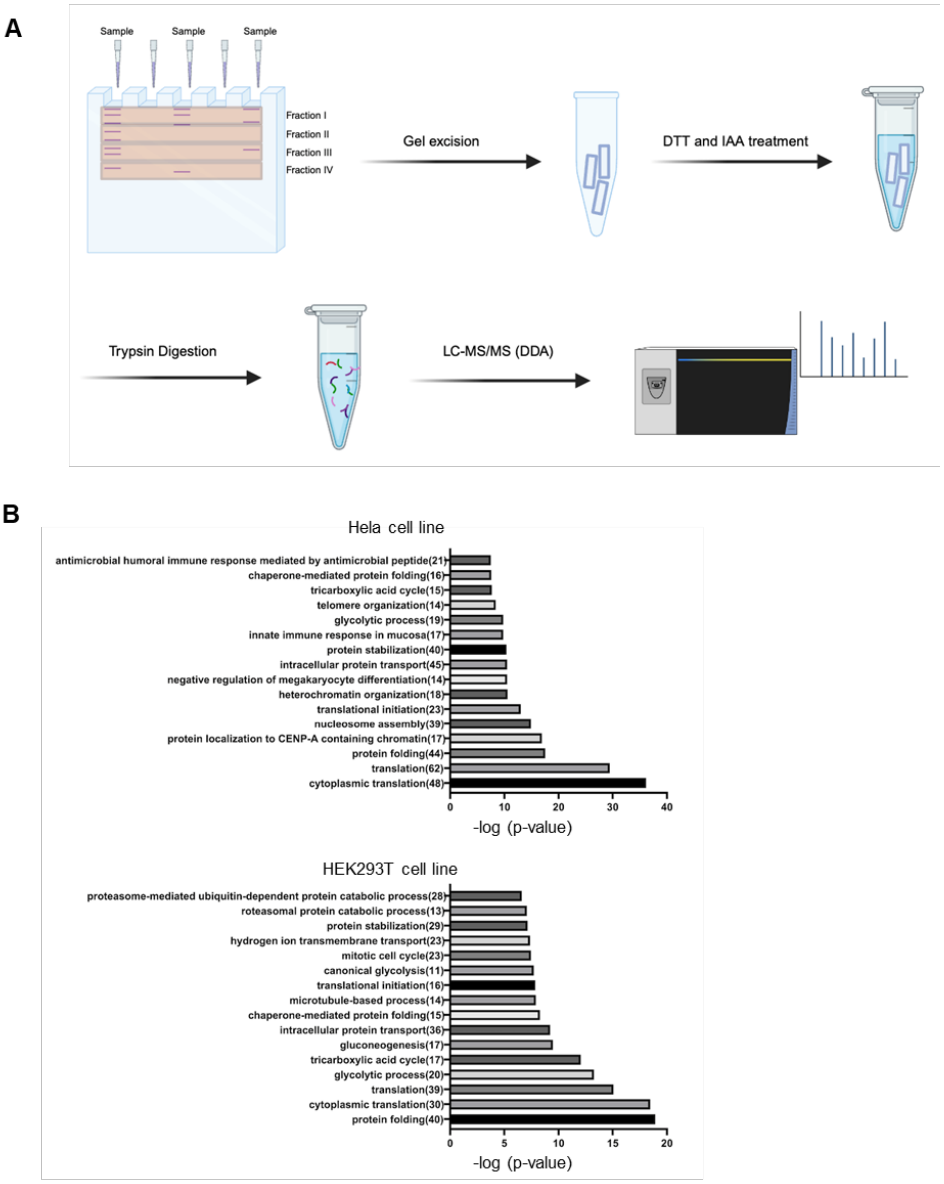

Supplementary Figure 3. LC-MS/MS for identifying URM1-interacting proteins. (A) In-gel digestion and LC-MS/MS analysis. ‘DTT’ and ‘IAA’ represent dithiothreitol and iodoacetamide, respectively. (B) Gene Ontology analysis of biological processes of the candidate URM1-interacting proteins. Created in BioRender. Wang, Y. (2026) https://BioRender.com/1nu6x5s

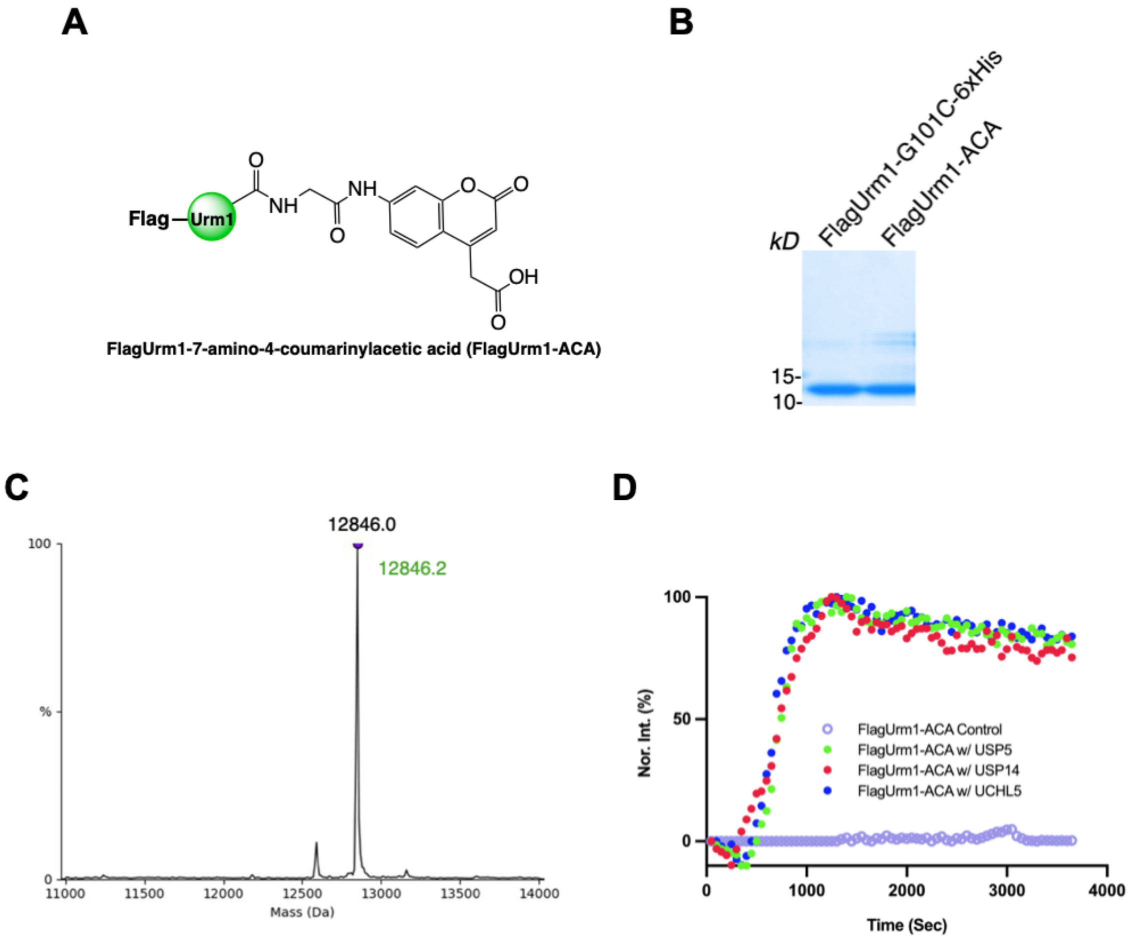

Supplementary Figure 4. Fluorometric assay of FLAG-URM1-ACA hydrolysis by USP5, USP14, and UCHL5. (A) The structure of FLAG-URM1-ACA. (B) SDS-PAGE analysis of recombinantly expressed FLAG-URM1-G101C-6×His and its ACPL reaction product FLAG-URM1-ACA by reacting with Gly-ACA. (C) Deconvoluted ESI-MS of FLAG-URM1-ACA. Its theoretical molecular weight is 12,846.2 Da. (D) USP5, USP14, and UCHL5-catalyzed ACA release from FLAG-URM1-ACA. Recombinant USP5, USP14, and UCHL5 (50 nM each) were incubated with 400 nM FLAG-URM1-ACA in assay buffer at 30 °C. Fluorescence intensity was recorded every 50 seconds for up to 1 hour (excitation: 380 nm; emission: 460 nm). Compared to control (probe only), all three enzymes triggered a marked increase in fluorescence, indicating cleavage of the FLAG-URM1-ACA probe and indicating hydrolysis of the URM1-ACA reporter under these in-vitro conditions.

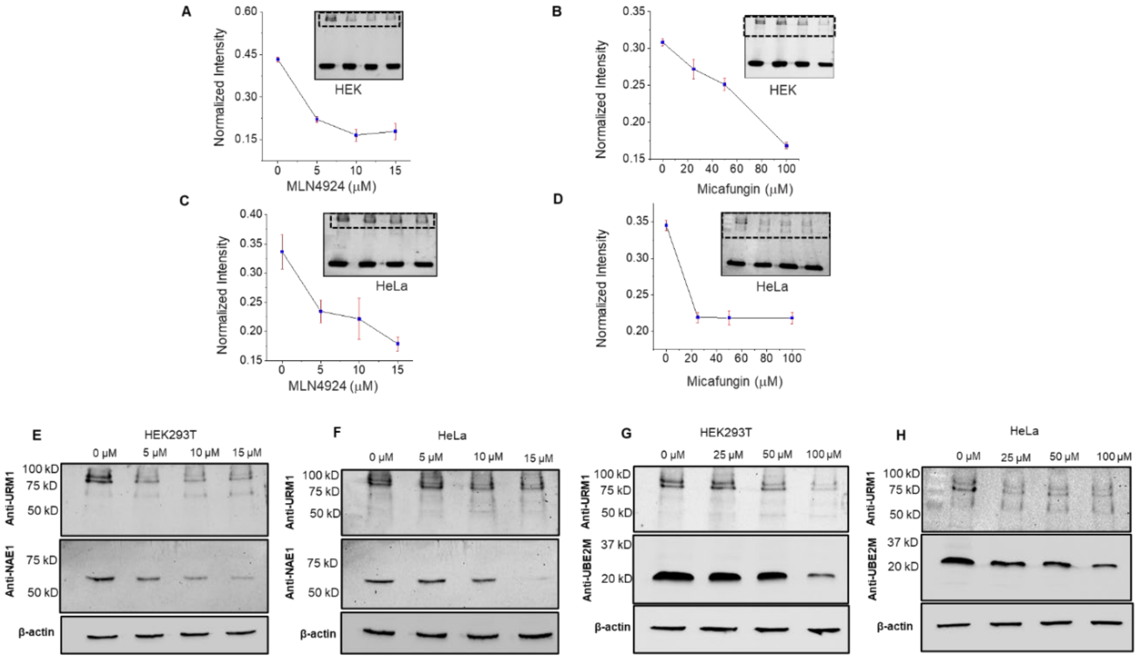

Supplementary Figure 5. Quantification of protein urmylation inhibition in HEK293T and HeLa cells by MLN4924 (a NAE inhibitor) and Micafungin (a UBE2M inhibitor). A decrease in normalized URM1 substrate intensity following treatment with MLN4924 or Micafungin reflects reduced urmylation in HEK293T (A, B) and HeLa (C, D) cells. A concentration-dependent reduction in NAE1 band intensity and protein urmylation is observed in HEK293T (E) and HeLa (F) cells upon MLN4924 treatment. In contrast, Micafungin treatment, which inhibits UBE2M, decreases protein urmylation in HEK293T (G) and HeLa (H) cells. Data in A–D are presented as mean ± SD from n = 3 independent experiments. Panels E–H show representative blots from n = 3 independent experiments with similar results. Full uncropped blots with molecular weight markers are provided in the Source Data file.

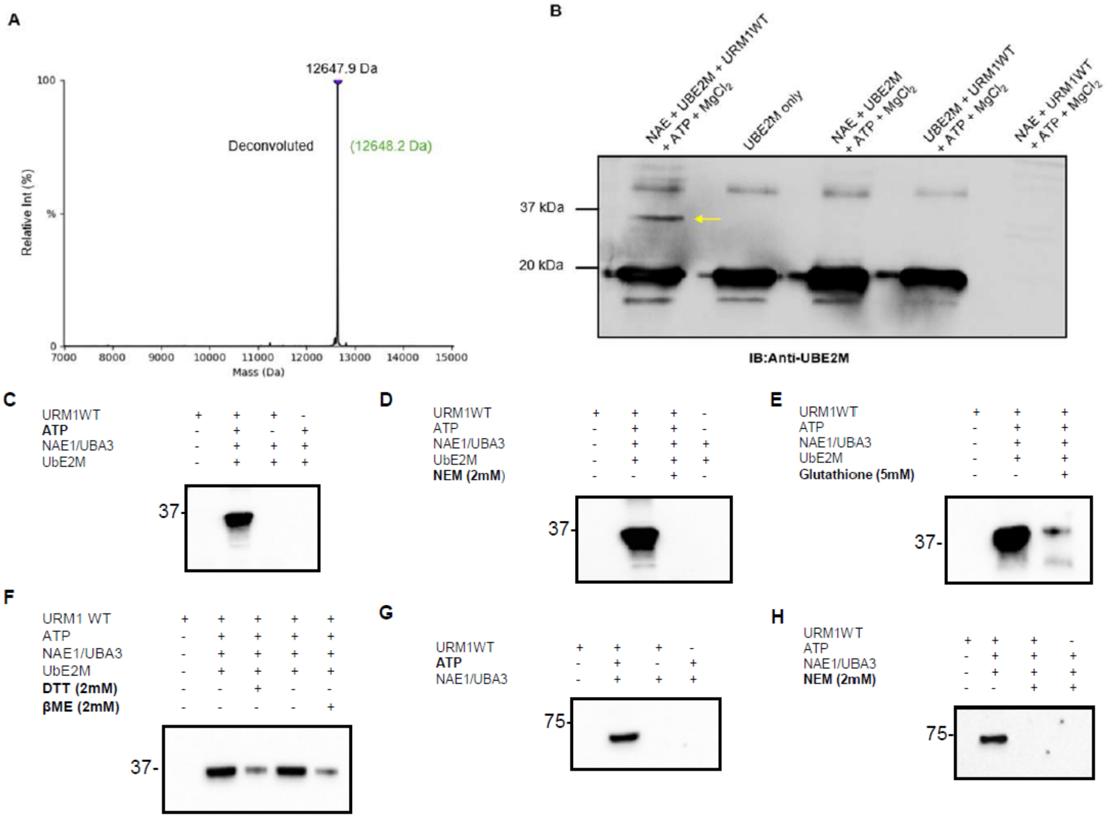

Supplementary Figure 6. In vitro reconstitution of URM1 charging and thioester-like intermediates. (A) Deconvoluted ESI-MS of wild-type FLAG-URM1-GG (URM1-WT). The theoretical molecular weight of 12648.2 Da (shown in green) was calculated based on its elemental composition. (B) UBE2M immunoblot detection of a putative UBE2M~URM1 intermediate. URM1-WT (15 µM) was incubated with NAE1/UBA3 (1 µM) and/or UBE2M (2 µM) in the presence of ATP (2 mM) and $MgCl_2$ (5 mM) in 20 mM HEPES, 150 mM NaCl, 5 mM TCEP (pH 7.5) at 37 °C for 45 min. Reactions were quenched in non-reducing SDS sample buffer resolved by SDS–PAGE, and immunoblotted with anti-UBE2M. A higher–molecular-weight UBE2M-reactive species (yellow arrow), consistent with a putative UBE2M~URM1 thioester-like intermediate, is detected only in the complete reaction and is not observed when essential components are omitted. (C–F) Anti-FLAG detection of URM1-containing intermediates and thioester diagnostics. Unless otherwise indicated, reactions contained FLAG–URM1WT (20 µM), NAE1/UBA3 (5 µM), and UBE2M (3 µM) with ATP (5 mM) and $MgCl_2$ (10 mM) in 50 mM HEPES, 150 mM NaCl, 5 mM TCEP (pH 7.5) and were incubated at 37 °C for 3 h, then quenched in non-reducing sample buffer and analyzed by anti-FLAG immunoblotting. (C) Component dependence of the putative UBE2M~URM1 species. (D) The putative UBE2M~URM1 species is abolished by N-ethylmaleimide (NEM; 2 mM; enzyme pre-treatment with removal of excess NEM), consistent with catalytic cysteine dependence. (E) The putative UBE2M~URM1 species is disrupted by glutathione (5 mM). (F) The putative UBE2M~URM1 species is reduced/disrupted upon post-reaction challenge with DTT (2 mM) or β-mercaptoethanol (βME; 2 mM) for 1 h at 37 °C prior to quenching, consistent with thiol sensitivity expected for thioester-linked intermediates. (G,H) E1~URM1 intermediate. Reactions were performed as in (C–F) but with UBE2M omitted, then analyzed by non-reducing SDS–PAGE and anti-FLAG immunoblotting. (G) Formation of an E1~URM1 species (~75 kDa) requires ATP. (H) The E1~URM1 species is abolished by NEM (2 mM; enzyme pre-treatment with removal of excess NEM), consistent with active-site cysteine dependence.

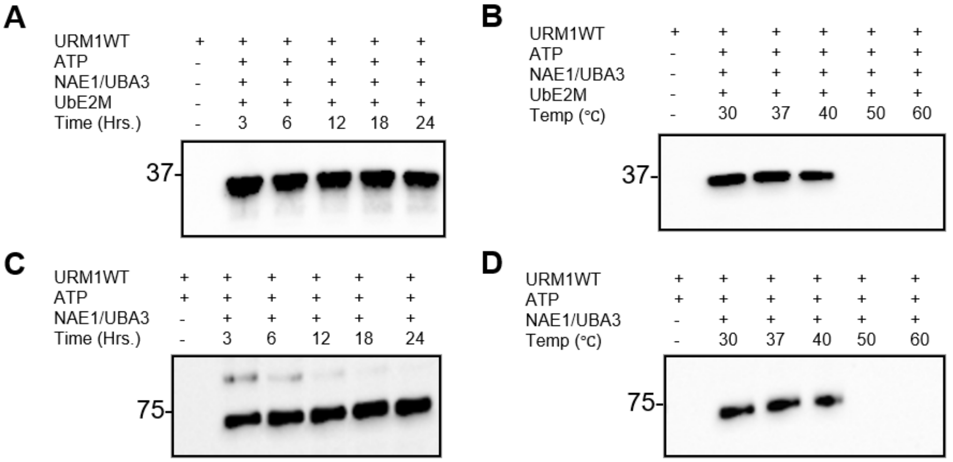

Supplementary Figure 7. Time- and temperature-dependence of URM1 charging intermediates formed by NAE1/UBA3 and UBE2M. (A) Time-course analysis of a URM1-containing species consistent with the putative UBE2M~URM1 charging intermediate formed in reactions containing FLAG–URM1-GG (URM1WT), ATP, $MgCl_2$, NAE1/UBA3, and UBE2M for the indicated times. (B) Temperature dependence of the putative UBE2M~URM1 intermediate formed at the indicated temperatures in the complete reaction. (C) Time-course analysis of a URM1-containing species consistent with the E1~URM1 thioester intermediate (~75 kDa) formed in an E1-only reaction containing URM1WT, ATP, $MgCl_2$, and NAE1/UBA3 for the indicated times. (D) Temperature dependence of the E1~URM1 intermediate formed at the indicated temperatures in the E1-only reaction. For all panels, reaction mixtures were resolved by SDS–PAGE and analyzed by immunoblotting with an anti-FLAG antibody.

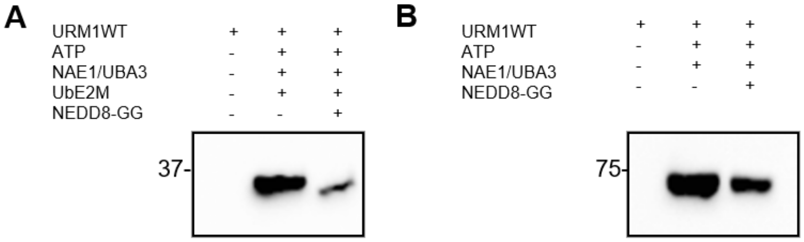

Supplementary Figure 8. NEDD8-GG competes with URM1-GG in NAE1/UBA3-dependent charging reactions. (A) E2 charging/transfer condition. Flag URM1-WT–GG (20 μM) was incubated with NAE1/UBA3 (5 μM) and UBE2M (3 μM) in the presence of ATP (5 mM) and $MgCl_2$ (10 mM) at 37 °C for 3 h. Addition of equimolar NEDD8-GG (20 μM; added at reaction initiation) reduces the abundance of the URM1-containing UBE2M~URM1 species. (B) E1 charging condition. Flag URM1-WT–GG (20 μM) was incubated with NAE1/UBA3 (5 μM) with ATP (5 mM) and $MgCl_2$ (10 mM) at 37 °C for 3 h (UBE2M omitted). Addition of equimolar NEDD8-GG (20 μM; added at reaction initiation) reduces formation of the URM1-containing UBA3~URM1 species. Reactions were quenched and analyzed by non-reducing SDS-PAGE and anti-FLAG immunoblotting.

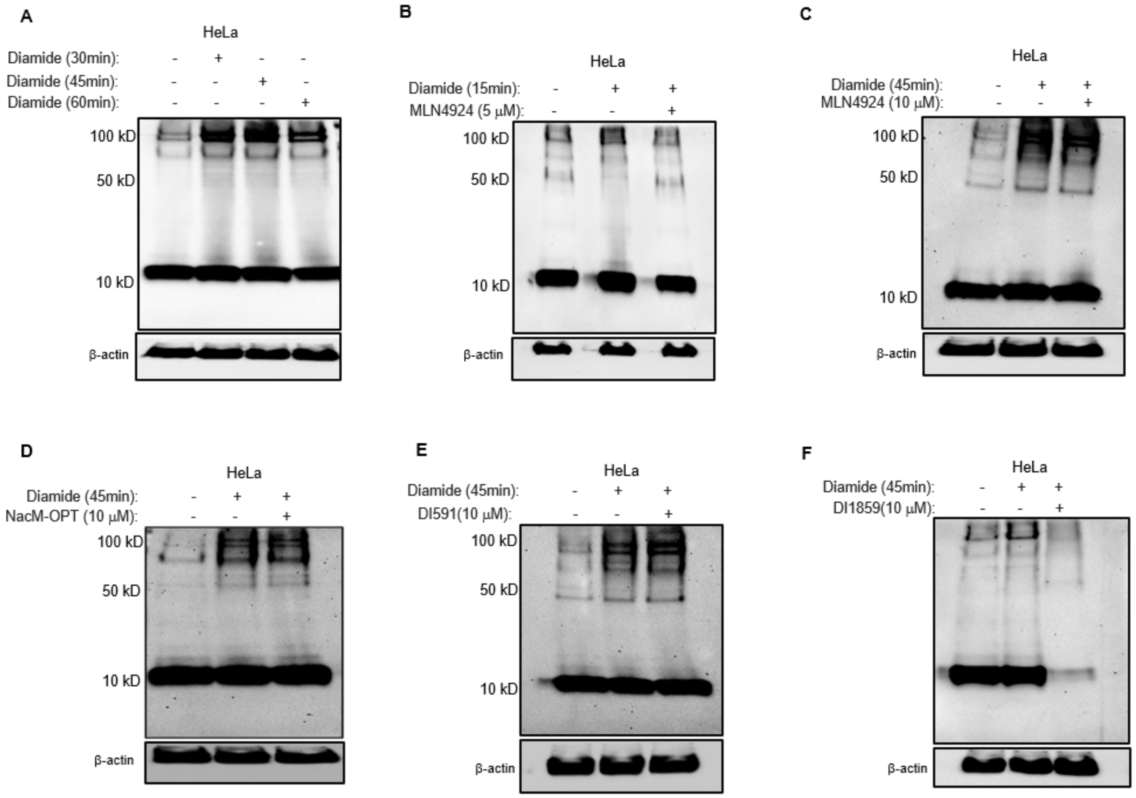

Supplementary Figure 9. Inhibition of URM1 conjugation under normal cellular conditions by UBE2M–DCN1 interaction inhibitors. (A–C) Suppression of protein urmylation in HEK293T cells treated with NacM-OPT, DI1859, or DI591. (D–F) Inhibition of protein urmylation in HeLa cells by the same compounds(n=3). Panels A–F show representative blots from n = 3 independent experiments with similar results. Samples in panels A–F were resolved by native-PAGE. Full uncropped blots with molecular weight markers are provided in the Source Data file.

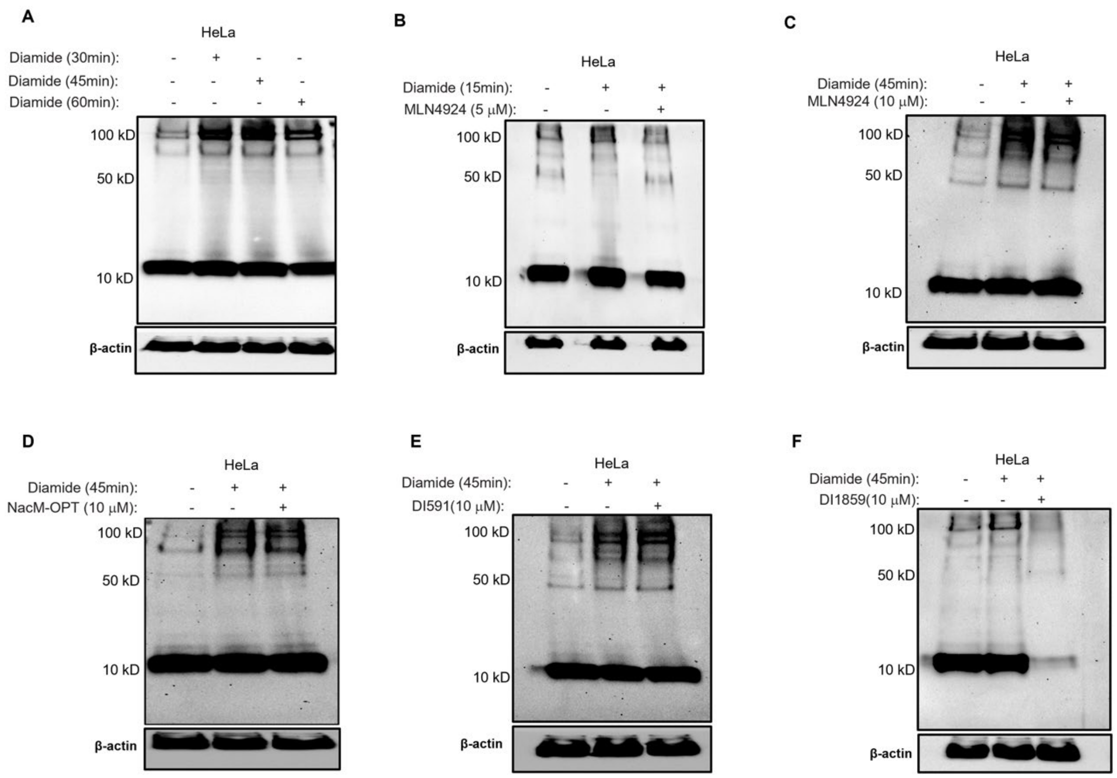

Supplementary Figure 10. Optimization of diamide treatment and inhibition of oxidative stress–induced urmylation in HeLa cells. (A) HeLa cells were treated with 400 μM diamide for 30, 45, or 60 minutes to optimize exposure time. (B) Initial inhibition of diamide-induced urmylation was assessed using 5 μM MLN4924 with a 15-minute diamide treatment. (C) Following optimization (45-minute exposure), inhibition was tested with 10 μM MLN4924. (D) Oxidative stress–induced urmylation was further evaluated using 10 μM NacM-OPT, DI591, and DI1859. Urmylation levels in (A–D) were analyzed by immunoblotting with an anti-URM1 antibody (n=3). Samples in panels A–D were resolved by native-PAGE. Full uncropped blots with molecular weight markers are provided in the Source Data file.

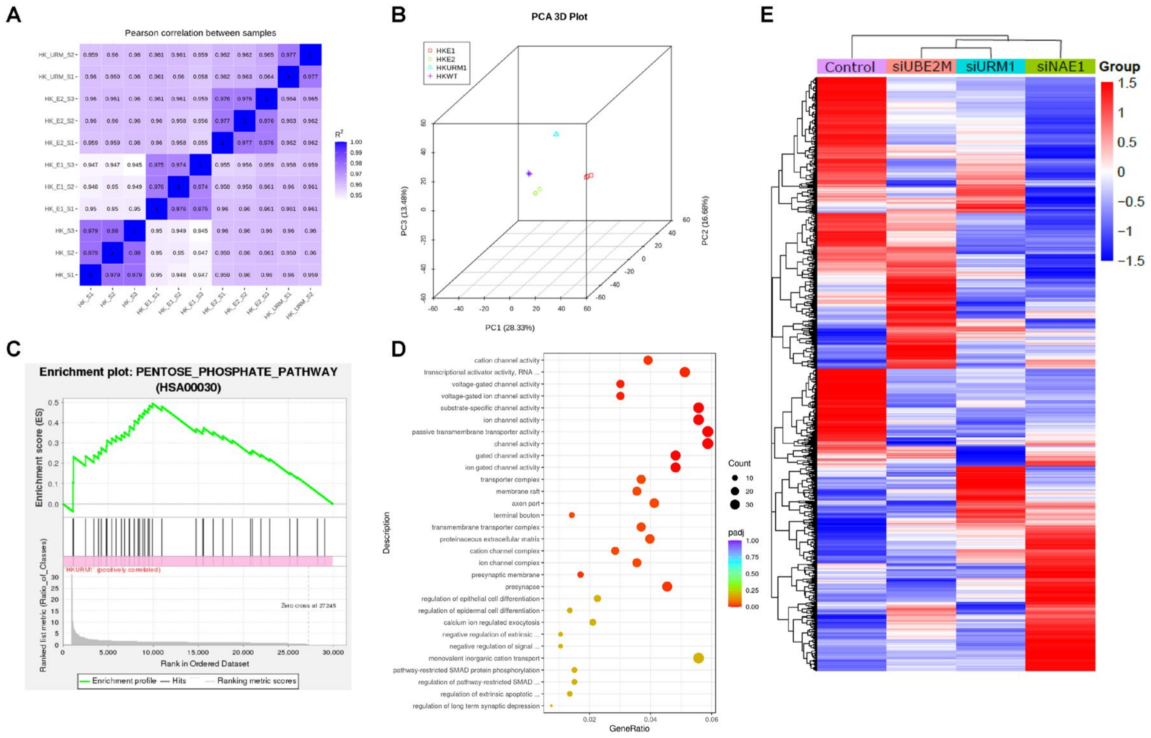

Supplementary Figure 11. Transcriptomic analyses reveal redox-related gene expression changes upon URM1 pathway perturbation. (A) Pearson correlation heatmap of RNA-seq samples across control (HKWT), NAE1 knockdown (HKE1), UBE2M knockdown (HKE2), and URM1 knockdown (HKURM1) conditions, showing high reproducibility within replicates. (B) Principal component analysis (PCA) of transcriptomic profiles reveals distinct clustering of treatment groups, with PC1 and PC2 capturing the majority of variance. (C) Gene set enrichment analysis (GSEA) of URM1-knockdown cells indicates significant upregulation of the pentose phosphate pathway (KEGG: hsa00030). (D) Gene Ontology (GO) enrichment analysis highlights terms related to ion transport, membrane function, apoptosis regulation, and SMAD signaling, consistent with a role for URM1 in redox homeostasis. (E) Heatmap of differentially expressed genes across control, siUBE2M, siURM1, and siNAE1 groups, with hierarchical clustering showing shared and distinct transcriptional responses. Gene expression values are row-scaled z-scores.

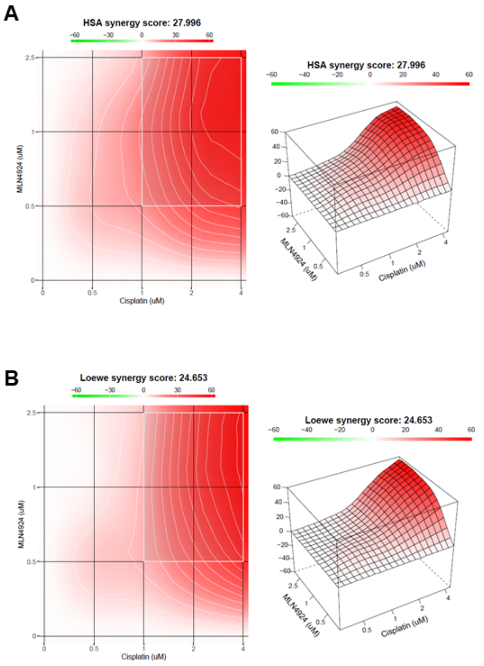

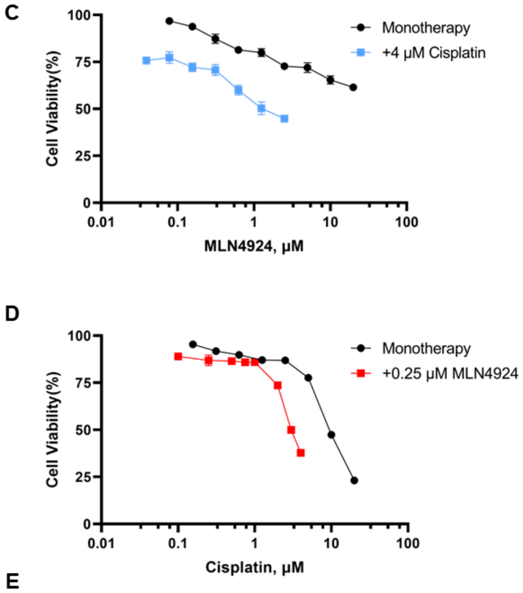

E

Treatment Condition	MLN4924 IC_{50} (μM, 95% CI)	Cisplatin IC_{50} (μM, 95% CI)
Monotherapy	17.33 (12.37–24.95)	5.66 (2.27–14.12)
+ 0.25 μM MLN4924	–	2.04 (1.11–3.77)
+ 4 μM Cisplatin	0.52 (0.27–1.03)	–

Supplementary Figure 12. MLN4924 and cisplatin exhibit synergistic cytotoxicity in combination treatments. (A) Drug interaction landscape assessed using the highest single agent (HSA) reference model. Heatmap and 3D surface plot represent synergy scores across a dose matrix of MLN4924 and cisplatin, with red indicating synergy and green indicating antagonism. The average HSA synergy score was 27.996. (B) Drug interaction analysis using the Loewe additivity model confirms synergy between MLN4924 and cisplatin, with an average synergy score of 24.653. (C) Dose–response curves of MLN4924 with or without 4 μM cisplatin. Combination treatment resulted in a leftward shift in the curve, indicating increased potency. (D) Dose–response curves of cisplatin with or without 0.25 μM MLN4924, showing a similar potentiation effect. (E) Summary table of IC_{50} values and 95% confidence intervals. Combination treatment reduced the apparent IC_{50} of both agents, consistent with a synergistic interaction. Data in C and D are presented as mean ± SEM from n = 3 biological replicates, with each condition tested in triplicate wells per experiment.

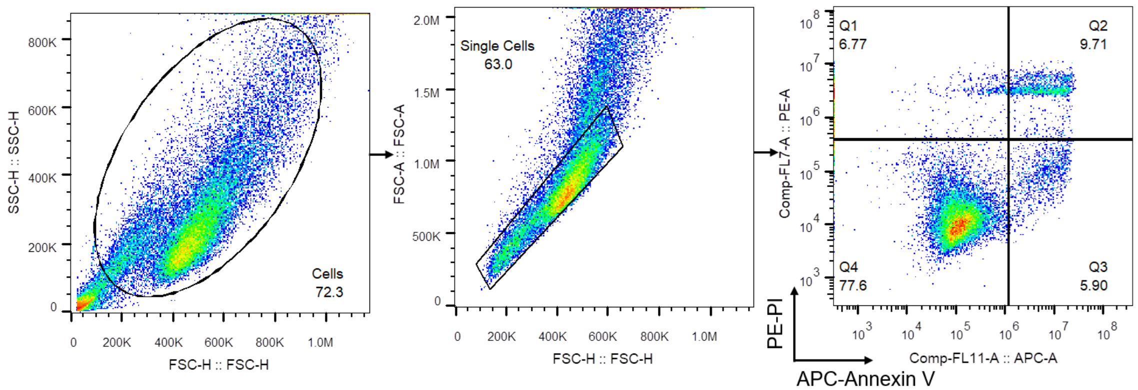

Supplementary Figure 13. Sequential FACS gating strategy for the apoptosis assay shown in Figure 6D. Representative sequential flow-cytometry gating strategy used for the apoptosis analysis in Figure 6D. Cells were first gated based on FSC/SSC properties to exclude debris, followed by singlet discrimination using FSC-A versus FSC-H. The final population was analyzed by Annexin V/PI staining. Live cells were defined as Annexin V$^-$/PI$^-$ (Q4), corresponding to the quantified population shown in Figure 6D. The same sequential gating strategy was applied to the H_2O_2 and diamide treated samples.

Supplementary Tables

Supplementary Table 1. List of E1, E2 and E3 enzymes identified by Flag-URM1-G101Pa in HEK293T and HeLa

Protein ID	Gene Symbol	Description
Q13564	NAE1*#	NEDD8-activating enzyme E1 regulatory subunit
Q8TBC4	UBA3#	NEDD8-activating enzyme E1 catalytic subunit
P61081	UBE2M*#	NEDD8-conjugating enzyme Ubc12
P62877	RBX1*	E3 ubiquitin-protein ligase RBX1, N-terminally processed

'' indicates present in HeLa. '#' indicates present in HEK293T. '*#' indicates present in both HEK293T and HeLa*

Supplementary Table 2. List of DUBs and other enzymes identified by Flag-URM1-G101Pa in HEK293T and HeLa

Protein ID	Gene Symbol	Description
P45974	USP5*#	Ubiquitin carboxyl-terminal hydrolase 5
P54578	USP14*#	Ubiquitin carboxyl-terminal hydrolase 14
Q96FW1	OTUB1*#	Ubiquitin thioesterase OTUB1
Q9Y5K5	UCHL5*#	Ubiquitin carboxyl-terminal hydrolase isozyme L5
Q06830	PRDX1*#	Peroxiredoxin-1
P32119	PRDX2*#	Peroxiredoxin-2
Q9NY33	DPP3*#	Dipeptidyl peptidase 3
O00231	PSMD11*#	26S proteasome non-ATPase regulatory subunit 11
O00232	PSMD12*#	26S proteasome non-ATPase regulatory subunit 12
P25786	PSMA1*#	Proteasome subunit alpha type-1
P25787	PSMA2*#	Proteasome subunit alpha type-2
P25788	PSMA3*#	Proteasome subunit alpha type-3
P25789	PSMA4*#	Proteasome subunit alpha type-4
Q5JRX3	PITRM1*#	Presequence protease, mitochondrial
Q16531	DDB1*#	DNA damage-binding protein 1
P13010	XRCC5*#	X-ray repair cross-complementing protein 5
P12956	XRCC6*#	X-ray repair cross-complementing protein 6
Q96T76	MMS19*#	MMS19 nucleotide excision repair protein homolog
P13639	EEF2*#	Elongation factor 2
P24539	ATP5F1*#	ATP synthase F (0) complex subunit B1, mitochondrial
P17066	HSPA6*#	Heat shock 70 kDa protein 6
O75608	LYPLA1*#	Acyl-protein thioesterase 1
P18887	XRCC1#	DNA repair protein XRCC1
P25705	ATP5A1*#	ATP synthase subunit alpha, mitochondrial

'' indicates present in HeLa. '#' indicates present in HEK293T. '*#' indicates present in both HEK293T and HeLa*

Supplementary Table 3. List of primary and secondary antibodies used in this study.

Antibody	Host / Type	Dilution	Vendor	Catalog No.
Anti-Flag	Mouse, monoclonal	1:2000	Millipore Sigma	A8592
Anti-URM1	Rabbit, polyclonal	1:1000	Proteintech	15285-1-AP
Anti-UBA3	Mouse, IgG2a	1:500	Thermo Fisher Scientific	MA536104
Anti-NAE1	Mouse, IgG	1:500	Thermo Fisher Scientific	H00008883-B01P
Anti-UBE2M	Mouse, IgG1	1:1000	Thermo Fisher Scientific	MA525739
Secondary for Anti-URM1	Goat anti-Rabbit IgG (H+L), HRP-conjugated	1:2000	Proteintech	SA00001-2
Secondary for Anti-UBA3	Goat anti-Mouse IgG2a, HRP-conjugated	1:2000	Thermo Fisher Scientific	M32207
Secondary for Anti-NAE1	Goat anti-Mouse IgG (H+L), HRP-conjugated	1:2000	ABClonal	AS003
Secondary for Anti-UBE2M	Goat anti-Mouse IgG1, HRP-conjugated	1:2000	Thermo Fisher Scientific	PA1-74421

Supplementary Table 4. Sequences of siRNAs designed for gene knockdown

Gene	Strand	Sequence (5'→3')	Catalog No.
**UBA3**	Sense	GCUAAAGAUAUUGGAAGAUU	HY-RS15321
	Antisense	UCUUCCAAUAUCUUUAGCUU	HY-RS15321
**NAE1**	Sense	GGAAGUUUGUGUUCAGUUUUU	HY-RS09007
	Antisense	AAACUGAACACAAACUUCCUU	HY-RS09007
**UBE2M**	Sense	CCAAAGAGGGUCAAGGAAAUU	HY-RS15355
	Antisense	UUUCCUUGACCCUCUUUGGUU	HY-RS15355
**URM1**	Sense	UCAUCUGGAUCAAGAAGAAUU	HY-RS15508
	Antisense	UUCUUCUUGAUCCAGAUGAUU	HY-RS15508
**Negative Control**		vendor-provided (IDT)	51-01-14-03

Supplementary Table 5. Gene set enrichment analysis (GSEA) results comparing URM1 knockdown (siURM1) versus control

Gene Set	Size	ES	NES	FDR q-value
Pentose Phosphate Pathway	38	0.49	1.89	0.094
Basal Cell Carcinoma	69	0.39	1.81	0.153
Melanoma	76	0.39	1.8	0.167
Glycine Serine And Threonine Metabolism	53	0.39	1.78	0.149
Breast Cancer	153	0.38	1.77	0.138
Mineral Absorption	69	0.5	1.73	0.136
Signaling Pathways Regulating Pluripotency Of Stem Cells	155	0.39	1.72	0.131
2 Oxocarboxylic Acid Metabolism	34	0.44	1.7	0.138
TGF-Beta Signaling Pathway	125	0.36	1.7	0.134
Transcriptional Misregulation In Cancer	211	0.37	1.69	0.131
Cell Adhesion Molecules	119	0.42	1.68	0.136
Central Carbon Metabolism In Cancer	93	0.42	1.68	0.133
Glycosphingolipid Biosynthesis Globo Series	19	0.54	1.66	0.144
Carbon Metabolism	177	0.41	1.61	0.147
Cysteine And Methionine Metabolism	68	0.36	1.61	0.142

Listed are gene sets enriched in the URM1 knockdown group, including pathway size, enrichment score (ES), normalized enrichment score (NES), and false discovery rate (FDR) q-value.

Supplementary Methods

Activity-Based Protein Profiling Using a Negative Control Probe. HEK293T and HeLa cell lysates, as well as mouse tissue lysates, were incubated overnight at 4°C with 6 μM FLAG-URM1-G101C-6His, a catalytically inactive control probe. FLAG-tagged proteins were enriched by co-immunoprecipitation using anti-FLAG magnetic agarose beads. Eluted proteins were separated by 15% SDS–PAGE and analyzed by immunoblotting with an anti-FLAG antibody (Supplementary Figure 2).

Synthesis of FlagURM1-ACA using ACPL. 500mM of GlyACA (2-(7-(2-aminoacetamido)-2-oxo-2H-chromen-4-yl) acetic acid was dissolved into 1xPBS buffer, and pH was slowly adjusted to 9.5 using 6M NaOH. At this stage, protein pellet of FlagURM1-G101C-6xHis was dissolved in the solution, followed by sequential addition of TCEP and NTCB. The reaction mixtures was incubated at 37 ºC, desalted to high-purity using a HiTrap column, and then incubated with Ni2+-NTA resins. The flow-through was removed with micro syringe filters and collected for ESI-MS analysis.

Monitoring catalytic activity of DUBs using FlagURM1-ACA. In a 96-well plate, 50nM of USP5, USP14 and UCHL5 were pre-incubated at 30 °C in an assay buffer containing 50mM Tris, 100mM NaCl, 0.5mM EDTA, 0.1% BSA (pH-7.6). After addition of 400nM of FlagURM1-ACA probe, fluorescence measurements were taken for 1 hour at every 50-second interval (excitation at 380 nm and emission at 460 nm) (Supplementary Figure 4).

Densitometric Analysis of Western Blot Bands. Western blot signals (Supplementary Figure 5) were quantified using Image Lab software by measuring the integrated intensity of each band following background subtraction. Normalized intensities were plotted using OriginPro 2017.

In Vitro Reconstitution of UBE2M–URM1 Conjugation. *Expression and Purification of FLAG-URM1-Wild-Type (URM1WT).* To express FLAG-URM1-Wild-Type (URM1WT), a construct was generated by restoring the glycine residue at position 101 and removing the C-terminal 6×His tag from the previously reported FLAG-URM1-G101C-6×His sequence. The synthetic gene was ordered from Twist Bioscience and cloned into an IPTG-inducible expression vector. The resulting plasmid was transformed into E. coli BL21(DE3) electrocompetent cells via electroporation and plated on LB agar supplemented with 50 μg/mL kanamycin. A single colony was grown overnight and expanded into 1 L LB medium containing the same antibiotic. Cultures were incubated at 37 °C with shaking until the OD_{600} reached 0.6–0.7, at which point protein expression was induced by adding 1 mM IPTG. Induced cultures were incubated overnight at 18 °C with shaking and then harvested by centrifugation. The bacterial pellet was resuspended in lysis buffer (50 mM NaH_2PO_4, 500 mM NaCl, 5 mM imidazole, 1 mM TCEP, pH 7.8), and lysed by sonication on ice. The lysate was clarified by centrifugation at 10,000 rpm for 30 min at 4 °C. The soluble fraction was then desalted using a HiPrep Desalting Column (GE Healthcare) into 50 mM ammonium bicarbonate. Desalted protein was lyophilized and stored at –80 °C.
In Vitro Reconstitution Assay of URM1WT. In vitro conjugation assay was performed by incubating 15 μM Flag-URM1WT with 1 μM of E1 enzyme NAE (NAE1/UBA3 complex) and 2 μM E2 (UBE2M) enzyme in the presence of 2 mM ATP and 5 mM $MgCl_2$. Reactions were carried out in a buffer containing 20 mM HEPES, 150 mM NaCl, and 5 mM TCEP at pH 7.5, and incubated at 37 °C for 45 minutes. To preserve thioester-linked charging intermediates, reactions were quenched with non-reducing SDS sample buffer and samples were not boiled prior to electrophoresis, then resolved by SDS–PAGE, and transferred onto a 0.2 μm nitrocellulose membrane.

Thioester characterization of URM1 charging intermediates. Putative thioester-like intermediates between URM1 and the E1 (NAE1/UBA3 complex) and/or E2 (UBE2M) enzymes were generated using purified recombinant proteins under conditions matching the in vitro reconstitution assays. For UBE2M immunoblot detection (Supplementary Figure 6B), URM1-WT (15 μM) was incubated with NAE1/UBA3 (1 μM) and/or UBE2M (2 μM) in the presence of ATP (2

mM) and $MgCl_2$ (5 mM) in 20 mM HEPES, 150 mM NaCl, and 5 mM TCEP (pH 7.5) at 37 °C for 45 min. For anti-FLAG detection of URM1-containing intermediates and thioester diagnostics (Supplementary Figure 6C–H), unless otherwise indicated reactions contained FLAG–URM1WT–GG (20 μM), NAE1/UBA3 (5 μM), and UBE2M (3 μM) with ATP (5 mM) and $MgCl_2$ (10 mM) in 50 mM HEPES, 150 mM NaCl, 5 mM TCEP (pH 7.5) and were incubated at 37 °C for 3 h. Where indicated, UBE2M was omitted to generate the E1~URM1 species and ATP was omitted for ATP-dependence controls. To assess catalytic cysteine dependence, enzymes were pretreated with N-ethylmaleimide (NEM; 2 mM) prior to initiating the charging reaction and excess NEM was removed using a spin desalting column. To probe thiol sensitivity, reaction mixtures were challenged post-reaction with glutathione (GSH; 5 mM) or with DTT (2 mM) or β-mercaptoethanol (βME; 2 mM); for DTT/βME challenges, samples were incubated for 1 h at 37 °C prior to quenching. All reactions were quenched in non-reducing SDS sample buffer, resolved by SDS–PAGE, and analyzed by immunoblotting with anti-UBE2M (Supplementary Figure 6B) or anti-FLAG (Supplementary Figure 6C–H).

NEDD8-GG competition assays. To test competition between URM1-WT–GG and NEDD8-GG for NAE1/UBA3-dependent charging, reactions were assembled as either complete reactions (FLAG–URM1WT–GG + ATP + NAE1/UBA3 + UBE2M) or E1-only reactions (FLAG–URM1WT–GG + ATP + NAE1/UBA3; UBE2M omitted) using the conditions described above for URM1-containing intermediates (FLAG–URM1WT–GG 20 μM; NAE1/UBA3 5 μM; UBE2M 3 μM where applicable; ATP 5 mM; $MgCl_2$ 10 mM; 50 mM HEPES, 150 mM NaCl, 5 mM TCEP, pH 7.5). NEDD8-GG (20 μM) was added at reaction initiation, and mixtures were incubated at 37 °C for 3 h. Reactions were quenched and analyzed by non-reducing SDS–PAGE followed by anti-FLAG immunoblotting.

Cell viability assay and IC_{50} calculation. HepG2 cells were seeded in 96-well plates and treated with MLN4924 or cisplatin either alone or in combination. For monotherapy, MLN4924 was applied for 72 hours and cisplatin for 48 hours. For combination groups, MLN4924 was added 24 hours prior to cisplatin, resulting in a total MLN4924 exposure of 72 hours and cisplatin exposure of 48 hours. Each treatment condition was tested in triplicate wells, and all experiments were independently repeated three times. Cell viability was assessed using the Cell Counting Kit-8 (MCE) per the manufacturer's instructions. Absorbance at 450 nm was measured using a microplate reader (BioTek Synergy H1), and readings were normalized to vehicle-treated controls. IC_{50} values and 95% confidence intervals (CI) were calculated using nonlinear regression (four-parameter logistic model) in GraphPad Prism 9.
