# Supplementary material for: NAE1/UBA3-UBE2M are E1 and E2 enzymes for the URM1 modification
Source: Nat Commun. 2026 Apr 29;17:5858. doi: 10.1038/s41467-026-72296-w (PMC13333801; doi:10.1038/s41467-026-72296-w)
Supplement: Supplementary file 2 — Reporting Summary [file 41467_2026_72296_MOESM2_ESM.pdf]

nature portfolio

Corresponding author(s): Wenyue Cao, Wenshe Ray Liu

Last updated by author(s): Mar 23, 2026

# Reporting Summary

Nature Portfolio wishes to improve the reproducibility of the work that we publish. This form provides structure for consistency and transparency in reporting. For further information on Nature Portfolio policies, see our Editorial Policies and the Editorial Policy Checklist.

## Statistics

For all statistical analyses, confirm that the following items are present in the figure legend, table legend, main text, or Methods section.

| n/a | Confirmed | |
|---|---|---|
| ☐ | ☒ | The exact sample size ($n$) for each experimental group/condition, given as a discrete number and unit of measurement |
| ☐ | ☒ | A statement on whether measurements were taken from distinct samples or whether the same sample was measured repeatedly |
| ☐ | ☒ | The statistical test(s) used AND whether they are one- or two-sided<br>*Only common tests should be described solely by name; describe more complex techniques in the Methods section.* |
| ☐ | ☒ | A description of all covariates tested |
| ☐ | ☒ | A description of any assumptions or corrections, such as tests of normality and adjustment for multiple comparisons |
| ☐ | ☒ | A full description of the statistical parameters including central tendency (e.g. means) or other basic estimates (e.g. regression coefficient) AND variation (e.g. standard deviation) or associated estimates of uncertainty (e.g. confidence intervals) |
| ☐ | ☒ | For null hypothesis testing, the test statistic (e.g. $F$, $t$, $r$) with confidence intervals, effect sizes, degrees of freedom and $P$ value noted<br>*Give P values as exact values whenever suitable.* |
| ☒ | ☐ | For Bayesian analysis, information on the choice of priors and Markov chain Monte Carlo settings |
| ☒ | ☐ | For hierarchical and complex designs, identification of the appropriate level for tests and full reporting of outcomes |
| ☐ | ☒ | Estimates of effect sizes (e.g. Cohen's $d$, Pearson's $r$), indicating how they were calculated |

*Our web collection on statistics for biologists contains articles on many of the points above.*

## Software and code

Policy information about availability of computer code

| | |
|---|---|
| Data collection | Data were collected using commercial instrument control software provided by the manufacturers. LC-MS/MS proteomics data were acquired on an Orbitrap Fusion Lumos mass spectrometer coupled to an EASY-nLC 1000 system, both operated using Thermo Scientific Xcalibur software (version 4.3). High-field asymmetric waveform ion mobility spectrometry (FAIMS) was controlled using the vendor's FAIMS Pro interface. Flow cytometry data were collected on a CytoFLEX flow cytometer (Beckman Coulter) using CytExpert software (version 2.4). |
| Data analysis | Proteomics raw data were processed with MaxQuant software (version 2.1.2.0) using the UniProt human proteome database (UP000005640) with cysteine carbamidomethylation as a fixed modification, and methionine oxidation and N-terminal acetylation as variable modifications. RNA-seq data were quality-filtered with fastp (version 0.20.0), aligned to the GRCh38 human genome using HISAT2 (version 2.0.5), quantified with featureCounts (version 1.5.0-p3), and analyzed for differential expression using the DESeq2 R package (version 1.20.0). Gene Ontology and KEGG pathway enrichment analyses were performed using clusterProfiler (version 3.8.1). Gene set enrichment analysis (GSEA) was conducted with the Broad Institute's GSEA software (version 4.2.3). Flow cytometry data were analyzed with FlowJo software (version 10.8.1). Western blot band intensities were quantified using Image Lab software (Bio-Rad, version 6.1). Proteomic heatmaps were generated using GraphPad Prism 10, and supplementary $IC_{50}$ analysis was performed using GraphPad Prism 9. |

For manuscripts utilizing custom algorithms or software that are central to the research but not yet described in published literature, software must be made available to editors and reviewers. We strongly encourage code deposition in a community repository (e.g. GitHub). See the Nature Portfolio guidelines for submitting code & software for further information.

## Data

Policy information about availability of data

All manuscripts must include a data availability statement. This statement should provide the following information, where applicable:

- Accession codes, unique identifiers, or web links for publicly available datasets
- A description of any restrictions on data availability
- For clinical datasets or third party data, please ensure that the statement adheres to our policy

Data availability. The RNA-seq data generated in this study have been deposited in the Gene Expression Omnibus (GEO) under accession code GSE305835 https://www.ncbi.nlm.nih.gov/geo/query/acc.cgi?acc=GSE305835. The mass spectrometry proteomics data generated in this study have been deposited in the ProteomeXchange Consortium via the PRIDE partner repository under accession code PXD067405 http://proteomecentral.proteomexchange.org/cgi/GetDataset?ID=PXD067405. The TCGA data used in this study are available from the Genomic Data Commons Data Portal https://portal.gdc.cancer.gov. Source data are provided with this paper. All other data supporting the findings of this study are available within the paper and its Supplementary Information.

## Research involving human participants, their data, or biological material

Policy information about studies with human participants or human data. See also policy information about sex, gender (identity/presentation), and sexual orientation and race, ethnicity and racism.

| | |
|---|---|
| Reporting on sex and gender | N/A.<br>This study involved immortalized human cell lines and mouse experiments only; no human participants, primary human tissues, or individual-level human data were used. |
| Reporting on race, ethnicity, or other socially relevant groupings | N/A.<br>No human participants or socially relevant groupings were included; public datasets used were fully de-identified. |
| Population characteristics | N/A. |
| Recruitment | N/A. |
| Ethics oversight | N/A. |

Note that full information on the approval of the study protocol must also be provided in the manuscript.

# Field-specific reporting

Please select the one below that is the best fit for your research. If you are not sure, read the appropriate sections before making your selection.

☒ Life sciences ☐ Behavioural & social sciences ☐ Ecological, evolutionary & environmental sciences

For a reference copy of the document with all sections, see nature.com/documents/nr-reporting-summary-flat.pdf

# Life sciences study design

All studies must disclose on these points even when the disclosure is negative.

| | |
|---|---|
| Sample size | Sample size is indicated in the figure legends. No statistical methods were used to predetermine sample sizes. |
| Data exclusions | No data were excluded from the analyses. |
| Replication | All key experiments were repeated independently at least twice, and results were reproducible. The exact number of independent biological replicates for each experiment is stated in the figure legends. |
| Randomization | No randomizations were undertaken. |
| Blinding | Blinding was performed for bulk RNA-seq data collection. |

# Reporting for specific materials, systems and methods

We require information from authors about some types of materials, experimental systems and methods used in many studies. Here, indicate whether each material, system or method listed is relevant to your study. If you are not sure if a list item applies to your research, read the appropriate section before selecting a response.

## Materials & experimental systems

| n/a | Involved in the study | |
|---|---|---|
| | x | Antibodies |
| | x | Eukaryotic cell lines |
| x | | Palaeontology and archaeology |
| | x | Animals and other organisms |
| x | | Clinical data |
| x | | Dual use research of concern |
| x | | Plants |

## Methods

| n/a | Involved in the study | |
|---|---|---|
| x | | ChIP-seq |
| | x | Flow cytometry |
| x | | MRI-based neuroimaging |

# Antibodies

Antibodies used

For Western blot
Anti-Flag (Mouse, monoclonal, Millipore Sigma, Cat#A8592, 1:2000 dilution)
Anti-URM1 (Rabbit, polyclonal, Proteintech, Cat#15285-1-AP, 1:1000 dilution)
Anti-UBA3 (Mouse, IgG2a, Thermo Fisher Scientific, Cat#MA536104, 1:500 dilution)
Anti-NAE1 (Mouse, IgG, Thermo Fisher Scientific, Cat#H00008883-B01P, 1:500 dilution)
Anti-UBE2M (Mouse, IgG1, Thermo Fisher Scientific, Cat#MA525739, 1:1000 dilution)
Goat anti-Rabbit IgG (H+L), HRP-conjugated (Proteintech, Cat#SA00001-2, 1:2000 dilution)
Goat anti-Mouse IgG2a, HRP-conjugated (Thermo Fisher Scientific, Cat#M32207, 1:2000 dilution)
Goat anti-Mouse IgG (H+L), HRP-conjugated (ABClonal, Cat#AS003, 1:2000 dilution)
Goat anti-Mouse IgG1, HRP-conjugated (Thermo Fisher Scientific, Cat#PA1-74421, 1:2000 dilution)

For flow cytometry
APC Annexin V Apoptosis Detection Kit with PI (BioLegend, Cat#640932, used according to manufacturer's instructions).

Validation

For western blotting, anti-FLAG (Millipore Sigma, Cat# A8592) was used to detect FLAG-tagged proteins; according to the manufacturer, this clone recognizes the FLAG epitope in N-terminal, C-terminal, and internal contexts and is validated for WB detection of FLAG fusion proteins. Anti-URM1 (Proteintech, Cat# 15285-1-AP) is validated by the manufacturer for western blotting, including WB images in HepG2 and other lysates. Anti-UBA3 (Thermo Fisher Scientific, Cat# MA5-36104) is validated by the manufacturer for WB (recommended WB dilution 1:500–1:2,000). Anti-NAE1 (Thermo Fisher Scientific, Cat# H00008883-B01P) is validated by the manufacturer for WB, including detection in transfected 293T lysates. Anti-UBE2M (Thermo Fisher Scientific, Cat# MA5-25739) is validated by the manufacturer for WB, including detection in HepG2, HeLa, and HEK293T lysates. In addition, in this study, anti-UBA3, anti-NAE1, and anti-UBE2M gave signals at the expected molecular weights and showed the expected changes in probe-enrichment and/or knockdown experiments, providing study-specific support for their use in WB. For flow cytometry, the APC Annexin V Apoptosis Detection Kit with PI (BioLegend, Cat# 640932) was used according to the manufacturer's instructions; this kit is specifically designed for flow-cytometric identification of apoptotic and necrotic cells.

Anti-FLAG (Millipore Sigma, A8592):
https://www.sigmaaldrich.com/US/en/product/sigma/a8592

Anti-URM1 (Proteintech, 15285-1-AP):
https://www.ptglab.com/products/URM1-Antibody-15285-1-AP.htm

Anti-UBA3 (Thermo Fisher Scientific, MA5-36104):
https://www.thermofisher.com/antibody/product/UBA3-Antibody-clone-1B5-2-3-Monoclonal/MA5-36104

Anti-NAE1 (Thermo Fisher Scientific, H00008883-B01P):
https://www.thermofisher.com/order/catalog/product/de/de/H00008883-B01P

Anti-UBE2M (Thermo Fisher Scientific, MA5-25739):
https://www.thermofisher.com/antibody/product/UBE2M-Antibody-clone-OTI2D9-Monoclonal/MA5-25739

APC Annexin V Apoptosis Detection Kit with PI (BioLegend, 640932):
https://www.biolegend.com/en-dk/products/apc-annexin-v-apoptosis-detection-kit-with-pi-9788

# Eukaryotic cell lines

Policy information about cell lines and Sex and Gender in Research

Cell line source(s)

HEK293T and HeLa cells were obtained from the American Type Culture Collection (ATCC), and HepG2 cells were obtained from the Margie Moczygemba laboratory (Texas A&M University) and were originally sourced from ATCC.

Authentication

All cell lines were purchased from authenticated sources or obtained from reputable collaborating laboratories.

Mycoplasma contamination

All cell lines tested negative for mycoplasma contamination using PCR-based mycoplasma detection.

| | |
|---|---|
| Commonly misidentified lines (See ICLAC register) | No commonly misidentified cell lines listed in the ICLAC register were used in this study. |

## Animals and other research organisms

Policy information about studies involving animals; ARRIVE guidelines recommended for reporting animal research, and Sex and Gender in Research

| | |
|---|---|
| Laboratory animals | Male C57BL/6J mice (3 months old) were used in this study. Mice were maintained at the Texas A&M University animal facility, and tissues collected included liver, lung, kidney, and brain. |
| Wild animals | The study did not involve wild animals. |
| Reporting on sex | Male mice were used. The study involved tissue collection only and was not designed or powered to test sex differences; therefore, sex-based analyses were not performed. |
| Field-collected samples | The study did not involve samples collected from the field. |
| Ethics oversight | All animal procedures were approved by the Texas A&M University Institutional Animal Care and Use Committee (IACUC) under protocol IACUC 2025-0071 and were conducted in accordance with the NIH Guide for the Care and Use of Laboratory Animals. |

Note that full information on the approval of the study protocol must also be provided in the manuscript.

## Plants

| | |
|---|---|
| Seed stocks | N/A. |
| Novel plant genotypes | N/A. |
| Authentication | N/A. |

## Flow Cytometry

### Plots

Confirm that:

- [x] The axis labels state the marker and fluorochrome used (e.g. CD4-FITC).
- [x] The axis scales are clearly visible. Include numbers along axes only for bottom left plot of group (a 'group' is an analysis of identical markers).
- [x] All plots are contour plots with outliers or pseudocolor plots.
- [x] A numerical value for number of cells or percentage (with statistics) is provided.

### Methodology

| | |
|---|---|
| Sample preparation | HepG2 cells were transfected with URM1 siRNA (Lipofectamine RNAiMAX). After 48 h, cells were treated with 200 µM $H_2O_2$ or 400 µM diamide for 16 h, then harvested, washed twice with PBS, and stained with APC-Annexin V and PI using the BioLegend kit per the manufacturer's instructions. Samples were analyzed immediately. |
| Instrument | Flow cytometry was performed on a CytoFLEX flow cytometer (Beckman Coulter) |
| Software | Data were analyzed using FlowJo. |
| Cell population abundance | A minimum of 20,000 events was collected per sample; n = 7 biological replicates were analyzed for each condition. |
| Gating strategy | Live cells were defined as Annexin $V^{-}/PI^{-}$; gates were set using unstained and single-stained controls. Heat-killed cells were included as a positive control. |

- [x] Tick this box to confirm that a figure exemplifying the gating strategy is provided in the Supplementary Information.
