# Supplementary material for: NAE1/UBA3-UBE2M are E1 and E2 enzymes for the URM1 modification
Source: Nat Commun. 2026 Apr 29;17:5858. doi: 10.1038/s41467-026-72296-w (PMC13333801; doi:10.1038/s41467-026-72296-w)
Supplement: Supplementary file 3 — Transparent Peer Review file [file 41467_2026_72296_MOESM3_ESM.pdf]

nature portfolio

Peer Review File

NAE1/UBA3-UBE2M are E1 and E2 Enzymes for the URM1 Modification

Corresponding Author: Professor Wenshe Liu

This file contains all reviewer reports in order by version, followed by all author rebuttals in order by version.

Version 0:

Reviewer comments:

Reviewer #1

(Remarks to the Author)
This is a very nice straightforward paper that identifies the enzymes required for activation and transfer of the Ub-like modifier Urm1. The authors make a valiant attempt to link Urmylation, or the absence thereof, to a stress response. Based on historic data obtained from the TCGA atlas, the authors also note a difference in survival of patients with tumors expressing high or low levels of Urmylation. I find the data compelling and techniically well executed. The data are interpreted correctly and do not stray beyond what the actual data demonstrate. The authors properly reviewed the literature and citations to earlier work on Urm1 are appropriate. All in all, this is definitely a paper worth publishing in NComms. The only point of debate might be the somewhat limited amount of data that speak to functional relevance, but this criticism could also be leveled at much of the earlier work on. Urm1. I do not see the need for further experimentation.

Reviewer #2

(Remarks to the Author)
In this manuscript, Chakraborty et al. report the design of an activity-based URM1 probe to interrogate the human URMylation pathway. Using this probe in proteomic screens, the authors identify NEDD8 E1 and the NEDD8 E2, UBE2M as candidate URM1 E1 and E2 enzymes and implicate DCN1 as a potential E3 ligase. Through a combination of biochemical pull-downs, RNAi knockdowns, inhibitor studies, and transcriptomic profiling, they propose that URM1 modification in human cells proceeds via a canonical E1-E2-E3 cascade involving the same machinery that drives NEDDylation. They further suggest that this pathway protects against oxidative stress, influences tumor cell survival, and may be of therapeutic relevance. While there are many positive aspects to this work, particularly the novel probe design and broad dataset integration, the central claim that the NEDDylation machinery also catalyzes URM1 modification is highly provocative and will require substantial additional validation before it can be accepted as definitive. A key conceptual concern is whether URM1 is genuinely activated by the NEDDylation machinery in cells or whether the observed interactions reflect non-physiological permissiveness of the E1/E2 enzymes. Because NEDD8 is the native substrate for NAE1/UBA3 and UBE2M and is present at higher abundance, it is important to demonstrate that URM1 can be activated and transferred using canonical reaction chemistry and in competition with NEDD8. Otherwise, there remains the possibility that the reported URM1 conjugation represents forced or non-native chemistry detectable in vitro or under probe-driven conditions, rather than a bona fide physiological pathway. Overall, this is an interesting and potentially important study, but substantial additional mechanistic validation will be essential to establish the physiological relevance of the proposed URM1 modification pathway.

Major comments:
1) While the authors report the formation of UBA3-URM1 and UBE2M-URM1 complexes in vitro and observe corresponding bands by Western blot, these data do not establish that URM1 undergoes bona fide thioester-based activation and transfer. The weakness of the current evidence is that covalent complexes formed with the activity-based probe could represent stable adducts rather than the transient intermediates expected in a canonical E1–E2 cascade. To address this, the authors should reconstitute the pathway biochemically using purified components and native URM1-GG, demonstrating ATP-dependent charging of both UBA3 and UBE2M with URM1 and confirming the thioester nature of these adducts by sensitivity to reducing agents and hydroxylamine. Including catalytic cysteine mutants of UBA3 and UBE2M that fail to charge would further support the specificity of the reaction. Such direct reconstitution is necessary to validate the central mechanistic claim that the NEDDylation machinery functions as the E1-E2 pair for URM1.

2) Although NEDD8 is present in the cell and tissue lysates used for the probe experiments, the lysate setting does not preserve the competitive and compartmentalized environment of intact cells. Under reducing and probe-driven conditions, non-physiological interactions may occur. Because NEDD8 is the native substrate for NAE1/UBA3 and UBE2M and is present at higher abundance, it is important to demonstrate that URM1 can be activated and transferred in competition with NEDD8 under cellular conditions. Otherwise, there remains the possibility that the reported URM1 conjugation represents forced or non-native chemistry detectable in vitro or under probe-driven conditions rather than a bona fide physiological pathway. To address this, direct kinetic comparisons of NEDD8 versus URM1 charging and transfer with purified enzymes, as well as competition assays, would be essential to establish whether URM1 can meaningfully compete with NEDD8 for activation in a physiological context.

3) DCN1 is a well-established scaffold that promotes NEDDylation of Cullin-RING ligases through its interaction with UBE2M, and while the inhibitor data under oxidative stress are intriguing, they remain indirect and do not firmly establish DCN1 as the E3 for URM1. To elevate DCN1 from a candidate to a definitive E3, the authors should include genetic experiments in which DCN1 is depleted by knockdown or CRISPR and rescued with wild-type DCN1 versus a mutant defective in UBE2M binding. In addition, in-vitro reconstitution showing DCN1-dependent transfer of URM1 from UBE2M~URM1 to a defined substrate lysine, with mass spectrometric confirmation of a URM1-Lys isopeptide, would provide direct evidence for enzymatic function.

4) Many of the approaches used in this study, including MLN4924, UBE2M perturbation, and DCN1 inhibition, primarily target the NEDDylation pathway, so the observed decreases in anti-URM1 signal could be explained by indirect effects on proteostasis or redox balance rather than direct inhibition of URM1 conjugation. To strengthen the argument, the authors should incorporate URM1-specific readouts, such as immunoprecipitation with anti-URM1 followed by diGly remnant enrichment and mass spectrometric site mapping to identify URM1-modified lysines. They should also include a rescue experiment in which a MLN4924-resistant NAE mutant is tested for restoration of URM1 conjugation or, alternatively, deplete NEDD8 and assess whether URM1 modification persists. Finally, testing whether a NEDD8-based activity probe captures the same bands or targets would help demonstrate that the signals attributed to URM1 are distinct from those arising from NEDDylation.

5) The propargylamine warhead is reactive toward catalytic cysteines in general, so it is important to demonstrate that the observed interactions with FLAG-URM1-G101Pa are not simply due to broad cysteine reactivity but rather reflect true pathway specificity conferred by URM1. To establish this, the authors should include side-by-side activity-based probes derived from other UBLs such as NEDD8, SUMO, or ubiquitin as negative or orthogonal controls in both the proteomics and immunoprecipitation experiments. In addition, competition assays in which lysates or recombinant targets are pre-incubated with excess non-reactive URM1-GG compared to an unrelated UBL would help demonstrate whether probe labeling is selectively displaced by URM1, thereby supporting pathway-specific recognition.

6) NEDD8 E1 and UBE2M catalytic cysteine mutants should be tested in cell-based assays to demonstrate that URM1 conjugation depends on the enzymatic activity of UBA3 and UBE2M in vivo, rather than reflecting indirect effects of knockdown or inhibition. Such direct reconstitution and validation are necessary to substantiate the central mechanistic claim that the NEDDylation machinery functions as the E1–E2 cascade for URM1.

7) The methods and results for Figure S6 are not sufficiently clear. The band interpreted as the UBE2M-URM1 product appears as only a minor fraction relative to free UBE2M, and it remains uncertain whether this band truly represents the expected UBE2M-URM1 thioester intermediate in a canonical E1–E2–E3 cascade. Incorporating catalytic cysteine mutants and including control lanes with and without reducing agents would help clarify the identity of this species and strengthen the mechanistic interpretation.

Reviewer #3

(Remarks to the Author)
Among Ub-like modifiers (Ubls), Urm1 is one of the least studied. It is a small Ubl protein with the typical C-terminal diglycine and β-grasp fold motif. Initially shown to mainly modify the Ahp1 protein in budding yeast via the E1-like Uba4, it was later found to conjugate with multiple proteins in yeast and humans under oxidative stress. Like other Ubls, it attaches to Lys residues, targeting proteins involved in RNA binding, tRNA modification, nuclear transport, and stress response. Unlike Ub and Ubls, which depend on E1–E2–E3 cascades, Urm1 is activated to a thiocarboxylate (-COSH) and is thought to proceed through urmylation by an unusual, possibly E2/E3-independent mechanism. No Urm1 chains have been detected, and unlike ubiquitin or SUMO, no de-urmylating enzyme is known to exist.
In this manuscript submitted to Nature Communications, the Liu group uses a chemical biology approach based on an activity-based Urm1 probe (FLAG-URM1-G101Pa). The team combines it with advanced proteomics to report the identification of the first dedicated E1-E3 enzymes responsible for urmylation in human cells, establishing the NAE1/UBA3–UBE2M–DCN1 axis as the main catalytic pathway for this PTM. The work includes multiple independent validation steps, covering structural probe development, proteomics, gene knockdown, inhibitor studies, and functional transcriptomic and clinical correlations. Notably, this study reveals that the urmylation machinery overlaps with the neddylation pathway, which has implications for understanding the action of small-molecule inhibitors, such as MLN4924, in cancer therapy. These findings greatly enhance our understanding of stress adaptation, posttranslational regulation, and cancer biology, and open new avenues for therapies targeting the urmylation machinery.

I recommend the publication of this manuscript after the authors address these comments:

1) The study on the deconjugating proteases for protein urmylation is not comprehensive and can be misleading. The fact that some DUBs reacted with URM1-G101Pa does not make them become deurmylases, given structural similarities between URM1 and ubiquitin, covalent interactions between these enzymes and FLAG-URM1-G101Pa, which could lead to non-selective labeling. This is also true even though these enzymes removed the ACA unit from the URM1-ACA. This study requires further cellular work and validation to confirm its findings. I suggest removing this section and reserving it for a more comprehensive analysis, similar to the job done with E1-E3.

2) The discussion acknowledges the possibility of a thiocarboxylate-dependent (yeast-like) conjugation pathway for Urm1 in mammalian cells. Are there experimental indications for or against dual-pathway usage under stress or developmental conditions in humans? Please note that a recent study published in Angew Chem has shown that, regardless of URM1 C-terminal functionality, URM1 forms fewer conjugates under normal conditions compared to conditions of oxidative stress, along with the recent in vitro studies, suggesting that the characteristic GG motif is not required for URM1's conjugation. The author should cite this work in the context of the suggested thioacid role, as it is particularly relevant to the work presented here, which utilizes chemical biology approaches to study URM (https://doi.org/10.1002/anie.202410135).

3) Can the authors comment on whether they can delineate the substrate specificity determinants for Urm1 versus NEDD8? And the endogenous protein substrates modified by Urm1 vs the Nedd8.

4) It's unclear from the data how effective the 2-nitro-5-thiocyanobenzoic acid (NTCB) method is at activating a protein cysteine for an exchange reaction. Comparing the mass of the starting material to the product doesn't provide enough information. HPLC analysis should be used to demonstrate the efficiency of the conversion.

5) A recent review of Ubl chemical biology approaches has been published and can be cited to show the different tools to study this family (RSC Chem. Biol., 2025,6, 492-509).

Version 1:

Reviewer comments:

Reviewer #2

(Remarks to the Author)
In this revised manuscript, Chakraborty et al. have made a substantial effort to address concerns raised during the initial review. The authors present a large body of newly added experiments that significantly strengthen the work and address many of the issues previously raised, and this effort is appreciated. With that said, the collective evidence still falls short of establishing URM1 as a bona fide physiological/cognate substrate of the neddylation machinery in cells. The new data demonstrate permissive enzymatic interactions but do not yet convincingly address reaction efficiency, competition with the native substrate NEDD8, or native pathway flux under physiological conditions.

This issue is particularly important given historical precedent in the Ub and Ubl fields. For example, the E2 enzyme UBE2L6 was initially annotated as a Ub E2 based on its ability to form thioester intermediates with the Ub E1 UBA1 in vitro. Subsequent work, however, demonstrated that UBE2L6 is in fact the cognate E2 for the ISG15 E1 UBA7/UBE1L in vivo, and that its apparent engagement with UBA1 reflected biochemical permissiveness rather than physiological pathway usage (see PMID: 18583345).

In the present study, even at the in vitro level, the data do not yet meet the standard typically used to assign a cognate Ubl-E1-E2 relationship, as reaction efficiency, stoichiometry, and competition with the native substrate have not been established. This underscores the importance of distinguishing permissive E1-E2 chemistry from bona fide pathway flux when assigning enzymatic cascades in cells. Based on the current evidence, in this reviewer's view, URM1 representing a bona fide physiological/cognate substrate of the neddylation machinery in cells remains premature and the key mechanistic claims would need to be substantially softened to be supported by the data (for example, by framing URM1 engagement as conditional, stress-associated, or permissive rather than canonical). Otherwise, additional evidence would be required to support the current mechanistic claims.

Specific comments:

1) In Figure S6B, the band interpreted as the UBE2M~URM1 thioester intermediate is present at very low abundance relative to free UBE2M, and key controls required to establish thioester identity are not shown. In particular, no reducing-agent control (+/- DTT or +/- βMe) is included to demonstrate thiol sensitivity of the putative intermediate. Although the authors examine the effect of glutathione in Figure S6E, glutathione is not an ideal substitute for DTT or βMe as a diagnostic

reagent for thioester intermediates, given its lower and context-dependent nucleophilicity.

2) More generally, enzyme and substrate concentrations and reaction time points are not reported for the experiments shown in Figure S6, precluding assessment of stoichiometry, kinetics, or reaction efficiency and leaving open the possibility that high enzyme concentrations may artificially drive non-physiological thioester formation. This concern is heightened by the very low level of UBE2M-URM1 formation evident in Figure S6B. Furthermore, E1 and E2 charging assays are typically presented in a manner that enables quantification of modified versus unmodified enzyme (e.g., Coomassie or Sypro staining, or fluorescently labeled UBLs), rather than relying solely on Western blot detection of a minor species. Because the readout in Figure S6C-G relies exclusively on anti-URM1 (Flag) immunoblotting, the relative levels of free versus charged UBA3 and UBE2M cannot be evaluated.

3) Additionally, no side-by-side NEDD8 controls are included, making it impossible to benchmark URM1 charging efficiency against the native substrate of this E1–E2 pathway. Given that the in vitro system has been established, a minimal and informative experiment would be to include URM1 and NEDD8 at equal concentrations in parallel E1 charging and E2 transfer assays, as well as in direct competition assays, to determine whether URM1 can meaningfully compete with NEDD8 for activation and transfer.

Taken together, the data presented in Figure S6 do not conclusively establish that the observed species represents a bona fide UBE2M~URM1 thioester intermediate rather than a low-abundance species formed under permissive in vitro conditions.

Clarifying these issues is critical because, at the cellular level, most of the evidence for URM1 engagement relies on activity-based probe capture and indirect perturbations (chemical inhibition and knockdown phenotypes), rather than direct observation of endogenous URM1 activation and transfer through the E1–E2 cascade. The authors do not demonstrate endogenous UBA3~URM1 or UBE2M~URM1 thioester intermediates in cells, nor do they show URM1 activation and transfer under conditions that preserve the native competitive environment dominated by NEDD8. As a result, it remains unclear whether the cellular data reflect permissive chemistry revealed by probe-driven or in vitro assays rather than bona fide physiological pathway usage. For these reasons, the biochemical experiments in Figure S6 are pivotal to the central mechanistic claim.

Reviewer #3

(Remarks to the Author)
The authors have revised the comments raised by this referee and to best of my assessment the comments by the other referees. The MS now can be accepted for publication in Nat Com

Open Access This Peer Review File is licensed under a Creative Commons Attribution 4.0 International License, which permits use, sharing, adaptation, distribution and reproduction in any medium or format, as long as you give appropriate credit to the original author(s) and the source, provide a link to the Creative Commons license, and indicate if changes were made.
In cases where reviewers are anonymous, credit should be given to 'Anonymous Referee' and the source.
The images or other third party material in this Peer Review File are included in the article’s Creative Commons license, unless indicated otherwise in a credit line to the material. If material is not included in the article’s Creative Commons

license and your intended use is not permitted by statutory regulation or exceeds the permitted use, you will need to obtain permission directly from the copyright holder.
To view a copy of this license, visit https://creativecommons.org/licenses/by/4.0/

Reviewer #1

Comment: This is a very nice straightforward paper that identifies the enzymes required for activation and transfer of the Ub-like modifier Urm1. The authors make a valiant attempt to link Urmylation, or the absence thereof, to a stress response. Based on historic data obtained from the TCGA atlas, the authors also note a difference in survival of patients with tumors expressing high or low levels of Urmylation. I find the data compelling and techniically well executed. The data are interpreted correctly and do not stray beyond what the actual data demonstrate. The authors properly reviewed the literature and citations to earlier work on Urm1 are appropriate. All in all, this is definitely a paper worth publishing in NComms. The only point of debate might be the somewhat limited amount of data that speak to functional relevance, but this criticism could also be leveled at much of the earlier work on Urm1. I do not see the need for further experimentation.

We thank the reviewer for the supportive and thoughtful assessment of our manuscript, and for recognizing the technical rigor and the care taken to interpret the data within its experimental boundaries. We also appreciate the reviewer's perspective that functional studies of URM1 biology remain relatively limited across the field.

In the revised manuscript, we have made targeted edits to further clarify the scope of our functional conclusions and to better frame key open questions for future work. In discussion part, we added "Although the functional experiments presented here are necessarily initial, they support a cytoprotective role of URM1 under oxidative stress and motivate future substrate-mapping and in vivo genetic studies to define physiological contexts where this pathway is most critical". Overall, we are grateful for the reviewer's recommendation for publication and for the constructive feedback.

Reviewer #2

Comment 1: While the authors report the formation of UBA3-URM1 and UBE2M-URM1 complexes in vitro and observe corresponding bands by Western blot, these data do not establish that URM1 undergoes bona fide thioester-based activation and transfer. The weakness of the current evidence is that covalent complexes formed with the activity-based probe could represent stable adducts rather than the transient intermediates expected in a canonical E1–E2 cascade. To address this, the authors should reconstitute the pathway biochemically using purified components and native URM1-GG, demonstrating ATP-dependent charging of both UBA3 and UBE2M with URM1 and confirming the thioester nature of these adducts by sensitivity to reducing agents and hydroxylamine. Including catalytic cysteine mutants of UBA3 and UBE2M that fail to charge would further support the specificity of the reaction. Such direct reconstitution is necessary to validate the central mechanistic claim that the NEDDylation machinery functions as the E1-E2 pair for URM1.

We thank the reviewer for emphasizing the need to distinguish bona fide E1/E2 charging intermediates from stable/noncovalent complexes. In the revised manuscript and SI, we **strengthened the biochemical evidence** that the observed UBE2M~URM1 and UBA3~URM1 species behave like canonical E1/E2~Ubl thioester-linked charging intermediates.

Specifically, the putative UBE2M~URM1 intermediate is ATP-dependent and is abolished by N-ethylmaleimide (NEM; active-site cysteine blocking) and disrupted by glutathione, consistent with catalytic cysteine dependence and thiol sensitivity (SI Fig. S6C–E). In an E1-only reaction, the UBA3~URM1 intermediate similarly requires ATP and is abolished by NEM (SI Fig. S6F–G). Both intermediates exhibit time- and temperature-dependent formation (SI Fig. S7). We have revised the text to describe these species as charging intermediates consistent with thioester-like behavior, rather than over-interpreting them as stable complexes.

<u>For the reviewer's convenience, we provide the exact revised manuscript text and the relevant updated Supplementary Figure panels below:</u>

(Results, P8) A biochemistry assay containing NAE1/UBA3, UBE2M, URM1, and ATP produced a higher-molecular-weight UBE2M-reactive species under non-reducing conditions that is consistent with a putative UBE2M~URM1 thioester intermediate; omission of ATP, NAE1/UBA3, or URM1 abolished this species (Figure S6B,C). This putative intermediate was eliminated by N-ethylmaleimide and disrupted by glutathione, consistent with catalytic cysteine dependence and thiol sensitivity (Figure S6D,E). In an E1-only reaction, a URM1-containing species consistent with an E1~URM1 thioester intermediate formed in an ATP- and active-site cysteine-dependent manner (Figure S6F,G). Both putative E2~URM1 and E1~URM1 intermediates exhibited time- and temperature-dependent formation (Figure S7). … Together, these

behaviors are consistent with E1/E2~Ubl thioester charging intermediates rather than stable non-covalent complexes.

(Supplementary)

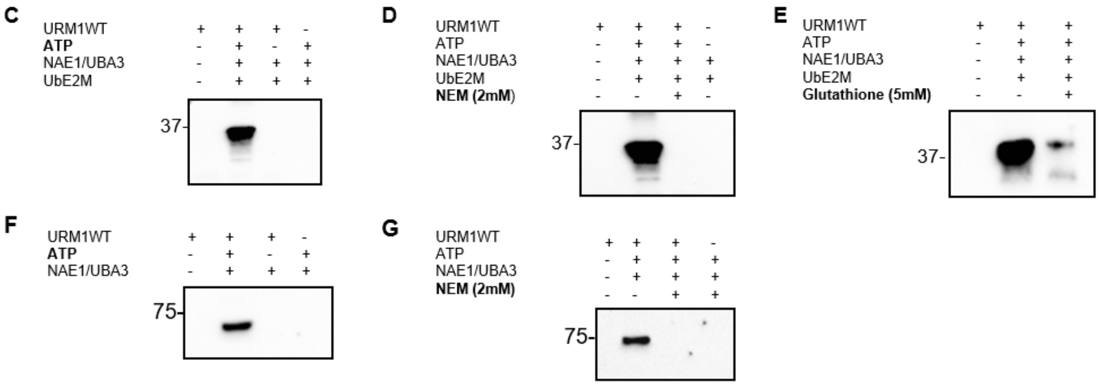

Figure S6. In vitro reconstitution of URM1 charging and thioester-like intermediates. (C–G) The indicated reactions were analyzed by SDS–PAGE under non-reducing conditions and immunoblotted with anti-FLAG to detect URM1-containing intermediates. (C) Component dependence of the putative UBE2M~URM1 species. (D) The putative UBE2M~URM1 species is abolished by N-ethylmaleimide (NEM; 2 mM), consistent with catalytic cysteine dependence. (E) The putative UBE2M~URM1 species is disrupted by glutathione (5 mM), consistent with thiol sensitivity expected for thioester-linked intermediates. (F) Formation of an E1~URM1 thioester intermediate (~75 kDa) requires ATP in reactions containing NAE1/UBA3 and URM1WT. (G) The E1~URM1 intermediate is abolished by NEM (2 mM), consistent with active-site cysteine dependence.

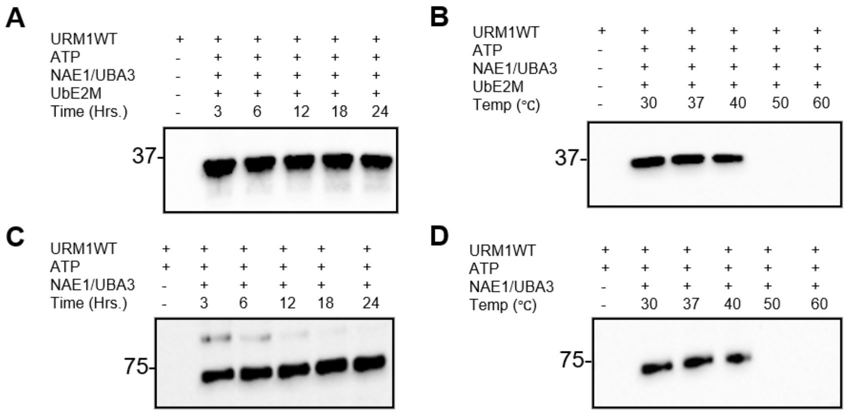

Figure S7. Time- and temperature-dependence of URM1 charging intermediates formed by NAE1/UBA3 and UBE2M. (A) Time-course analysis of a URM1-containing species consistent with the putative UBE2M~URM1 charging intermediate formed in reactions containing FLAG–URM1-GG (URM1WT), ATP, $MgCl_2$, NAE1/UBA3, and UBE2M for the indicated times. (B) Temperature dependence of the putative UBE2M~URM1 intermediate formed at the indicated temperatures in the complete reaction. (C) Time-course analysis of a URM1-containing species consistent with the E1~URM1 thioester intermediate (~75 kDa) formed in an E1-only reaction containing URM1WT, ATP, $MgCl_2$, and NAE1/UBA3 for the indicated times. (D) Temperature dependence of the E1~URM1 intermediate formed at the indicated temperatures in the E1-only reaction. For all panels, reaction mixtures were resolved by SDS–PAGE and analyzed by immunoblotting with an anti-FLAG antibody.

Comment 2: Although NEDD8 is present in the cell and tissue lysates used for the probe experiments, the lysate setting does not preserve the competitive and compartmentalized environment of intact cells. Under reducing and probe-driven conditions, non-physiological interactions may occur. Because NEDD8 is the native substrate for NAE1/UBA3 and UBE2M and is present at higher abundance, it is important to demonstrate that URM1 can be activated and transferred in competition with NEDD8 under cellular conditions. Otherwise, there remains the possibility that the reported URM1 conjugation represents forced or non-native chemistry detectable in vitro or under probe-driven conditions rather than a bona fide physiological pathway. To address this, direct kinetic comparisons of NEDD8 versus URM1 charging and transfer with purified enzymes, as well as competition assays, would be essential to establish whether URM1 can meaningfully compete with NEDD8 for activation in a physiological context.

We agree that the abundance of cellular NEDD8 raises an important question regarding potential competition for shared E1/E2 machinery. We clarify that our study does not aim to conclude global URM1–NEDD8 competition in cells under basal conditions. Rather, our central findings are that the canonical NEDDylation E1/E2 pair can biochemically activate URM1 and that URM1 conjugation is regulated in an oxidative-stress-dependent manner, suggesting context-dependent engagement of this machinery.

To ensure that our conclusions do not rely on probe-driven chemistry alone, we emphasize that URM1 conjugation is inhibited in intact cells by pharmacological inhibition of NAE1/UBA3 (MLN4924) and UBE2M (micafungin), supporting a cellular requirement for this cascade despite the presence of endogenous NEDD8 (Fig. 4H, Fig. S9). In addition, we included **direct *in vitro* competition experiments** using purified components, showing that adding excess NEDD8-GG reduces formation of both UBE2M~URM1 in the complete reaction and UBA3~URM1 in an E1-only reaction (Fig. S8), consistent with URM1 engaging the same charging machinery in a competitive setting. We have also revised the manuscript text to frame these data as evidence of shared enzymatic machinery, while noting that quantitative kinetic preference and the extent of competition in vivo remain to be determined.

<u>For the reviewer's convenience, we provide the exact revised manuscript text and the relevant updated Supplementary Figure panels below:</u>

(Results, P9) Finally, excess NEDD8-GG reduced formation of both intermediates in these in vitro reactions (Figure S8), suggesting that NEDD8 and URM1 can engage the same E1/E2 enzymes when both C-terminal diglycine substrates are present.

(Discussion, P10) Given the abundance of cellular NEDD8, we do not infer global competition between URM1 and NEDD8 in vivo under basal conditions. Substrate or channel selection between neddylation and urmylation may

depend on stress-regulated availability of activated URM1 species, adaptor/E3 usage, and compartmentalization, variables that will require dedicated mechanistic dissection.

(Supplementary)

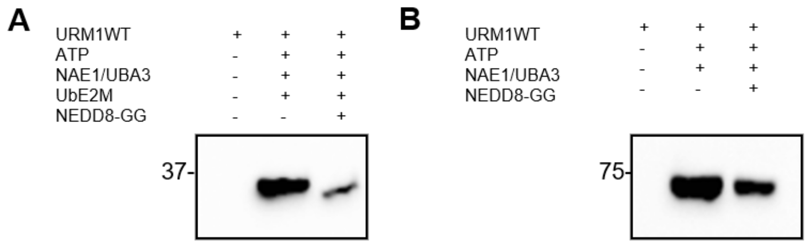

Figure S8. NEDD8-GG competes with URM1-GG in NAE1/UBA3-dependent charging reactions. (A) Formation of UBE2M–URM1 in the complete reaction (URM1WT + ATP + NAE1/UBA3 + UBE2M) is reduced in the presence of NEDD8-GG. (B) Formation of UBA3–URM1 in an E1-only reaction (URM1WT + ATP + NAE1/UBA3) is reduced in the presence of NEDD8-GG. Reaction mixtures were separated by SDS-PAGE and analyzed by immunoblotting with an anti-FLAG antibody.

Comment 3: DCN1 is a well-established scaffold that promotes NEDDylation of Cullin-RING ligases through its interaction with UBE2M, and while the inhibitor data under oxidative stress are intriguing, they remain indirect and do not firmly establish DCN1 as the E3 for URM1. To elevate DCN1 from a candidate to a definitive E3, the authors should include genetic experiments in which DCN1 is depleted by knockdown or CRISPR and rescued with wild-type DCN1 versus a mutant defective in UBE2M binding. In addition, in-vitro reconstitution showing DCN1-dependent transfer of URM1 from UBE2M~URM1 to a defined substrate lysine, with mass spectrometric confirmation of a URM1-Lys isopeptide, would provide direct evidence for enzymatic function.

We thank the reviewer for the careful critique regarding DCN1. We agree that DCN1 is best established in the neddylation field as a scaffold/cofactor that promotes UBE2M-dependent transfer, and that inhibitor phenotypes alone do not constitute definitive proof of DCN1 serving as the URM1 E3. At this stage, we therefore revised the manuscript to frame DCN1 as an E3-like cofactor/candidate, rather than a definitive URM1 E3. Our conclusion is supported by the observation that three mechanistically distinct inhibitors that disrupt the UBE2M–DCN1 interaction (NacM-OPT, DI591, and DI1859) consistently suppress URM1 conjugation under oxidative stress in both HEK293T and HeLa cells (Fig. 5B–H), and also reduce URM1 conjugation under basal conditions (Fig. S9, Fig. S10). We further added explicit language in the Discussion noting that quantitative E3 definition will require the above genetic and biochemical reconstitution experiments.

We also attempted siRNA-mediated DCN1 depletion in HEK293T and HeLa

cells; however, depletion was incomplete across multiple siRNA sequences, preventing a definitive interpretation at this time. Accordingly, we did not include these preliminary results and instead moderated our claims.

For the reviewer's convenience, we provide the revised manuscript text below.

(Results, P9) Collectively, our data provide strong evidence that NAE1/UBA3 and UBE2M are E1 and E2 enzymes for URM1 modification under oxidative stress and UBE2M engages DCN1 as an E3-like scaffold to promote URM1 transfer under oxidative stress. These findings support the existence of an NAE1/UBA3-UBE2M activation cascade and implicate DCN1 as an E3-like cofactor in the cellular URM1 conjugation pathway.

(Discussion, P11) Overall, our results identify NAE1/UBA3 and UBE2M as an E1/E2 activation cascade for URM1 and implicate DCN1 as an E3-like cofactor/scaffold that promotes URM1 conjugation under oxidative stress in human cells.

Comment 4: Many of the approaches used in this study, including MLN4924, UBE2M perturbation, and DCN1 inhibition, primarily target the NEDDylation pathway, so the observed decreases in anti-URM1 signal could be explained by indirect effects on proteostasis or redox balance rather than direct inhibition of URM1 conjugation. To strengthen the argument, the authors should incorporate URM1-specific readouts, such as immunoprecipitation with anti-URM1 followed by diGly remnant enrichment and mass spectrometric site mapping to identify URM1-modified lysines. They should also include a rescue experiment in which a MLN4924-resistant NAE mutant is tested for restoration of URM1 conjugation or, alternatively, deplete NEDD8 and assess whether URM1 modification persists. Finally, testing whether a NEDD8-based activity probe captures the same bands or targets would help demonstrate that the signals attributed to URM1 are distinct from those arising from NEDDylation.

We appreciate the reviewer's concern that several tools used here were originally developed for the neddylation pathway and that decreases in anti-URM1 signal could, in principle, arise indirectly through broader effects on proteostasis or redox signaling. In this revision, we strengthened the manuscript's framing to ensure our conclusions rest on URM1-specific evidence and orthogonal validation, rather than on neddylation-targeting pharmacology alone.

First, our primary biochemical readout is direct detection of URM1 conjugates by anti-URM1 immunoblotting, and the functional phenotypes are supported by URM1 pathway perturbation in parallel with E1/E2 disruption(Fig.4H, Fig. S9-S10). Second, we complement inhibitor effects with independent

biochemical evidence that the neddylation E1/E2 machinery can directly form UBA3~URM1 and UBE2M~URM1 charging intermediates in vitro, including diagnostic ATP- and active-site-cysteine dependence and competition by NEDD8-GG (Fig.S6–S8), thereby anchoring the interpretation in direct enzyme–modifier chemistry. Third, we revised the Results text to explicitly interpret inhibitor phenotypes as supportive while limiting mechanistic claims to what is directly supported by the combined genetic/biochemical dataset. We agree that URM1-centered site-mapping approaches (URM1 enrichment followed by diGly-remnant profiling and site-resolved proteomics), genetic resistance/rescue strategies, and comparative profiling with NEDD8-based activity probes would be powerful next-step experiments to further dissect pathway specificity; we now highlight these as important future directions and have adjusted the manuscript accordingly.

For the reviewer's convenience, we provide the revised manuscript text below.

(Results, P8) Because MLN4924 and the UBE2M-DCN1 interface inhibitors were developed to perturb neddylation, their effects on URM1 conjugation could, in principle, include indirect consequences of broadly altering Cullin-RING ligase (CRL) activity and cellular stress signaling. We therefore interpret inhibitor phenotypes as supportive and consider them alongside orthogonal genetic and biochemical evidence.

Comment 5: The propargylamine warhead is reactive toward catalytic cysteines in general, so it is important to demonstrate that the observed interactions with FLAG-URM1-G101Pa are not simply due to broad cysteine reactivity but rather reflect true pathway specificity conferred by URM1. To establish this, the authors should include side-by-side activity-based probes derived from other UBLs such as NEDD8, SUMO, or ubiquitin as negative or orthogonal controls in both the proteomics and immunoprecipitation experiments. In addition, competition assays in which lysates or recombinant targets are pre-incubated with excess non-reactive URM1-GG compared to an unrelated UBL would help demonstrate whether probe labeling is selectively displaced by URM1, thereby supporting pathway-specific recognition.

We appreciate the reviewer's caution regarding the propargylamine-based activity-based probe chemistry. We agree that terminal-alkyne/propargylamide warheads can react with catalytic cysteines, and thus the central issue is whether enrichment reflects URM1-guided recognition rather than indiscriminate cysteine reactivity. Mechanistically, propargylamide/terminal-alkyne warheads used in Ub/UBL activity-based probes behave as active-site–directed, mechanism-based traps: efficient covalent capture requires productive engagement of the UBL scaffold within the enzyme active site and

leads to a defined vinyl thioether adduct, rather than broad, diffusion-controlled cysteine alkylation (Ekkebus et al., J. Am. Chem. Soc. 2013; Sommer et al., Bioorg. Med. Chem. 2013; Ovaa, Nat. Rev. Cancer 2007; Griffin et al., Nature Communications 2025).

Consistent with this interpretive framework, in the revised manuscript we clarified that the warhead provides covalent capture while specificity is primarily conferred by the URM1 scaffold and active-site engagement, and that probe enrichment should be interpreted as identifying candidate URM1-engaging cysteine enzymes rather than implying inherent warhead selectivity. Importantly, to control for non-covalent URM1/FLAG binders and workflow/background enrichment, our LFQ-MS proteomics was performed side-by-side with a non-reactive negative control (FLAG-URM1-G101C-6His) processed identically; proteins preferentially enriched by FLAG-URM1-G101Pa were defined relative to this control (Fig. 3; Fig. S2).

For the reviewer's convenience, we provide the revised manuscript text below.

(Results, P4) Here, the propargylamide serves primarily as a covalent capture handle, whereas apparent specificity is driven mainly by the URM1 scaffold and active-site engagement.

(Results, P5) Using FLAG-URM1-G101C-6×His-treated samples as controls avoided potential identifications of non-covalent URM1 and FLAG binders.

Comment 6: NEDD8 E1 and UBE2M catalytic cysteine mutants should be tested in cell-based assays to demonstrate that URM1 conjugation depends on the enzymatic activity of UBA3 and UBE2M in vivo, rather than reflecting indirect effects of knockdown or inhibition. Such direct reconstitution and validation are necessary to substantiate the central mechanistic claim that the NEDDylation machinery functions as the E1–E2 cascade for URM1.

We agree that catalytic-cysteine mutants of UBA3 and UBE2M would provide a stringent genetic reconstitution test of E1/E2 enzymatic activity in vivo. While we did not perform mutant rescue in cells in this revision, we strengthened the mechanistic assignment using convergent orthogonal evidence that directly addresses the reviewer's concern about indirect knockdown/inhibitor effects.

First, in cell-based assays, genetic depletion of the NAE1/UBA3 complex (siNAE1 and siUBA3) and of UBE2M (siUBE2M) each leads to a marked reduction of endogenous URM1 conjugates in both HEK293T and HeLa cells (Fig. 4A–F). Second, we complement these knockdown data with pharmacological perturbations at two distinct steps of the cascade: inhibition of NAE1/UBA3 with MLN4924 and inhibition of UBE2M with micafungin both

suppress cellular urmylation in a dose-dependent manner (Fig. 4G–H; Fig. S5). Third, to directly demonstrate enzymatic E1/E2 charging chemistry independent of cellular network effects, we reconstituted URM1 charging in vitro and observed canonical thioester hallmarks: formation of putative E2~URM1 and E1~URM1 intermediates requires ATP and is abolished by catalytic-cysteine alkylation with N-ethylmaleimide, with additional thiol sensitivity to glutathione (Fig. S6B–G; Fig. S7). These biochemical diagnostics support a thioester-based E1/E2 mechanism rather than stable non-covalent association.

Finally, we revised the text to explicitly note that inhibitors developed for neddylation could, in principle, exert indirect effects via broader CRL/stress signaling, and we therefore interpret inhibitor phenotypes as supportive when considered alongside the orthogonal genetic and biochemical evidence presented. In addition, we note that oxidative stress may engage additional URM1 conjugation routes in human cells; whether and how oxidative-stress-induced URM1 conjugation is routed through NAE1/UBA3–UBE2M in all contexts remains an important topic for future study.

<u>For the reviewer's convenience, we provide the revised manuscript text below.</u>

(Results, P8) We note that this in vitro competition reflects enzyme–substrate compatibility under defined conditions and does not necessarily imply direct competition between endogenous NEDD8 and URM1 in cells, where relative abundance, compartmentalization, and regulatory context can be fundamentally different.

(Discussion, P11) Given the robust induction of URM1 conjugation by oxidative stress, an important open question is whether stress-induced URMylation is universally routed through the NAE1/UBA3–UBE2M cascade or whether additional thiocarboxylate-dependent mechanisms contribute under specific contexts.

Comment 7: The methods and results for Figure S6 are not sufficiently clear. The band interpreted as the UBE2M-URM1 product appears as only a minor fraction relative to free UBE2M, and it remains uncertain whether this band truly represents the expected UBE2M-URM1 thioester intermediate in a canonical E1–E2–E3 cascade. Incorporating catalytic cysteine mutants and including control lanes with and without reducing agents would help clarify the identity of this species and strengthen the mechanistic interpretation.

We thank the reviewer for noting that the presentation of Fig. S6 required a clearer description and stronger validation of the putative charging intermediate. In the revised Supplementary Information, we expanded both the Fig. S6 legend and the corresponding Methods to explicitly specify

reaction components/concentrations, incubation conditions, and the non-reducing electrophoresis/sample-handling parameters used to preserve thioester-linked species. Importantly, we added/clarified functional controls supporting that the ~37 kDa band corresponds to the canonical UBE2M~URM1 thioester intermediate: the species is observed only in the complete reaction and is ATP-dependent (Fig. S6C), is eliminated upon catalytic cysteine blockade with NEM (Fig. S6D), and is disrupted by thiol competition with glutathione (Fig. S6E). Analogous controls for the E1 intermediate (UBA3~URM1) are shown in Fig. S6F–G. Finally, consistent with the transient nature of E2 charging, only a minor fraction of total UBE2M is expected to be thioester-linked at steady state, explaining the relative intensity compared to free UBE2M. Together, these clarifications and controls strengthen the assignment and mechanistic interpretation of the intermediates shown in Fig. S6.

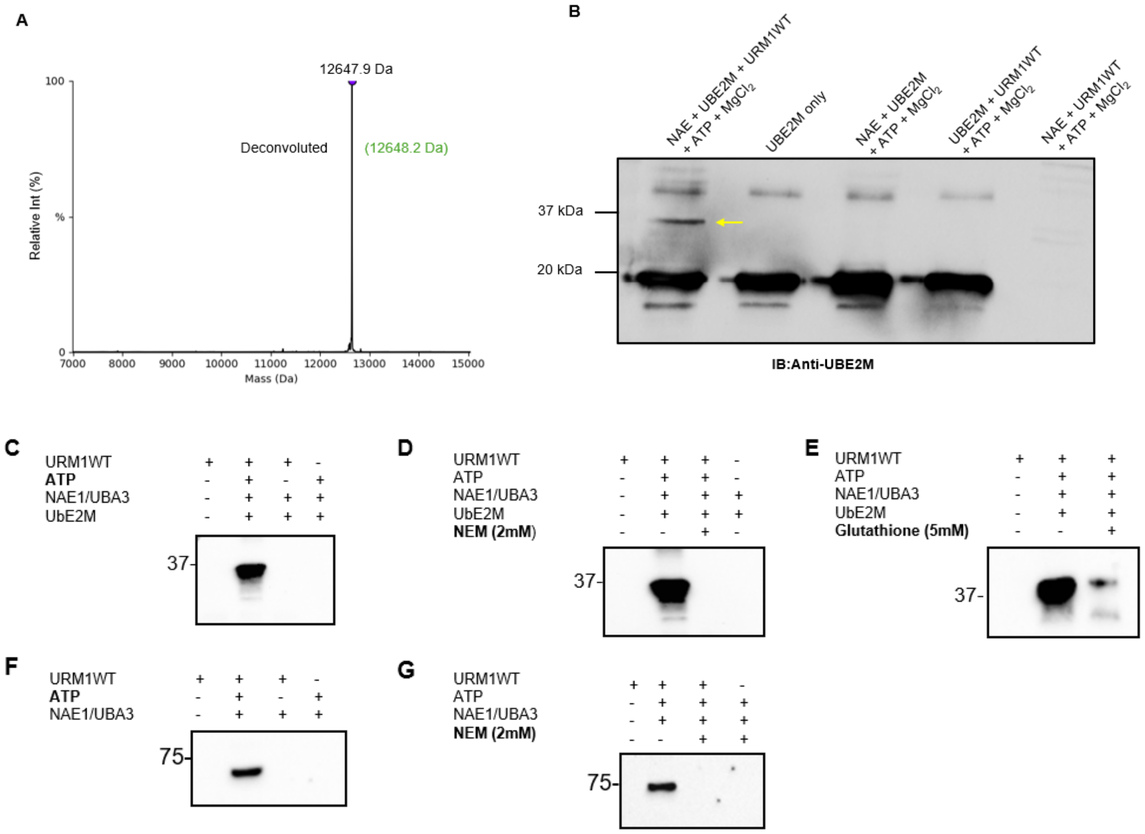

Figure S6. In vitro reconstitution of URM1 charging and thioester-like intermediates. (A) Deconvoluted ESI-MS of wild-type FLAG-URM1-GG (URM1-WT). The theoretical molecular weight of 12648.2 Da (shown in green) was calculated based on its elemental composition. (B) Reconstituted reactions containing URM1WT, ATP, $MgCl_2$, NAE1/UBA3, and/or UBE2M were analyzed by SDS–PAGE under non-reducing conditions and immunoblotted with anti-UBE2M. A higher-molecular-weight UBE2M-reactive species (yellow arrow), consistent with a putative UBE2M~URM1 thioester intermediate, is detected only in the complete reaction and is not observed when essential components are omitted. (C–G) The indicated reactions were analyzed by SDS–PAGE under non-reducing conditions and immunoblotted with anti-FLAG to detect URM1-containing intermediates. (C) Component dependence of the putative

UBE2M~URM1 species. (D) The putative UBE2M~URM1 species is abolished by N-ethylmaleimide (NEM; 2 mM), consistent with catalytic cysteine dependence. (E) The putative UBE2M~URM1 species is disrupted by glutathione (5 mM), consistent with thiol sensitivity expected for thioester-linked intermediates. (F) Formation of an E1~URM1 thioester intermediate (~75 kDa) requires ATP in reactions containing NAE1/UBA3 and URM1WT. (G) The E1~URM1 intermediate is abolished by NEM (2 mM), consistent with active-site cysteine dependence.

Reviewer #3

Comment 1: The study on the deconjugating proteases for protein urmylation is not comprehensive and can be misleading. The fact that some DUBs reacted with URM1-G101Pa does not make them become deurmylases, given structural similarities between URM1 and ubiquitin, covalent interactions between these enzymes and FLAG-URM1-G101Pa, which could lead to non-selective labeling. This is also true even though these enzymes removed the ACA unit from the URM1-ACA. This study requires further cellular work and validation to confirm its findings. I suggest removing this section and reserving it for a more comprehensive analysis, similar to the job done with E1-E3.

We agree with the reviewer that the URM1-ACA hydrolysis assay (Fig. S4) and the URM1-G101Pa profiling should not be interpreted as sufficient evidence to assign physiological "de-URMylase" activity. In the revised manuscript we removed language implying bona fide deurmylase function and instead frame these data as in vitro URM1-substrate compatibility/cross-reactivity consistent with the shared ubiquitin-like fold. Specifically, we revised the Results text to state that, while these enzymes can hydrolyze URM1-ACA in vitro, whether they function as physiological de-URMylating enzymes remains to be determined and will require targeted cellular validation. In parallel, we revised the Supplementary Information figure title/legend for Fig. S4 to describe "URM1-ACA hydrolysis" under in vitro conditions, avoiding terminology that could be misconstrued as establishing endogenous deurmylases.

<u>For the reviewer's convenience, we provide the exact revised manuscript text and the relevant updated Supplementary below:</u>

(Result, P6) All three enzymes efficiently hydrolyze URM1-ACA in vitro, consistent with cross-reactivity expected from URM1/ubiquitin structural similarity; whether they function as physiological de-urmylating enzymes remains to be determined.

(Supplementary) Figure S4. Fluorometric assay of FLAG-URM1-ACA hydrolysis by USP5, USP14, and UCHL5. (A) The structure of FLAG-URM1-ACA. (B) SDS-PAGE analysis of recombinantly expressed FLAG-URM1-G101C-6□His and its ACPL reaction product FLAG-URM1-ACA by reacting with Gly-ACA. (C) Deconvoluted ESI-MS of FLAG-URM1-ACA. Its theoretical molecular weight is 12,846.2 Da. (D) USP5, USP14, and UCHL5-catalyzed ACA release from FLAG-URM1-ACA. Recombinant USP5, USP14, and UCHL5 (50 nM each) were incubated with 400 nM FLAG-URM1-ACA in assay buffer at 30 °C. Fluorescence intensity was recorded every 50 seconds for up to 1 hour (excitation: 380 nm; emission: 460 nm). Compared to control (probe only), all three enzymes triggered a marked increase in fluorescence, indicating cleavage of the FLAG-URM1-ACA probe and indicating hydrolysis

of the URM1-ACA reporter under these in-vitro conditions.

Comment 2: The discussion acknowledges the possibility of a thiocarboxylate-dependent (yeast-like) conjugation pathway for Urm1 in mammalian cells. Are there experimental indications for or against dual-pathway usage under stress or developmental conditions in humans? Please note that a recent study published in Angew Chem has shown that, regardless of URM1 C-terminal functionality, URM1 forms fewer conjugates under normal conditions compared to conditions of oxidative stress, along with the recent in vitro studies, suggesting that the characteristic GG motif is not required for URM1's conjugation. The author should cite this work in the context of the suggested thioacid role, as it is particularly relevant to the work presented here, which utilizes chemical biology approaches to study URM (https://doi.org/10.1002/anie.202410135).

We thank the reviewer for highlighting the possibility of a thiocarboxylate-dependent activation route for URM1 in mammalian systems and for pointing us to the recent Angewandte Chemie study. Our data demonstrate that the NAE1/UBA3–UBE2M machinery can form URM1 charging intermediates in vitro and that perturbing this cascade modulates URM1 conjugation in cells under oxidative stress. At the same time, these results do not exclude alternative URM1 activation chemistries.

To address this explicitly, we revised the Discussion to frame thiocarboxylation as a plausible, context-dependent alternative route in mammals, noting that human MOCS3 is known to activate URM1 for thiocarboxylate formation in related sulfur-transfer biology. We also added the requested citation to the SBL study, which reports that oxidative stress alters URM1 localization and conjugation patterns in live cells. Together with prior in vitro work showing that thiocarboxylated Urm1 can support stress-dependent conjugation chemistry, these studies reinforce that dual-pathway usage remains an open question. We therefore avoid over-interpreting our data as excluding thiocarboxylate-dependent mechanisms and instead highlight this as a key direction for future mechanistic dissection under specific stress or developmental contexts.

<u>For the reviewer's convenience, we provide the revised manuscript text below.</u>

(Discussion, P11) given the robust induction of URM1 conjugation by oxidative stress, an important open question is whether stress-induced URMylation is universally routed through the NAE1/UBA3–UBE2M cascade or whether additional thiocarboxylate-dependent mechanisms contribute under specific contexts. Dissecting the relative contributions of these mechanisms under defined stress or developmental conditions will require future mechanistic studies.

Comment 3: Can the authors comment on whether they can delineate the substrate specificity determinants for Urm1 versus NEDD8? And the endogenous protein substrates modified by Urm1 vs the Nedd8.

We agree that delineating the substrate-specificity determinants that distinguish URM1 from NEDD8, as well as defining the endogenous protein substrates modified by each, is an important mechanistic direction. In the current study, we establish charging-level compatibility of URM1 with components of the canonical neddylation machinery and observe NEDD8–URM1 interplay under defined in vitro conditions; however, we do not claim to have mapped endogenous URM1 substrates or identified substrate-level determinants that differentiate URM1 versus NEDD8 in cells.

To address the reviewer's concern about over-interpreting charging-level observations, we revised both the Results and Discussion to explicitly limit our inference. In the Results, we now note that the observed in vitro competition reflects enzyme–substrate compatibility under defined conditions and does not necessarily imply direct competition between endogenous NEDD8 and URM1 in cells, where relative abundance, compartmentalization, and regulatory context can be fundamentally different. In the Discussion, we further state that given the abundance of cellular NEDD8, we do not infer global competition between URM1 and NEDD8 in vivo under basal conditions, and that substrate/channel selection between neddylation and urmylation may depend on stress-regulated availability of activated URM1 species, adaptor/E3 usage, and compartmentalization—variables that will require dedicated mechanistic dissection.

Finally, because MLN4924 and UBE2M–DCN1 interface inhibitors were developed to perturb neddylation, we also clarified that their effects on URM1 conjugation could include indirect consequences of broadly altering CRL activity and stress signaling. We therefore interpret inhibitor phenotypes as supportive and consider them alongside orthogonal genetic and biochemical evidence presented in the study. Overall, these revisions ensure that our conclusions are confined to what is directly supported by our data while transparently defining the remaining open questions regarding endogenous substrates and specificity determinants.

<u>For the reviewer's convenience, we provide the revised manuscript text below.</u>

(Results, P8) Because MLN4924 and the UBE2M-DCN1 interface inhibitors were developed to perturb neddylation, their effects on URM1 conjugation could, in principle, include indirect consequences of broadly altering Cullin-RING ligase (CRL) activity and cellular stress signaling. We therefore interpret inhibitor phenotypes as supportive and consider them alongside orthogonal genetic and biochemical evidence.

(Discussion, P10) Given the abundance of cellular NEDD8, we do not infer global competition between URM1 and NEDD8 in vivo under basal conditions. Substrate or channel selection between neddylation and urmylation may depend on stress-regulated availability of activated URM1 species, adaptor/E3 usage, and compartmentalization, variables that will require dedicated mechanistic dissection.

(Discussion, P11) Given the robust induction of URM1 conjugation by oxidative stress, an important open question is whether stress-induced URMylation is universally routed through the NAE1/UBA3–UBE2M cascade or whether additional thiocarboxylate-dependent mechanisms contribute under specific contexts. Dissecting the relative contributions of these mechanisms under defined stress or developmental conditions will require future mechanistic studies.

Comment 4: It's unclear from the data how effective the 2-nitro-5-thiocyanobenzoic acid (NTCB) method is at activating a protein cysteine for an exchange reaction. Comparing the mass of the starting material to the product doesn't provide enough information. HPLC analysis should be used to demonstrate the efficiency of the conversion.

We appreciate the reviewer's request for a chromatographic readout to assess the efficiency of the NTCB-enabled cysteine activation/exchange workflow. We agree that intact-mass comparison alone can leave ambiguity regarding conversion and sample purity. To address this, we added an analytical LC–MS chromatographic readout (TIC) for both the starting material (FLAG–URM1–G101C–6×His) and the product probe (FLAG–URM1–G101Pa), alongside intact ESI–MS characterization (Fig. S1A–E). As shown in Fig. S1E, the starting material and product display distinct retention times, and the isolated product sample is dominated by a predominant peak with minimal residual starting material, providing chromatographic support for successful conversion and high purity of the probe used in downstream experiments. We also updated the Methods/SI Methods to explicitly state that LC–MS is used as an orthogonal assessment to complement intact-mass confirmation.

In addition, our use of NTCB-mediated cysteine activation and exchange follows the same chemical-biology framework we recently reported (JACS 2025, DOI: 10.1021/jacs.5c01732), where NTCB-based chemistry is coupled with LC–MS characterization and downstream purification/quality-control to ensure well-defined protein products prior to functional studies. We now cite this work as a precedent for the exchange chemistry and its analytical evaluation, and we emphasize that chromatographic verification is particularly important given that NTCB reactions can exhibit competing side processes under certain conditions.

For the reviewer’s convenience, we provide the updated Supplementary Figure below:

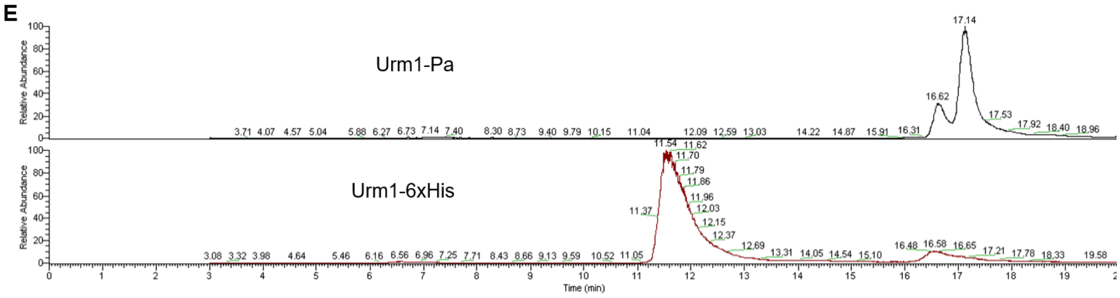

Figure S1. ESI-MS of FLAG-URM1-G101C-6His and FLAG-URM1-G101Pa. (E) Analytical LC–MS chromatograms (TIC) of FLAG-URM1-G101C-6×His (starting material) and FLAG-URM1-G101Pa (product), showing distinct retention times and a predominant product peak, consistent with efficient conversion and high purity.

Comment 5: A recent review of Ubl chemical biology approaches has been published and can be cited to show the different tools to study this family (RSC Chem. Biol., 2025,6, 492-509).

Response: Thank you for pointing us to this timely review. We have added the suggested citation to provide broader context for the chemical biology toolkit used here and to guide readers to complementary approaches for studying ubiquitin-like modifiers.

Reviewer #2

1) In Figure S6B, the band interpreted as the UBE2M~URM1 thioester intermediate is present at very low abundance relative to free UBE2M, and key controls required to establish thioester identity are not shown. In particular, no reducing-agent control (+/- DTT or +/- βMe) is included to demonstrate thiol sensitivity of the putative intermediate. Although the authors examine the effect of glutathione in Figure S6E, glutathione is not an ideal substitute for DTT or βMe as a diagnostic reagent for thioester intermediates, given its lower and context-dependent nucleophilicity.

We agree that a direct reducing-agent challenge strengthens the interpretation. We therefore performed an additional post-reaction reducing-agent challenge and added it as Fig. S6F. After allowing the charging reaction to proceed, we challenged the reaction with DTT (2 mM) or β-mercaptoethanol (2 mM) for 1 h at 37 °C prior to quenching, which reduced/disrupted the putative UBE2M~URM1 species, consistent with the thiol sensitivity expected for a thioester-linked intermediate.

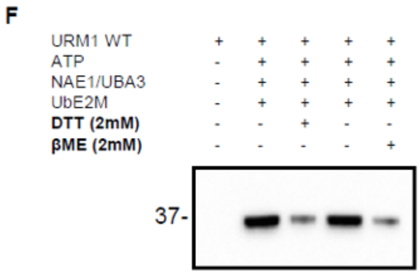

Fig S6. (F) The putative UBE2M~URM1 species is reduced/disrupted upon post-reaction challenge with DTT (2 mM) or β-mercaptoethanol (βME; 2 mM) for 1 h at 37 °C prior to quenching, consistent with thiol sensitivity expected for thioester-linked intermediates.

In the revised manuscript, we also updated the description of the *in vitro* reconstitution results to explicitly include this reducing-agent sensitivity.

2) More generally, enzyme and substrate concentrations and reaction time points are not reported for the experiments shown in Figure S6, precluding assessment of stoichiometry, kinetics, or reaction efficiency and leaving open the possibility that high enzyme concentrations may artificially drive non-physiological thioester formation. This concern is heightened by the very low level of UBE2M-URM1 formation evident in Figure S6B. Furthermore, E1 and E2 charging assays are typically presented in a manner that enables quantification of modified versus unmodified enzyme (e.g., Coomassie or Sypro staining, or fluorescently labeled UBLs), rather than relying solely on Western blot detection of a minor species. Because the readout in Figure S6C-G relies exclusively on anti-URM1 (Flag) immunoblotting, the relative levels of free versus charged UBA3 and UBE2M cannot be evaluated.

We sincerely thank the reviewer for this important technical critique. We carefully read the reviewer-recommended precedent (PMID: 18583345), and it was very helpful in underscoring a central principle in the Ub/Ubl field: *in vitro* thioester formation can reflect biochemical permissiveness, and therefore assigning a cognate E1–E2 relationship in cells requires careful attention to reaction conditions, stoichiometry, and pathway flux in the native competitive environment. We agree that our original presentation of Figure S6 did not provide sufficient experimental detail for the reader to evaluate these aspects, and we have corrected this in the revision.

First, we have now fully reported enzyme/substrate concentrations, buffer composition, temperature, and reaction times directly in the Figure S6 legend and in the SI Methods to make the reaction context explicit. These revisions allow the reader to clearly understand the conditions under which URM1-containing intermediates were observed.

Second, we appreciate the reviewer's suggestion to present E1/E2 charging assays in a format that enables quantification of modified versus unmodified enzyme (e.g., Coomassie/Sypro staining or fluorescently labeled UBLs). Following this advice, we attempted Coomassie-based total protein staining; however, under our experimental workflow the staining did not yield an interpretable readout for these samples, and therefore we did not include it in the revised figure. Therefore, for Figure S6C-H we retained anti-FLAG immunoblotting as a specific readout for URM1 transfer, and we revised the text to avoid any implication that these experiments quantify charging efficiency.

Importantly, to avoid over-interpretation, we also revised the main text (highlighted in red) to explicitly distinguish what Figure S6 is intended to demonstrate: these assays support pathway competence and chemically appropriate URM1 transfer signatures, URM1-containing E1/E2 species under defined conditions, rather than establishing physiological charging efficiency, stoichiometry, or native pathway flux.

Finally, we note that our conclusions are not based on the *in vitro* data alone. The study also provides supportive cellular and tissue evidence obtained under basal conditions: (i) the URM1 activity-based probe labels NAE1/UBA3 and UBE2M from cell and mouse tissue lysates without exogenous oxidative stimulation, and (ii) siNAE1/siUBA3/siUBE2M knockdown in intact cells under basal conditions reduces the overall URM1-conjugate signal detected by anti-URM1 immunoblotting. While we fully agree these remain indirect and do not substitute for direct flux measurements, they provide functional support that the neddylation machinery participates in URM1-related chemistry in a native cellular context.

Consistent with the reviewer's broader point, we have accordingly softened and clarified the framing throughout the manuscript: establishing URM1 as a bona fide physiological/cognate substrate in a canonical E1-E2-E3 cascade will require substantial future work, including direct detection of endogenous thioester intermediates in cells, quantitative flux measurements, and rigorous competition against NEDD8 under native conditions, as well as identification/validation of the relevant E3 components.

<u>For the reviewer's convenience, we provide the exact revised manuscript text:</u>

(Introduction) Through proteomic analysis and subsequent validation, we provide evidence that URM1 activation/transfer in human cells can be routed through the NAE1/UBA3-UBE2M axis, particularly under oxidative stress, supporting enzymatic access of URM1 to the neddylation machinery.

(Discussion) Although the functional experiments presented here are necessarily initial, they support a cytoprotective role of URM1 under oxidative stress and motivate future substrate-mapping and in vivo genetic studies to define physiological contexts where this pathway is most critical.

(Discussion) ..given the robust induction of URM1 conjugation by oxidative stress, an

important open question is whether stress-induced urmylation is universally routed through the NAE1/UBA3-UBE2M cascade or whether additional mechanisms contribute under specific contexts. Dissecting the relative contributions of these mechanisms under defined stress or developmental conditions will require future mechanistic studies.

(Discussion) Overall, our results identify NAE1/UBA3 and UBE2M as an E1/E2 activation cascade for URM1 and implicate DCN1 as an E3-like cofactor/scaffold that promotes URM1 conjugation under oxidative stress in human cells.

3) Additionally, no side-by-side NEDD8 controls are included, making it impossible to benchmark URM1 charging efficiency against the native substrate of this E1–E2 pathway. Given that the in vitro system has been established, a minimal and informative experiment would be to include URM1 and NEDD8 at equal concentrations in parallel E1 charging and E2 transfer assays, as well as in direct competition assays, to determine whether URM1 can meaningfully compete with NEDD8 for activation and transfer.

We thank the reviewer for this helpful suggestion. We agree that side-by-side NEDD8 benchmarking and direct competition provides essential context for interpreting URM1 engagement of the NAE1/UBA3–UBE2M system.

To address this point, we explicitly present and emphasize these NEDD8 controls in Figure S8, and we revised the Figure S8 legend and the corresponding main-text description (red highlights) to make the experimental design and interpretation unambiguous. In brief, Figure S8 includes parallel E1 charging and E2 transfer assays with URM1 and NEDD8-GG at matched concentrations, as well as a direct competition format (co-addition at reaction initiation). Across these formats, NEDD8-GG reduces the URM1-intermediate signals, consistent with the expectation that both Ubls can access this enzymatic route under defined in vitro conditions.

For the reviewer's convenience, we provide the exact revised manuscript text:

(Results) Finally, excess NEDD8-GG reduced formation of both intermediates in these in vitro reactions (Figure S8), suggesting that NEDD8 and URM1 can engage the same E1/E2 enzymes when both C-terminal diglycine substrates are present. We note that this in vitro competition reflects enzyme–substrate compatibility under defined conditions and does not necessarily imply direct competition between endogenous NEDD8 and URM1 in cells, where relative abundance, compartmentalization, and regulatory context can be fundamentally different.

(Discussion) Given the abundance of cellular NEDD8, we do not infer global competition between URM1 and NEDD8 in vivo under basal conditions. Substrate or channel selection between neddylation and urmylation may depend on stress-regulated availability of activated URM1 species, adaptor/E3 usage, and compartmentalization, variables that will require dedicated mechanistic dissection.

We sincerely thank the reviewer for the many thoughtful and technically rigorous suggestions provided across both rounds of review. We have continued to refine the manuscript accordingly and have pursued additional experiments, where feasible, to further strengthen URM1-specific interpretation.

We would like to report an additional control that addresses a key point raised in the first-round review regarding the possibility that perturbations of the neddylation pathway might indirectly alter the anti-URM1 signal via broader effects on proteostasis or redox balance. As suggested, we tested whether URM1-associated conjugate signals persist upon depletion of the native substrate NEDD8.

Specifically, we performed siRNA-mediated NEDD8 knockdown and assessed global URM1 conjugates by anti-URM1 immunoblotting under basal (-diamide) and oxidative stress (+diamide) conditions. Efficient NEDD8 depletion was confirmed by anti-NEDD8 immunoblotting, with comparable loading verified by GAPDH. Under these conditions, NEDD8 depletion did not diminish the overall anti-URM1 conjugate pattern; under oxidative stress conditions, the URM1 signal showed a trend toward higher intensity relative to the control. Although this represents an initial experiment, the result is consistent with the conclusion that the anti-URM1 signal we track is not simply attributable to NEDDylation-derived bands. We interpret this control conservatively and agree that definitive establishment of a canonical physiological E1-E2-E3 cascade for URM1 will require further dedicated work beyond the scope of the current revision.

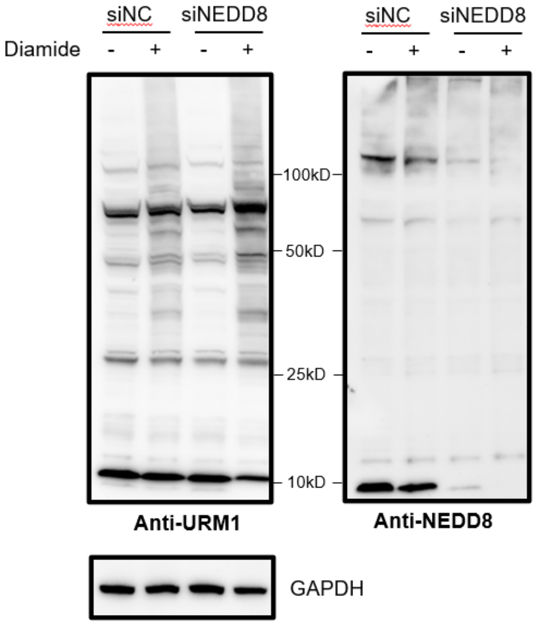

Figure. NEDD8 knockdown does not reduce global URM1 conjugate signals under basal or oxidative-stress conditions. HEK293T cells were transfected with non-targeting control siRNA (siNC) or siNEDD8 (IDT) using Lipofectamine™ RNAiMAX at a final siRNA concentration of 60 nM. At 36 h post-transfection, cells were treated with diamide (400 µM, 10 min) or left untreated, then harvested and lysed for immunoblotting. Global URM1 conjugates were detected by anti-URM1 immunoblotting, NEDD8 knockdown was confirmed by anti-NEDD8, and GAPDH served as a loading control.

Reviewer #3

The authors have revised the comments raised by this referee and to best of my assessment the comments by the other referees. The MS now can be accepted for publication in Nat Com.

Thank you for this positive assessment. We greatly appreciate the reviewer's time and careful evaluation of our revision.
